# Organogel delivery vehicles for the stabilization of organolithium reagents

**DOI:** 10.1038/s41557-023-01136-x

**Published:** 2023-02-16

**Authors:** Petr Slavík, Benjamin R. Trowse, Peter O’Brien, David K. Smith

**Affiliations:** Department of Chemistry, University of York, York, UK

**Keywords:** Molecular capsules, Reactive precursors, Synthetic chemistry methodology

## Abstract

Organolithium reagents are a vital tool in modern organic chemistry, enabling the synthesis of carbon–carbon bonds. However, due to their high reactivity, low temperatures, inert atmospheres and strictly dried solvents are usually necessary for their use. Here we report an encapsulating method for the stabilization of sensitive organolithium reagents—PhLi, *n*-BuLi and *s*-BuLi—in a low-cost hexatriacontane (C_36_H_74_) organogel. The use of this technology is showcased in nucleophilic addition reactions under ambient conditions, low-temperature bromine–lithium exchange, *ortho*-lithiation and C–H functionalization. The gel substantially enhances organolithium stability, allows simple storage, handling and delivery, and enables reproducible reagent portioning. The use of gels as easily divided delivery vehicles for hazardous organometallics has the potential to transform this area of synthetic chemistry, making these powerful reactions safer and more accessible to non-specialist researchers, and enabling the more widespread use of these common synthetic methods.

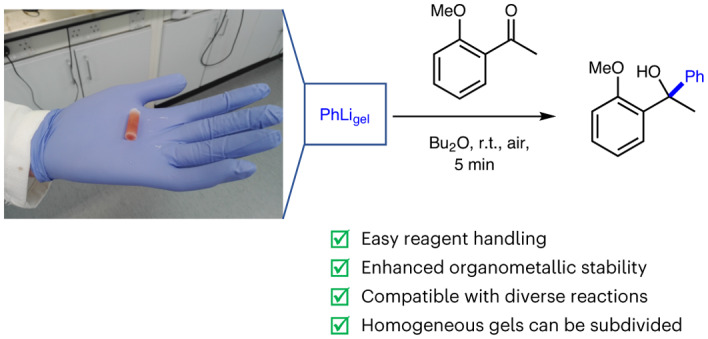

## Abstract (web summary)

Organolithium compounds are important reagents but are often hazardous due to their high reactivity. Now, encapsulating organolithium reagents within a supramolecular organogel has been found to enhance their stability, facilitating their storage and handling under ambient conditions. The homogeneous gels can be easily subdivided and dosed into a wide range of synthetic reactions.

## Main

A wide range of reactions in synthetic organic chemistry use hazardous reagents, many of which are unstable or pyrophoric in water or air and require handling under inert conditions. Organolithium reagents constitute one of the most important classes of such reagents and are widely used to construct carbon–carbon bonds^1,2^. Most notably, organolithiums are industrially applied in the synthesis of pharmaceuticals^3,4^ and the production of elastomer polymers^5^. There have been important developments in these simple but reactive systems in asymmetric synthesis^2,3,6–8^, which has added substantially to their synthetic value. However, it remains necessary to store and use these reagents under inert conditions. There are problems associated with reagent decomposition under extended storage, and reactions must be set up under rigorously inert conditions^9,10^. These issues introduce additional costs and safety hazards into the use of organolithium chemistry in industry, and also limit the extent to which non-specialist researchers use these methods. A simple and effective way of storing organolithiums for extended periods, as well as dosing reactions on demand, would be highly desirable. Indeed, there is considerable general interest in innovating the chemistry laboratory to empower the next generation of chemists^11^.

**Fig. 1:**
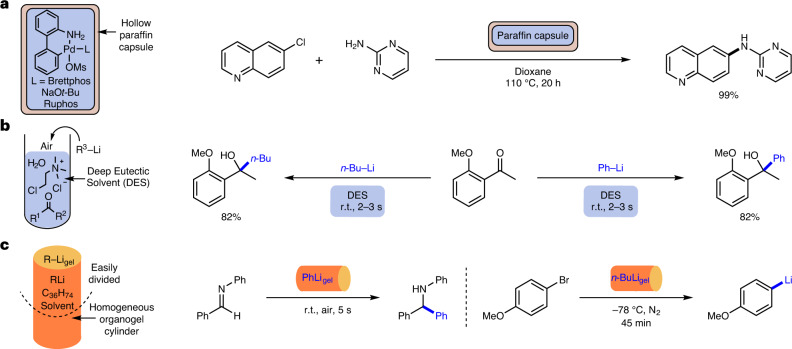
Technologies for the delivery of reactive species into organic reactions. **a**, Previous work on ‘drill and fill’ paraffin capsules developed as a single-dose delivery system and exemplified here with encapsulated palladium reagents and pre-catalysts for a Buchwald–Hartwig amination by Buchwald and others^12^. **b**, Previous work using deep eutectic solvents as a liquid delivery system to limit the hydrolysis of organolithium reagents as employed by Hevia and colleagues and exemplified here with a nucleophilic addition reaction^29^. **c**, The approach in this work using an organogel as a homogeneous easily divided delivery system for organolithium reagents, exemplified with reactions such as nucleophilic additions and Br–Li exchange reactions.

Consideration has previously been given to stabilizing other reactive species. One of the most important contributions was described by Buchwald and colleagues. They produced paraffin capsules, mechanically removed their cores, then loaded the capsules with air- and moisture-sensitive palladium (pre-)catalysts and metal salts, before resealing them^12^. The capsules could be removed from the glove box, safely handled in ambient conditions, and used to dose the reagents into cross-coupling reactions (Fig. 1a). Other researchers applied this approach to a variety of reactions^13–17^, including the use of potassium hydride^18^. Paraffin reagent capsules also have the benefit of protecting other species in multi-step reactions from catalyst poisoning and undesired side reactions, making product purification easier^19^. There has also been interest in using waxes to deliver reagents and catalysts into reactions^20,21^. In related work, but with a focus on toxic and malodorous reagents, Richert and colleagues reported a crystalline reagent coating based on tetrakis(dimethoxyphenyl)adamantane^22^. Xu and colleagues used a tableting approach based on mixing poly(tetrafluoroethylene) and crosslinked poly(styrene) with solid reagents, coating them with the same polymer mix to enhance stability^23^. Braje and co-workers recently made use of hydroxypropyl methylcellulose capsules to stabilize and simultaneously deliver catalyst, ligand and base into cross-coupling reactions for high-throughput screening^24^. Hydrophobic pockets formed by a cellulose derivative in water have also been used to facilitate catalytic reactions^25^. Teflon magnetic capsules have been developed for reagent delivery, with the stirring rate controlling the process^26^. Meanwhile, when working at process scale, dissolvable ‘SecuBags’ can deliver reactive reagents such as sodium hydride^27^.

With specific regard to organolithium reagents, the Hevia group reported that deep eutectic solvents (DESs) can deliver organolithiums into a range of reactions, minimizing hydrolysis and other side reactions (Fig. 1b)^28–31^. Capriati and colleagues performed Pd-catalysed cross-coupling reactions using organolithiums in aqueous conditions, albeit the reactions were very fast (reaction times of <1 min)^32^. Bergbreiter and co-workers reported that organolithium reagents could be used in poly(α-olefin) solvents, which have branched hydrocarbon structures, demonstrating enhanced stability and lower flammability^33,34^.

In the search for alternative ways of handling and dosing organolithium reagents, we reasoned that low-molecular-weight organogelators (LMWGs) may facilitate the generation of a gel-encapsulated organolithium that could be handled without the need for special procedures. Organogelators self-assemble into nanostructured materials at relatively low loadings in organic solvents via non-covalent interactions^35–37^. The resulting gels combine the solid-like behaviour of the nanostructured network with the liquid-like characteristics of the solvent^38^. Gels allow diffusion of soluble small molecules and are interesting media to support catalysts and to carry out reactions^39–43^. Incorporating an organolithium reagent into a gel could provide it with solid-like handling characteristics, and potentially protect the reactive species from air and water, offering stabilization and improved ease-of-handling. Furthermore, it should be possible to easily subdivide such gels, which is not possible with previously developed capsules or tablets.

To develop such a reagent dosing system, a gelator is required that is stable in the presence of an organolithium and compatible with the organic solvents in which they are used. Clearly, the gelator should not contain acidic protons, but, because many LMWGs self-assemble as a result of hydrogen-bond interactions^44^, this is a considerable design constraint. We focused our attention on a low-cost, linear alkane LMWG, C_36_H_74_ (hexatriacontane). Organogels comprising such long-chain alkanes have previously been described—they form networks of lamellar platelet-type aggregates^45,46^. Such organogels have not been exploited in applications other than superhydrophobic surfaces^47^ or coatings^48^. Indeed, they are best known as the troublesome aggregates that can form in oil pipelines caused by small quantities of these higher alkanes^49^. Our design principle was to repurpose higher alkanes such as C_36_H_74_ as delivery vehicles for reactive organolithiums. In this Article we report the successful implementation of hexatriacontane (C_36_H_74_) as an organogelator for commercial solutions of PhLi, *n*-BuLi and *s*-BuLi, as well as the use of these easily divided, easy-to-handle and air-/moisture-stable organolithium gels in reactions of industrial and academic relevance (Fig. 1c).

## Results and discussion

To begin, we studied the minimum gelation concentration of C_36_H_74_ in dibutyl ether and hexane—the solvents most commonly used for organolithium storage. In both cases, stable gels were obtained after a simple heat–cool cycle at concentrations of ~3% wt/vol. The *T*_gel_ values were 35–55 °C depending on gelator concentration. These relatively low *T*_gel_ values are desirable for organolithium delivery vehicles as they enable easy thermal processing to form (and break down) the gels. As expected, the gels exhibited lamellar platelet-type aggregates when imaged by scanning electron microscopy (Supplementary Figs. 1–3)^45,46^.

**Fig. 2:**
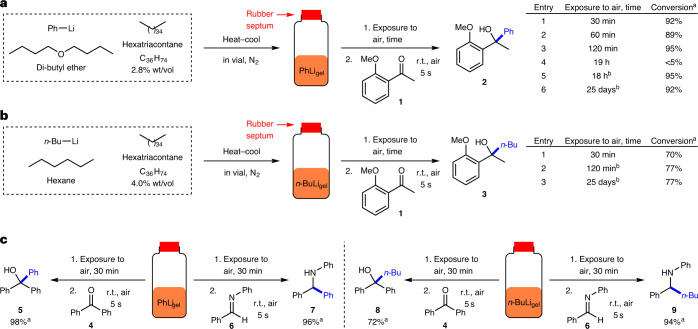
Evaluation of organolithium gels in vials using nucleophilic addition reactions under a variety of gel storage conditions. **a**, Formation of PhLi_gel_ and screening of PhLi_gel_ stability under ambient conditions, using reactivity in a nucleophilic addition to a ketone as the probe. The gel samples were stored under different conditions. **b**, Formation of *n*-BuLi_gel_ and screening of *n*-BuLi_gel_ stability under ambient conditions, using reactivity in a nucleophilic addition to a ketone as the probe. The gel samples were stored under different conditions. **c**, Nucleophilic addition reactions of PhLi_gel_ (left) and *n-*BuLi_gel_ (right) with a ketone and an imine, after exposure to air for 30 min, to yield secondary alcohol and amine products. ^a^Conversions were determined by ^1^H NMR spectroscopy using the relative integrals of key signals in the product and starting material. ^b^The vial was closed with a lid after 5-min exposure to air—this substantially extended the lifetime of the gel.

To prepare an organolithium-loaded gel, a simple procedure was developed that combined the C_36_H_74_ gelator, organolithium reagent (from a commercial solution) and additional solvent (Fig. 2a,b). An oven-dried vial (7 ml) with stirrer bar was charged with the C_36_H_74_ gelator, closed with a rubber septum and flushed with nitrogen. Anhydrous, degassed solvent (dibutyl ether for PhLi or hexane for *n*-BuLi) was added, followed by the organolithium (PhLi in dibutyl ether or *n*-BuLi in hexane). The vial was gently heated under nitrogen until the gelator dissolved, and was then immediately placed in an ice-water bath until the organogel formed. In each case, using 2.8–4.0% wt/vol of C_36_H_74_ gelator, a stable gel with incorporated organolithium (PhLi_gel_ and *n*-BuLi_gel_) was obtained in a vial.

Encouraged by the successful formation of organolithium gels in vials, their stability and reactivity under ambient conditions were evaluated. Initially, PhLi_gel_ (2 equiv.) was prepared and used in a nucleophilic addition reaction with 2′-methoxyacetophenone **1** (Fig. 2a, 0.8 mmol scale), the reaction chosen by Hevia and colleagues^29^ to study organolithium reagents in deep eutectic solvents. In all examples, the gel was exposed to the air for the specified time, after which the reagent was placed on top of the gel in the vial. After rapid stirring for only 5 s, which led to mechanical breakdown of the gel, the mixture was extracted, worked up and analysed by ^1^H NMR spectroscopy to determine the conversion to product. The gel network enhances organolithium stability under ambient conditions. Exposure of the gel to air under ambient conditions for 30 min and subsequent reaction with **1** resulted in 92% conversion to **2** (entry 1, Fig. 2a). In contrast, when commercial PhLi solution in dibutyl ether (1.9 M) was placed in the vial and exposed to ambient air for 30 min, only traces of **2** were observed. Prolonging the gel exposure time to 120 min did not cause much loss in PhLi activity (entries 2 and 3). However, overnight (19 h) exposure resulted in partial evaporation of the solvent (dibutyl ether) and damage to the gel network (Supplementary Fig. 5), together with decomposition of PhLi, as evidenced by <5% conversion to **2** (entry 4). This solvent evaporation problem was addressed by closing the vial with a lid. As a result, PhLi_gel_ that was exposed to ambient air for 30 min and then stored in a closed vial overnight (18 h) showed excellent activity, with 95% conversion to **2** (entry 5). Pleasingly, after much longer storage in a closed vial (25 days) under ambient conditions, the degradation of PhLi inside the gel remained negligible, and 92% conversion to **2** was obtained (entry 6). The gel formulation therefore provides long-term stabilization of PhLi, without storage at low temperature under an inert atmosphere.

Using a similar approach, *n*-BuLi was incorporated into a C_36_H_74_ gel. The stability and reactivity of *n*-BuLi_gel_ (2 equiv.) under ambient conditions was also evaluated by reaction with 2′-methoxyacetophenone **1** (Fig. 2b). The results suggest that *n*-BuLi is also stabilized within the gel and decomposition is limited (Supplementary Table 2). Indeed, conversion to **2** after 25 days of storage in a closed vial under ambient conditions (77%, entry 3, Fig. 2b) was slightly higher than the reaction using commercial *n*-BuLi solution under an inert atmosphere (70%). It was also shown that PhLi and *n*-BuLi organogels in vials could be successfully used for nucleophilic additions to benzophenone **4** and *N*-benzylideneaniline **6** (Fig. 2c).

**Fig. 3:**
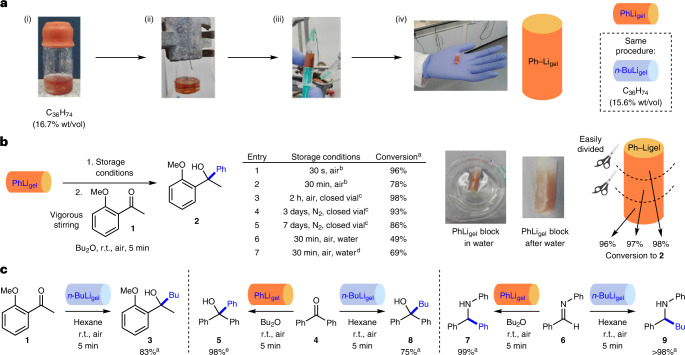
Formation, stability and reactivity of gel blocks incorporating PhLi and *n*-BuLi. **a**, Formation of the PhLi_gel_ block: (i) an oven-dried vial, containing PhLi, Bu_2_O, C_36_H_74_ (16.7% wt/vol) and a N_2_ balloon; (ii) gentle heating (heat gun) is applied until the gelator dissolves (under N_2_); (iii) the warm hydrosol is transferred to a syringe (under N_2_); (iv) after cooling (ice water), the gel is removed from the syringe by cutting to give the PhLi_gel_ block, which is stable to handling in a gloved hand. **b**, Stability of the PhLi_gel_ block under different storage conditions, which was tested using reactivity in a nucleophilic addition reaction as the probe, including an assessment of the divisibility of the PhLi_gel_ block. **c**, Nucleophilic addition reactions of PhLi_gel_ and *n*-BuLi_gel_ blocks with ketones and an imine to give secondary alcohols and amines. ^a^Conversions determined by ^1^H NMR spectroscopy using the relative integrals of key signals in the product and starting material. ^b^The PhLi_gel_ block was exposed to air on a Petri dish. ^c^The PhLi_gel_ block was transferred to a vial flushed with N_2_ and closed with a lid. ^d^The PhLi_gel_ block was formed using 33% wt/vol C_36_H_74_ gelator. ^e^Percent yield after purification by chromatography.

In the synthetic reactions in Fig. 2, reagents were placed on top of the organolithium gel, which had been formed within a vial. However, for general and practical use, we next prepared gel ‘blocks’ loaded with a specified amount of organolithium that could be directly and easily transferred under ambient conditions to another reaction vessel (such as a round-bottomed flask) containing the other reagents. To achieve this, the organolithium gel-formation procedure was modified (Supplementary General Procedure F and Methods), enabling the gel to be prepared inside a plastic syringe and then removed (Fig. 3a for PhLi_gel_). Ultimately, increasing the gelator concentration from 2.8% wt/vol to 16.7% wt/vol and diluting the commercial PhLi solution to 0.6 M in dibutyl ether resulted in the formation of a PhLi_gel_ block that was mechanically stable under ambient conditions and could be easily transferred using gloves. Based on titration experiments, decomposition of the organolithium during gel preparation was negligible (Supplementary Table 6). It was also possible to incorporate *n*-BuLi into a gel block using a similar procedure (15.6% wt/vol C_36_H_74_ gelator; Supplementary General Procedure F and Methods). To test the long-term stability of these formulations, gels were prepared in vials and sealed with a screwcap and electrical tape. These gels were briefly exposed to air (<30 s) while changing the cap, and stored in a desiccator under nitrogen. The amount of PhLi and *n*-BuLi in the gels was determined by titration experiments using (+)-menthol with 2,2′-bipyridine as an indicator^50^. No degradation was observed over a 42-day period, indicating potential for long-term storage in a container.

We explored the stability and reactivity of the PhLi_gel_ block with 2′-methoxyacetophenone **1** under a variety of storage conditions (Fig. 3b, 0.475 mmol scale). After preparing the PhLi_gel_ block (2 equiv.) and storing for 30 s under ambient conditions in air (on a Petri dish), it was added to **1** in solvent. Upon stirring, the gel block broke down to release PhLi into the solution, and 96% conversion to **2** was achieved (Fig. 3b, entry 1). Storing for 30 min in air on a Petri dish before reaction led to a lower conversion (78%; entry 2). The PhLi_gel_ blocks were more sensitive to storage than gels in vials, presumably due to their greater surface area, with surface-located PhLi being more able to react with moisture in ambient air. However, a 2-h storage time in air in a closed vial restored high conversion (98%; entry 3), which was maintained over seven days under nitrogen in a closed vial (entries 4 and 5). Alternatively, storage of the PhLi_gel_ block in an inert atmosphere, by leaving it in the syringe with the needle blocked in a rubber stopper, effectively maintained its activity. Similar stability was observed for the *n*-BuLi_gel_ block (Supplementary Table 7).

To further demonstrate the protective ability, the PhLi_gel_ block was immersed in a beaker of water for 30 min (Fig. 3b). It was then removed, dried with a paper towel and directly used in a reaction with **1**. The conversion to **2** (49%; entry 6) was lower than the standard reaction, presumably because PhLi located at the surface of the block partly decomposes upon contact with water (as noted visually, Fig. 3b). However, the fact that the reaction still proceeds in reasonable yield is strong evidence of the protective effect of gel encapsulation. Obviously, PhLi added to water in the absence of gel protection is all rapidly destroyed. Increasing gelator loading to 33% wt/vol and performing the same experiment gave a more robust gel, improving conversion to 69% (entry 7). The PhLi_gel_ block could be further stabilized by coating it with an inert layer, to prevent surface decomposition; coating the PhLi_gel_ block in paraffin gave 83% conversion to **2** after exposure to water (Supplementary Fig. 43). However, we reason that there are substantial advantages of working with a gel block rather than a filled capsule.

A key design principle of our organolithium gels is that they have an even distribution of the reagent through the gel, unlike the ‘drill and fill’ capsule technology developed by Buchwald and colleagues (Fig. 1a)^12^. To prove the equal distribution of reagent, a PhLi_gel_ block was cut into three equal-sized pieces using a razor blade. Each piece was used in a reaction with **1** to give **2**, with conversions of 96%, 97% and 98% (Fig. 3b), demonstrating that it is possible to subdivide the organolithium gel blocks to facilitate reagent dosing. Titration of *n*-BuLi and PhLi within the divided gel blocks (20% wt/vol gelator) found essentially identical concentrations of organolithium in each piece of gel, indicating the homogeneous nature. The variability in the mass of pieces of gel cut in this way was 5–10%. For more accurate gel subdivision, it may be desirable to use an extrudable gel, held within a needle-free syringe, with the required volume being pushed out of the syringe when needed. In terms of application of the technology, these observations are crucial as they demonstrate that a single large batch of homogeneous organolithium gel can be produced (for example, by a chemical supply company), packaged, stored and shipped in an appropriate way, then subdivided into the amounts required by the end-user, and used in different reactions.

The PhLi_gel_ and *n*-BuLi_gel_ blocks performed very well in other nucleophilic additions. The organolithium gels were prepared and stored for 10 s in air on a Petri dish before use, and good conversions to products **3**, **5** and **7**–**9** (75–79%) were obtained from reactions with 2′-methoxyacetophenone **1**, benzophenone **4** and *N*-benzylideneaniline **6** (Fig. 3c). In general, most of the C_36_H_74_ gelator can be removed in the work-up by precipitation and filtration. Standard flash column chromatography can be used to remove any final traces, eluting first with hexane to remove C_36_H_74_ and then with 1:1 hexane–dichloromethane to elute product. A 98% isolated yield of alcohol **5** was thus obtained from a PhLi_gel_ block and benzophenone **4** (Fig. 3c). The same purification approach was used by Buchwald and colleagues to remove residual paraffin from reactions using their paraffin capsules^12^.

**Fig. 4:**
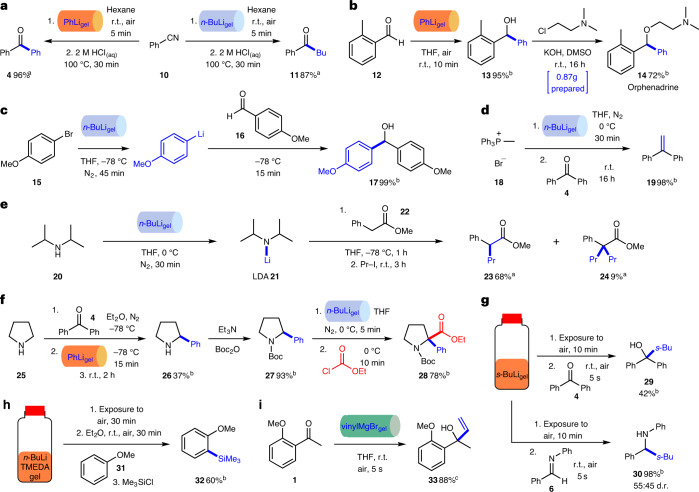
Synthetic applications of the PhLi_gel_ and *n-*BuLi_gel_ blocks, *s*-BuLi_gel_, *n*-BuLi/TMEDA_gel_ and vinylMgBr_gel_. **a**, Synthesis of ketones **4** and **11** via nucleophilic addition or organolithium gels to benzonitrile. **b**, Synthesis of 0.87 g of orphenadrine **14** using 9.5 mmol of a PhLi_gel_ block. **c**, Bromine–lithium exchange using an *n*-BuLi_gel_ block and addition of the organolithium generated to an aldehyde to give **17**. **d**, Wittig reaction to produce **19** using an *n*-BuLi gel block to create the ylid. **e**, Preparation of LDA **21** using an *n*-BuLi gel block, and its use in an enolate α-alkylation reaction. **f**, α-C–H bond difunctionalization of pyrrolidine using PhLi_gel_ and *n-*BuLi_gel_ blocks to give **28**. **g**, Nucleophilic addition reactions of *s*-BuLi_gel_ with a ketone and an imine to give **29** and **30**, respectively. **h**, The *ortho*-lithiation of methoxybenzene using a *n*-BuLi/TMEDA_gel_ followed by trapping with trimethylsilyl chloride to give **32**. **i**, Nucleophilic addition reaction of vinylMgBr_gel_ to a ketone to give **33**. ^a^Conversions determined by ^1^H NMR spectroscopy using dimethylformamide (DMF) as an external standard. ^b^Percent yield after purification by chromatography. ^c^VinylMgBr_gel_ exposed to air for 5 min before reaction; conversion determined by ^1^H NMR spectroscopy using the relative integrals of key signals in the product and starting material.

The PhLi_gel_ and *n*-BuLi_gel_ blocks were then used in a wider array of synthetic applications, including more challenging reactions (Fig. 4). Reaction of each of the PhLi_gel_ and *n*-BuLi_gel_ blocks with benzonitrile **10** followed by hydrolysis^51^ resulted in ketones **4** and **11** in 96% and 87% NMR yields, respectively (Fig. 4a). The preparation of a PhLi_gel_ block was scaled up (to 9.5 mmol of PhLi encapsulated; Supplementary Fig. 58) and used in the synthesis of 0.87 g of the anticholinergic and antihistamine drug orphenadrine **14**^52^. Reaction of PhLi_gel_ with 2-methylbenzaldehyde **12** under ambient conditions in air gave alcohol **13** in 95% isolated yield and, after *N*-alkylation with 2-(*N*,*N*-dimethylamino)ethylchloride, orphenadrine **14** was obtained in 72% yield (Fig. 4b). A bromine–lithium exchange reaction^53^ was performed with 4-bromoanisole **15** using an *n*-BuLi_gel_ block at −78 °C under an inert atmosphere followed by trapping the intermediate aryllithium with 4-methoxybenzaldehyde **16** to give alcohol **17** in 99% isolated yield (Fig. 4c). The *n*-BuLi_gel_ block was also successfully used to generate an ylid from phosphonium bromide **18**, and deployed in a Wittig reaction with benzophenone **4**^54^ to give alkene **19** in 98% isolated yield (Fig. 4d). The widely used strong base LDA **21** was prepared from diisopropylamine **20** and the *n*-BuLi_gel_ block, and then used in an enolate α-alkylation reaction of ester **22**^55^ to give **23** in 68% NMR yield (Fig. 4e).

We also successfully utilized both PhLi_gel_ and *n*-BuLi_gel_ blocks in a three-step, double α-C–H functionalization of pyrrolidine under inert conditions (Fig. 4f). In the first step, using Seidel’s approach^56^ with the PhLi_gel_ block, deprotonation of the NH proton in pyrrolidine **25** followed by hydride transfer to benzophenone **4** and addition of PhLi to the in situ-generated imine provided 2-phenylpyrrolidine **26** in 37% isolated yield. In our hands, using commercial PhLi solution, 2-phenylpyrrolidine **26** was obtained in 50% isolated yield. The 2-phenylpyrrolidine **26** was Boc-protected and then, following a method reported by O’Brien and Coldham^57^, benzylic lithiation using an *n*-BuLi_gel_ block at 0 °C and subsequent reaction with ethyl chloroformate gave α,α-disubstituted pyrrolidine **28** in 78% isolated yield.

To push the gel-protection technology further, we explored gels containing *s*-BuLi, a much more reactive organolithium. In this case, gels were formed in vials using a stock solution of *s*-BuLi (1.15 M in hexanes). The stability of the *s*-BuLi was determined by titration ((+)-menthol, 2,2′-bipyridine as indicator) and compared with *s-*BuLi solutions stored in the same way. For the *s-*BuLi solution, the amount of *s-*BuLi fell to 37% after 5 min exposure to air at ambient temperature, and 24% after 10 min. For *s*-BuLi_gel_ formed with 20% wt/vol gelator, the stability was considerably enhanced, with more *s*-BuLi remaining after exposure to air for 5 min (78%), 10 min (73%) and even 30 min (53%) at room temperature. It is evident that the gel network has a protective effect, although there is more degradation on exposure of *s*-BuLi_gel_ to air compared to PhLi_gel_ and *n*-BuLi_gel_. Two reactions using *s*-BuLi_gel_ in a vial that was exposed to air for 10 min were performed: reaction with benzophenone **4** gave alcohol **29** in 42% yield and with *N*-benzylideneaniline **6** gave amines **30** in 98% yield (Fig. 4g).

It was of interest to generate gels that also included a ligand for the organolithium. We therefore formed a gel incorporating both *n*-BuLi and tetramethylethylenediamine (TMEDA). The ligand activates the organolithium, and more gelator was therefore required to formulate a stable gel—25% wt/vol of gelator produced a gel in a vial that was stable to air for 30 min. This *n*-BuLi/TMEDA_gel_ was used for *ortho*-lithiation of methoxybenzene **31**^58^, and trapping with trimethylsilyl chloride giving silane **32** in 60% yield (Fig. 4h).

Finally, two organomagnesium (Grignard) reagents were incorporated successfully in gels. Specifically, we prepared gels based on vinylmagnesium bromide (0.7 M in tetrahydrofuran (THF)) with a gelator loading of 10% wt/vol, and phenylmagnesium chloride (1.0 M in 2-MeTHF) at gelator loadings of 5–10% wt/vol. The organomagnesium gel blocks were sufficiently robust to be prepared in syringes, removed, and transferred to a reaction vessel even at these relatively low gelator loadings. Reaction of vinylMgBr_gel_ with 2′-methoxyacetophenone **1** worked well, giving 88% conversion to alcohol **33** after exposure to air for 5 min (Fig. 4i). Hevia and colleagues reported a 77% yield of alcohol **33** for this reaction performed in deep eutectic solvents^29^. The PhMgCl_gel_ blocks were titrated and shown to have ~80% of the theoretical amount of PhMgCl present after 1-min exposure to air on a Petri dish. It is anticipated that higher gelator loadings will further enhance the stability of these organomagnesium gel blocks.

In summary, sensitive organolithium reagents such as PhLi, *n*-BuLi and *s*-BuLi can be successfully incorporated within C_36_H_74_ organogel delivery vehicles. The gel network provides considerable stability towards ambient conditions, and these organolithium gels can therefore be used without the need for many of the special working protocols usually needed for this type of chemistry. Our approach, using a small amount of a readily available, low-cost gelator (C_36_H_74_) in an organic solvent, has advantages, including solvent compatibility, simple manufacture and even distribution of the reagent through the gel for effective subdivision and accurate reaction dosing. The use of gels as delivery vehicles for hazardous organometallics has the potential to transform this area of synthetic chemistry, making these powerful reactions safer and more accessible, and enabling their more widespread use. We anticipate that gels could, for example, be commercially supplied as individual doses wrapped in foil, or in syringe tubes that enable extrusion of the required amount of gel. We also perceive benefits in using this simple technology for long-term storage and/or shipping of bulk organolithiums in industry. It is anticipated that this simple gel-mediated approach to reagent stabilization could be applied to other moisture- and air-sensitive reagents—this has been demonstrated here for organomagnesiums, and further work is currently in progress.

## Methods

Key methods for the preparation of organolithium gels in vials and as mechanically robust gel blocks, and their use in a standard test reaction, are presented here. Full details of all methods and all reactions are provided in the Supplementary Information.

### Preparation of organolithium gels (PhLi_gel_ and *n-*BuLi_gel_) in a vial

A 7-ml vial with stirrer bar was dried in an oven and allowed to cool under a nitrogen atmosphere. The vial was charged with the gelator C_36_H_74_ (80.0 mg, 0.16 mmol, 2.8% wt/vol in the case of PhLi and 4.0% wt/vol in the case of *n-*BuLi), sealed with a rubber septum and flushed with nitrogen via a needle for 5 min. Anhydrous and degassed solvent (2 ml of dibutyl ether in the case of PhLi or 1 ml of hexane in the case of *n*-BuLi) was added through the septum, followed by addition of the organolithium reagent (0.84 ml of PhLi, 1.91 M in dibutyl ether or 1 ml of *n*-BuLi, 1.6 M in hexane). The vial (kept under a nitrogen atmosphere using a balloon) was carefully heated until all of the gelator had dissolved. The vial was then immediately placed in iced water for 1 min until the organogel formed (Supplementary Fig. 4).

### Reaction of PhLi_gel_ in a vial with 2′-methoxyacetophenone 1

PhLi_gel_ (1.6 mmol) was prepared in a vial according to the above method. The organolithium gel was exposed to air by removing the rubber septum. After the specified time (Supplementary Table 1), 2′-methoxyacetophenone 1 (110.4 µl, 0.8 mmol) was added on top of the gel at room temperature and under air. The mixture was vigorously stirred for 5 s before the reaction was quenched by the addition of water (0.5 ml). The solids in the reaction mixture were removed by filtration using a glass funnel and filter paper. This procedure removed most of the C_36_H_74_ gelator. The reaction vial and filter paper with the gelator were washed with additional dibutyl ether (3 × 2 ml), and the combined filtrates were dried (MgSO_4_) and evaporated under reduced pressure to give the crude product. The crude product was analysed by ^1^H NMR spectroscopy to determine the conversion based on relative integrals of the CH_3_ group in the product and starting material.

### Preparation of mechanically robust organolithium gel (PhLi_gel_ and *n*-BuLi_gel_) blocks

A 5-ml vial was dried in the oven and allowed to cool under a nitrogen atmosphere. The vial was charged with the gelator C_36_H_74_ (250.0 mg, 0.49 mmol, 16.7% wt/vol in the case of PhLi and 15.6% wt/vol in the case of *n-*BuLi), sealed with a rubber septum and flushed with nitrogen three times. Anhydrous and degassed solvent (1 ml of dibutyl ether in the case of PhLi or 1 ml of hexane in the case of *n*-BuLi) was added through the septum followed by the addition of the organolithium reagent (0.50 ml of PhLi, 1.91 M in dibutyl ether or 0.6 ml of *n*-BuLi, 1.6 M in hexane). The vial (kept under a nitrogen atmosphere using a balloon) was carefully heated until all of the gelator had dissolved. The hot hydrosol was quickly transferred under a nitrogen atmosphere via a needle into a 2-ml syringe (previously flushed with nitrogen and pre-heated in the oven). The syringe was immediately placed in iced water for 1 min until the organogel formed. The organolithium gel was kept in the syringe under a nitrogen atmosphere before use. To use the organolithium gel, the upper part of the syringe was carefully cut with scissors and the gel block was removed (Supplementary Fig. 28).

### Reaction of the PhLi_gel_ gel block with 2′-methoxyacetophenone 1

A PhLi_gel_ block (0.95 mmol) was prepared according to the general procedure described above. After the specified time and storage conditions (Supplementary Table 8), the gel block was carefully placed in a 5-ml round-bottomed flask containing a solution of 2′-methoxyacetophenone **1** (65.4 µl, 0.475 mmol) in dry dibutyl ether (2 ml) at room temperature and under air. The mixture was vigorously stirred for 5 min before the reaction was quenched by the addition of water (0.5 ml). The solids in the reaction mixture were removed by filtration using a glass funnel and filter paper. This procedure removed most of the C_36_H_74_ gelator. The reaction vial and filter paper with the gelator were washed with additional dibutyl ether (3 × 2 ml), and the combined filtrates were dried (MgSO_4_) and evaporated under reduced pressure to give the crude product. The crude product was analysed by ^1^H NMR spectroscopy to determine the conversion based on relative integrals of the CH_3_ group in the product and starting material.

## Online content

Any methods, additional references, Nature Portfolio reporting summaries, source data, extended data, supplementary information, acknowledgements, peer review information; details of author contributions and competing interests; and statements of data and code availability are available at 10.1038/s41557-023-01136-x.

## Supplementary information

Supplementary Information Supplementary Information, including all methods, all experimental data and Figs. 1–84 of additional information.

## Data Availability

Supplementary Information is available in the online version of the paper. It includes full details of gel fabrication and characterization, all details of organic reaction methods and supporting characterization data. All supporting spectroscopic data can be found in their original form in an open-access depository at 10.15124/959c1b7b-6e81-4461-8a89-1a1ee8ce0b9f.
