# Author Correction: Organogel delivery vehicles for the stabilization of organolithium reagents

**DOI:** 10.1038/s41557-023-01198-x

**Published:** 2023-04-06

**Authors:** Petr Slavík, Benjamin R. Trowse, Peter O’Brien, David K. Smith

**Affiliations:** Department of Chemistry, University of York, York, UK

**Keywords:** Molecular capsules, Reactive precursors, Synthetic chemistry methodology

Correction to: *Nature Chemistry* 10.1038/s41557-023-01136-x. Published online 16 February 2023.

In the version of this article initially published, two relevant articles were not cited: Malinski, J. & Bergbreiter, D. E. Safer solvents for organolithium reagents. *Tetrahedron Lett*. **59**, 3926–3929 (2018) and Thavornpradit, S., Malinski, T. J. & Bergbreiter, D. E. Applications of poly(α-olefins) as solvents in organometallic chemistry. *J. Organomet. Chem*. **962**, 122261 (2022). They have now been inserted as refs. 33 and 34 and the subsequent references renumbered. The new references and explanatory text have been included in the third-to-last paragraph of the Introduction, reading “Bergbreiter and co-workers reported that organolithium reagents could be used in poly(α-olefin) solvents, which have branched hydrocarbon structures, demonstrating enhanced stability and lower flammability^33,34^” in the HTML and PDF versions of the article.

