# Supplementary material for: Organogel delivery vehicles for the stabilization of organolithium reagents
Source: Nat Chem. 2023 Feb 16;15(3):319–25. doi: 10.1038/s41557-023-01136-x (PMC9986108; doi:10.1038/s41557-023-01136-x)
Supplement: Supplementary file 1 — Supplementary Information, including all methods, all experimental data and Figs. 1–84 of additional information. [file 41557_2023_1136_MOESM1_ESM.pdf]

nature chemistry

Article

https://doi.org/10.1038/s41557-023-01136-x

Organogel delivery vehicles for the stabilization of organolithium reagents

In the format provided by the authors and unedited

Table of Contents

1. General Experimental Methods
2. SEM Images of $C_{36}H_{74}$ Gels
3. Preparation of PhLi and *n*-BuLi Gels in a Vial and Subsequent Reactions (Results in Fig. 2)
4. Preparation of $PhLi_{gel}$ and *n*-$BuLi_{gel}$ Blocks and Subsequent Reactions (Results in Fig. 3)
5. Synthetic Applications of the $PhLi_{gel}$ and *n*-$BuLi_{gel}$ Blocks (Results in Fig. 4)
6. Preparation of *s*-BuLi Gel in a Vial and Subsequent Reactions (Results in Fig. 4)
7. Preparation of *n*-BuLi/TMEDA Gel in a Vial and Subsequent Reactions (Results in Fig. 4)
8. Preparation of $vinylMgBr_{gel}$ and $PhMgCl_{gel}$ Blocks and Subsequent Reactions (Results in Fig. 4)

1. General Experimental Methods.

All compounds required in synthesis and analysis were purchased from standard commercial suppliers: PhLi (Aldrich, 1.9 M in dibutyl ether), *n*-BuLi (Alfa Aesar, 1.6 M in hexane or Fisher Thermo Scientific, 1.6 M in hexanes), s-BuLi (Fisher Thermo Scientific, 1.3 M in 92:8 cyclohexane/hexane), hexatriacontane (Alfa Aesar, 97+%), benzophenone (Sigma Aldrich, 99%), 2′-methoxyacetophenone (Alfa Aesar, 98%), *N*-benzylideneaniline (Acros Organics, 99%), 2-methylbenzaldehyde (TCI, >98.0%), 4-bromoanisole (Aldrich, ≥99.0%), methyltriphenylphosphonium bromide (Aldrich, 98%), methyl 2-phenylacetate (Aldrich, ≥99 %), 2,2-bipyridine (Alfa Aesar, 99%), (+)-menthol (TCI, >99.0%), dibutyl ether (Sigma Aldrich, anhydrous 99.3%), hexane (Acros Organics, extra dry or from Pure Solv^{TM} Micro Solvent Purification System), toluene (Pure Solv^{TM} Micro Solvent Purification System), THF (Pure Solv^{TM} Micro Solvent Purification System). Pyrrolidine and Me_3SiCl were freshly distilled and stored under nitrogen prior to use. TMEDA (Aldrich) and anisole (Aldrich) were freshly distilled from CaH_2 stored under nitrogen prior to use. ^1H and ^{13}C NMR spectra were recorded on a JEOL ECX 400 (^1H 400 MHz, ^{13}C 101 MHz) spectrometer. Samples were recorded as solutions in deuterated NMR solvents as stated and chemical shifts (δ) are quoted in parts per million. SEM images were taken using a JEOL JSM-7600F field emission SEM.

Temperature control was not routinely used in synthetic reactions using organolitihum gels, which were performed on relatively small lab scale. However, it may be desirable during scale-up to prevent solvent boiling in these exothermic reactions. We also observed that in cases where the gel did not break down, organolithium release was slower than in cases where the gel broke down upon stirring with a magnetic stirrer bar (over a period of hours rather than minutes) as organolithium has to diffuse from the gel (or reagents diffuse in). In the absence of stirring, it may thus be possible to achieve slow reagent release; alternatively, under vigorously stirred conditions where the gel is broken down, rapid release is obtained. This may provide another way in which reaction outcomes can be controlled.

Unless stated otherwise, the concentration of organolithium reagents was determined by titration experiments using butan-2-ol with 1,10-phenanthroline as an indicator.[1]

2. SEM Images of $C_{36}H_{74}$ Gels.

SEM Images of $C_{36}H_{74}$ gels

Samples for SEM were obtained by air drying the gels on copper shim pieces. The air-dried samples were then mounted on stubs and the images recorded.

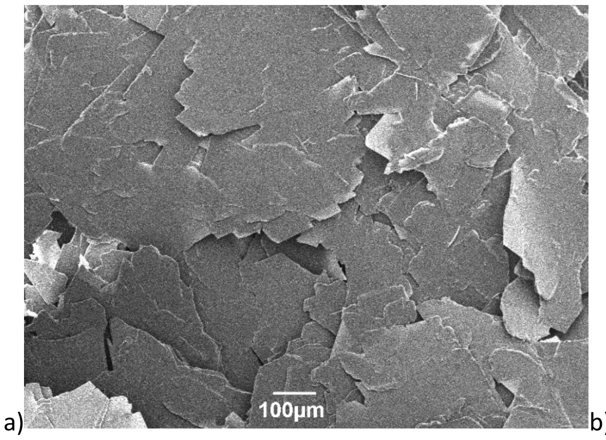

a)

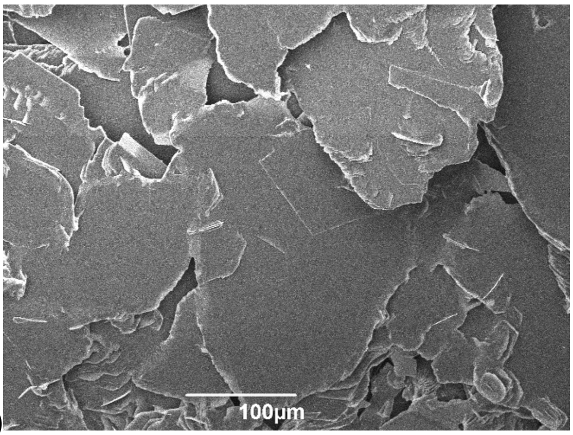

b)

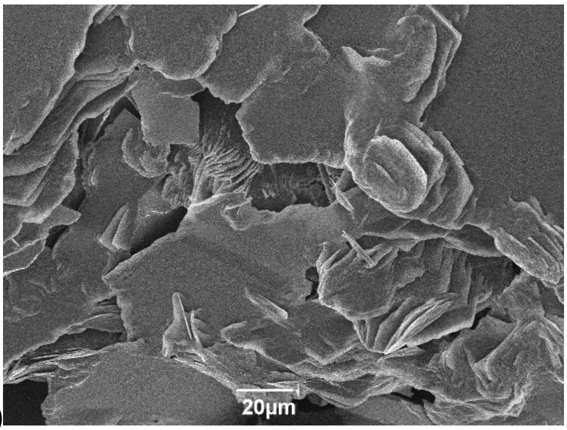

c)

Figure S1. SEM images of $C_{36}H_{74}$-dibutyl ether gel (10% wt/vol). Scale bars: a) 100 μm; b) 100 μm; c) 20 μm.

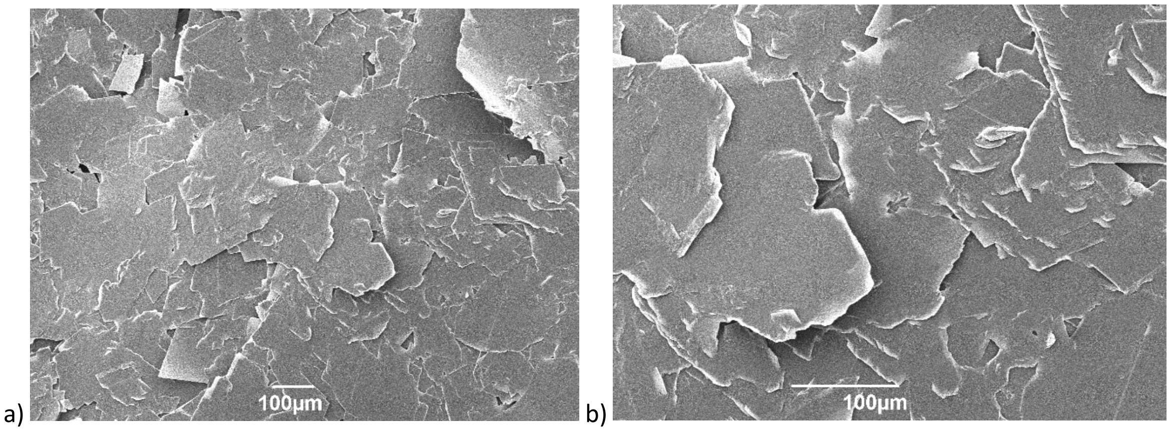

Figure S2. SEM images of $C_{36}H_{74}$-dibutyl ether gel (5% wt/vol). Scale bars: a) 100 µm; b) 100 µm.

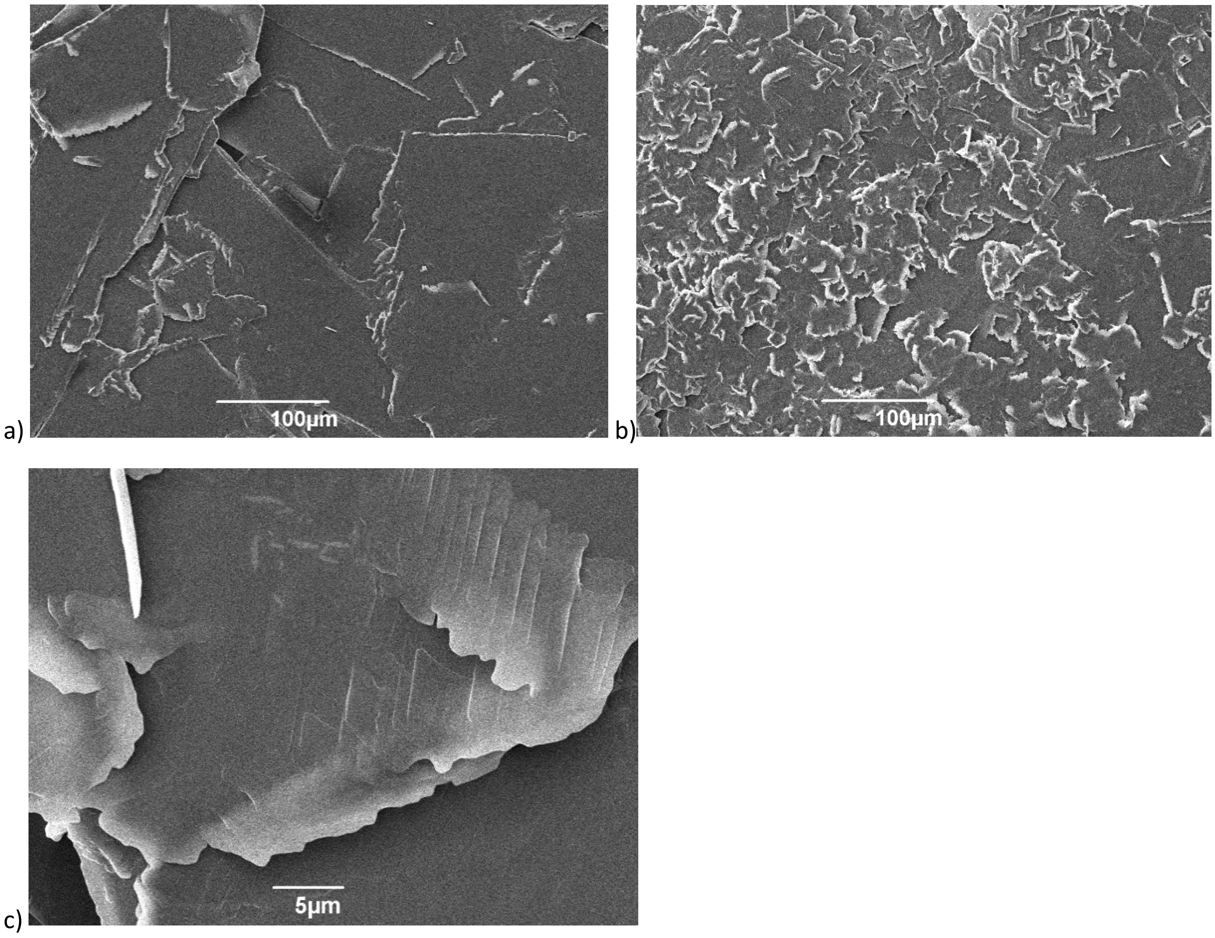

Figure S3. SEM images of $C_{36}H_{74}$-hexane gel (10% wt/vol). Scale bars: a) 100 µm; b) 100 µm; c) 5 µm.

3. Preparation of PhLi and *n*-BuLi Gels in a Vial and Subsequent Reactions (Results in Fig. 2)

General procedure A: preparation of the organolithium gels ($PhLi_{gel}$ and *n*-$BuLi_{gel}$) in a vial

A 7 mL vial with stirrer bar was dried in the oven and allowed to cool under a nitrogen atmosphere. The vial was charged with the gelator $C_{36}H_{74}$ (80.0 mg, 0.16 mmol, 2.8% wt/vol in the case of PhLi and 4.0% wt/vol in the case of *n*-BuLi), sealed with a rubber septum and flushed with nitrogen via a needle for 5 min. Anhydrous and degassed solvent (2 mL of dibutyl ether in the case of PhLi or 1 mL of hexane in the case of *n*-BuLi) was added through the septum followed by the addition of the organolithium reagent (0.84 mL of PhLi – 1.91 M in dibutyl ether or 1 mL of *n*-BuLi – 1.6 M in hexane). The vial (kept under a nitrogen atmosphere using a balloon) was carefully heated until all of the gelator had dissolved. Then, the vial was immediately placed in iced water for 1 min until the organogel formed. This is shown in Fig. S4.

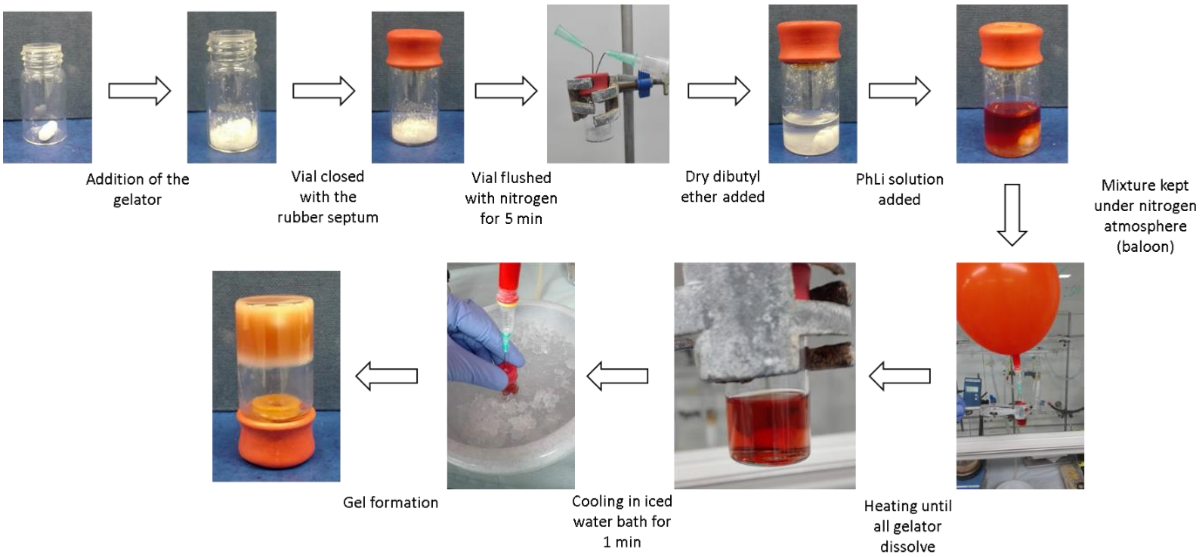

Figure S4. Schematic illustration of the preparation of $PhLi_{gel}$ in a vial.

General procedure B: Reaction of $PhLi_{gel}$ with 2′-methoxyacetophenone 1 using an organolithium gel in a vial

$PhLi_{gel}$ (1.6 mmol) was prepared in a vial according to general procedure A. The organolithium gel was exposed to air by removing the rubber septum. After the specified time (see Table S1), 2′-methoxyacetophenone **1** (110.4 µL, 0.8 mmol) was added on top of the gel at room temperature and under air. The mixture was vigorously stirred for 5 s before the reaction was quenched by the addition of water (0.5 mL). The solids in the reaction mixture were removed by filtration using a glass funnel and filter paper. This procedure removed most of the $C_{36}H_{74}$ gelator. The reaction vial and filter paper with the gelator were washed with additional dibutyl ether (3 x 2 mL) and the combined filtrates were dried ($MgSO_4$) and evaporated under reduced pressure to give the crude product. The crude product was analysed by ^{1}H NMR spectroscopy to determine the conversion based on relative integrals of the CH_3 group in the product and starting material. Compound **2** has been previously reported and the spectroscopic data were in agreement.[2]

Table S1 Screening of $PhLi_{gel}$ stability under ambient conditions (Fig. 2A).

Entry	Exposure to Air Time	Conversion to **2**[a]
1	30 min	92%
2	60 min	89%
3	120 min	95%
4	19 h	<5%
5	18 h[b]	95%
6	25 days[b]	92%

[a] Conversions determined by ^{1}H NMR spectroscopy using the relative integrals of key signals in product and starting material. [b] The vial was closed with a lid after 30 min exposure to air.

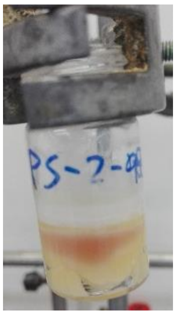

Fig S5. $PhLi_{gel}$ after exposure to air for 19 h in which the vial was not closed with a lid (used in reaction in Table S1, entry 4).

Reaction of $PhLi_{gel}$ with 2′-methoxyacetophenone 1 using $PhLi_{gel}$ in a vial: ^{1}H NMR Spectra

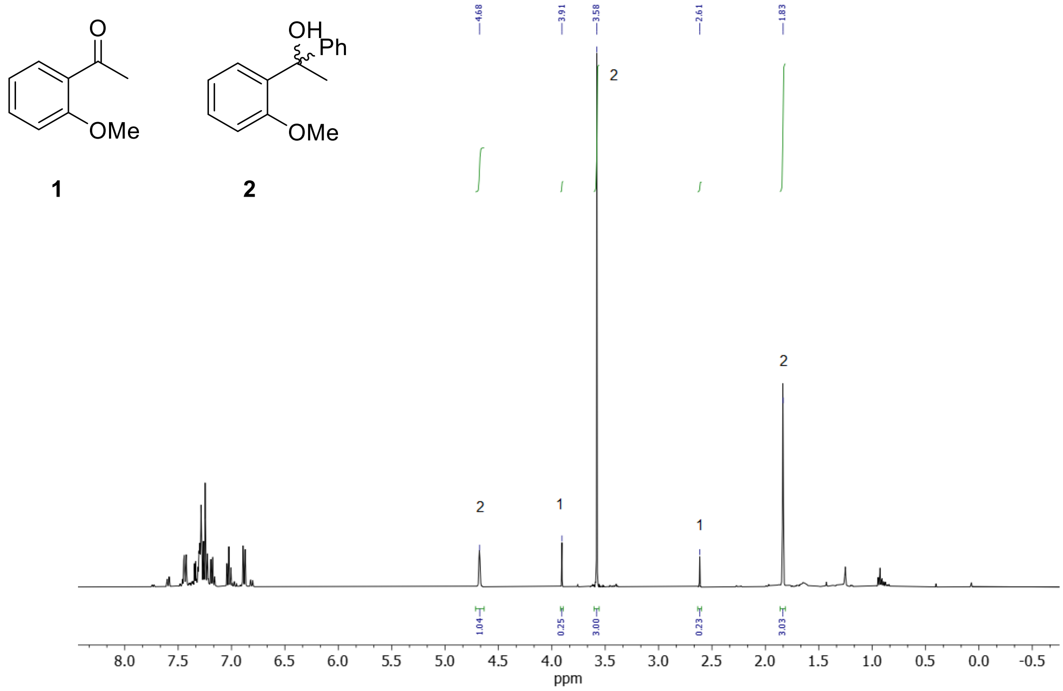

Figure S6. ^{1}H NMR spectrum of the crude product obtained after exposure of $PhLi_{gel}$ to air for 30 min followed by the reaction with 2′-methoxyacetophenone **1** ($CDCl_3$, 298 K, 400 MHz) – Fig. 2A, Entry 1 (Table S1).

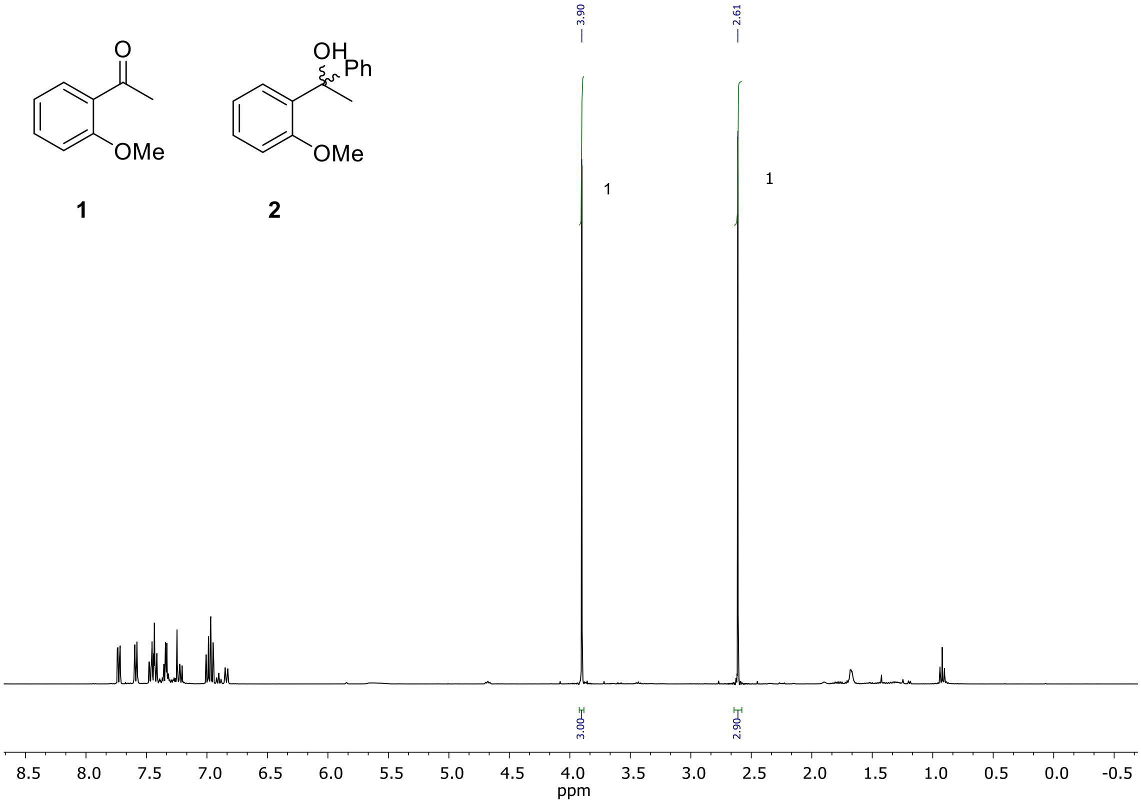

Figure S7. ^{1}H NMR spectrum of the crude product obtained after exposure of PhLi solution to air for 30 min followed by the reaction with 2′-methoxyacetophenone **1** ($CDCl_3$, 298 K, 400 MHz) – see text in paper.

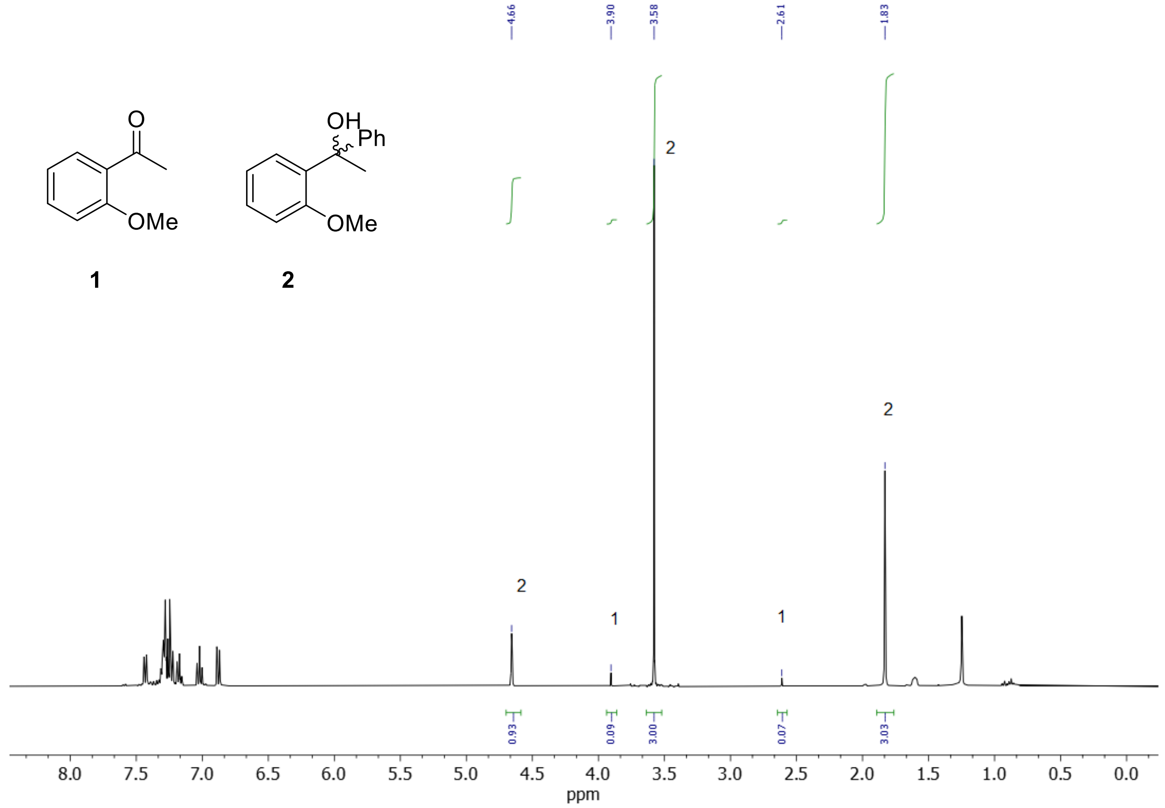

Figure S8. ^{1}H NMR spectrum of the crude product obtained after exposure of $PhLi_{gel}$ to air for 60 min followed by the reaction with 2′-methoxyacetophenone **1** ($CDCl_3$, 298 K, 400 MHz) – Fig. 2A, Entry 2 (Table S1).

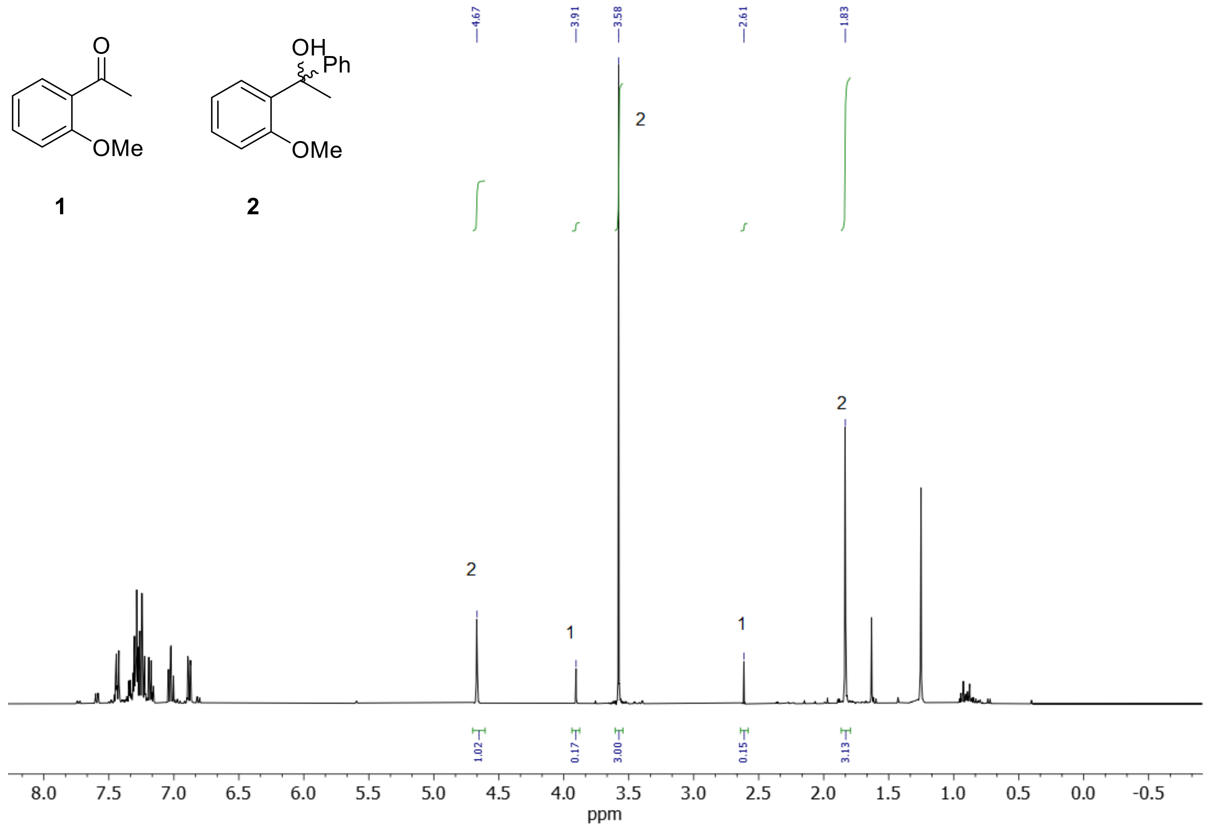

Figure S9. ^{1}H NMR spectrum of the crude product obtained after exposure of $PhLi_{gel}$ to air for 120 min followed by the reaction with 2′-methoxyacetophenone **1** ($CDCl_3$, 298 K, 400 MHz) – Fig. 2A, Entry 3 (Table S1).

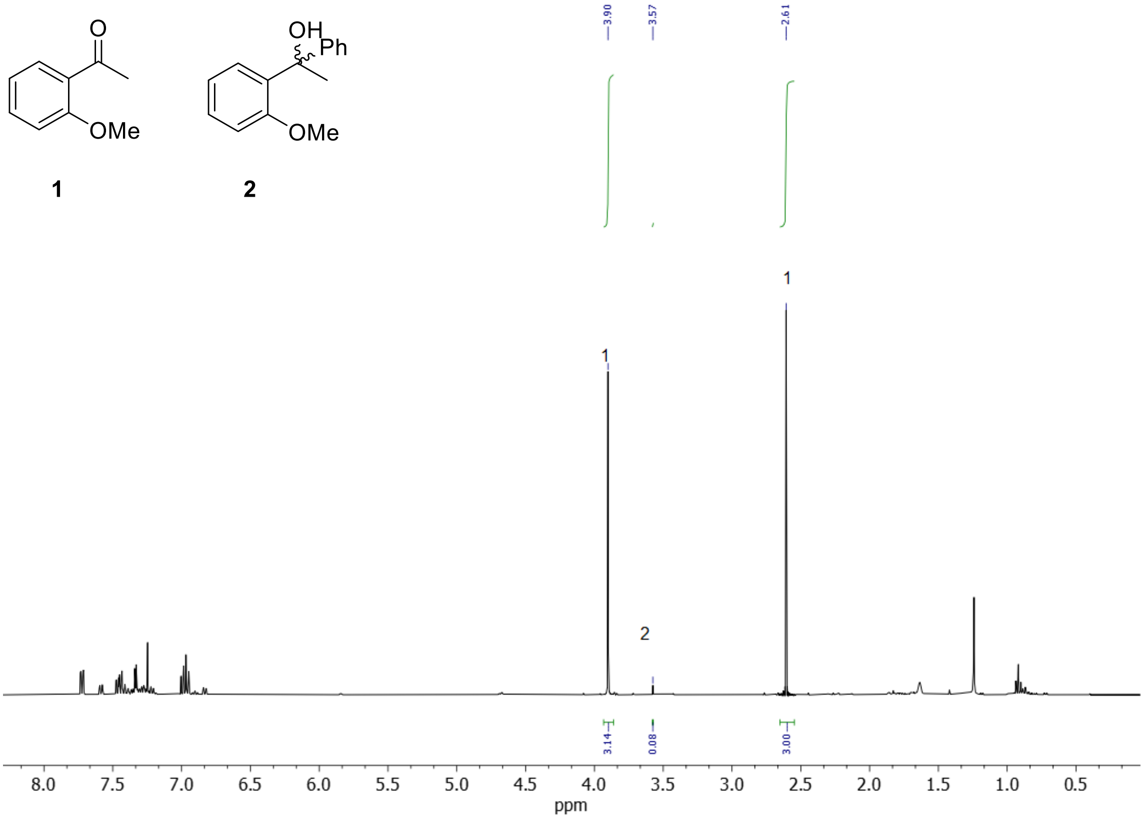

Figure S10. ^{1}H NMR spectrum of the crude product obtained after exposure of $PhLi_{gel}$ to air for 19 h followed by the reaction with 2′-methoxyacetophenone **1** ($CDCl_3$, 298 K, 400 MHz) – Fig. 2A, Entry 4 (Table S1).

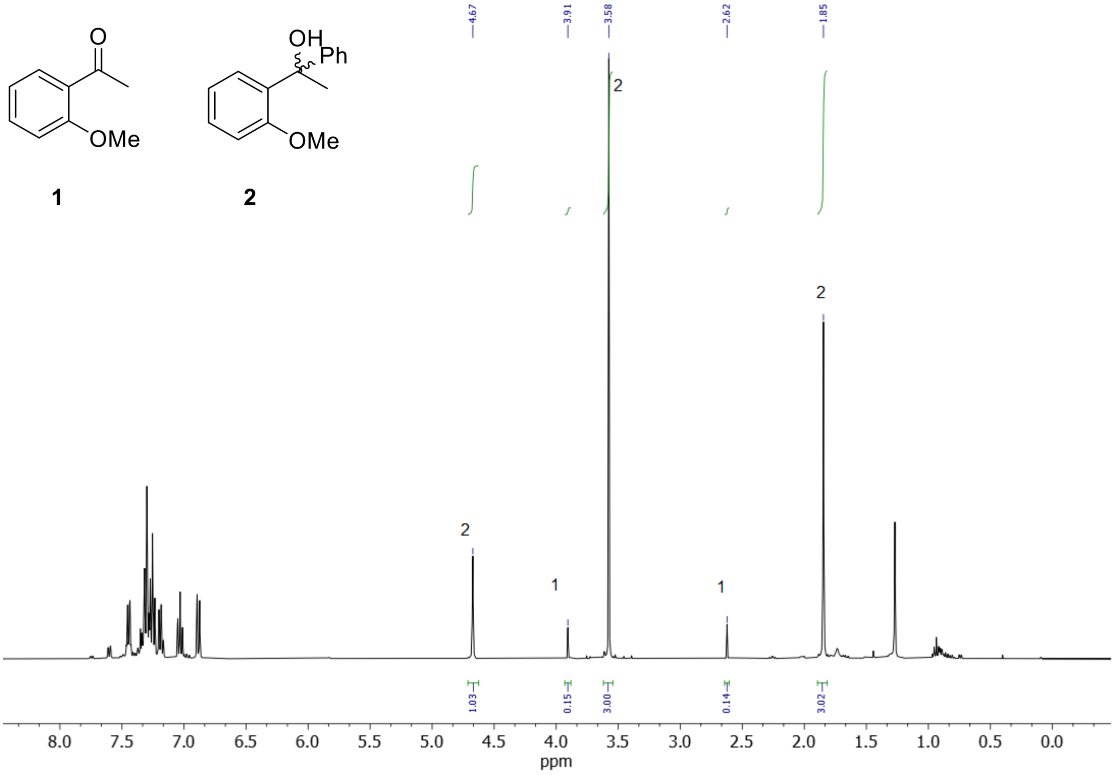

Figure S11. ^{1}H NMR spectrum of the crude product obtained after exposure of $PhLi_{gel}$ to air for 30 min and then stored in a closed vial for 19 h followed by the reaction with 2′-methoxyacetophenone **1** ($CDCl_3$, 298 K, 400 MHz) – Fig. 2A, Entry 5 (Table S1).

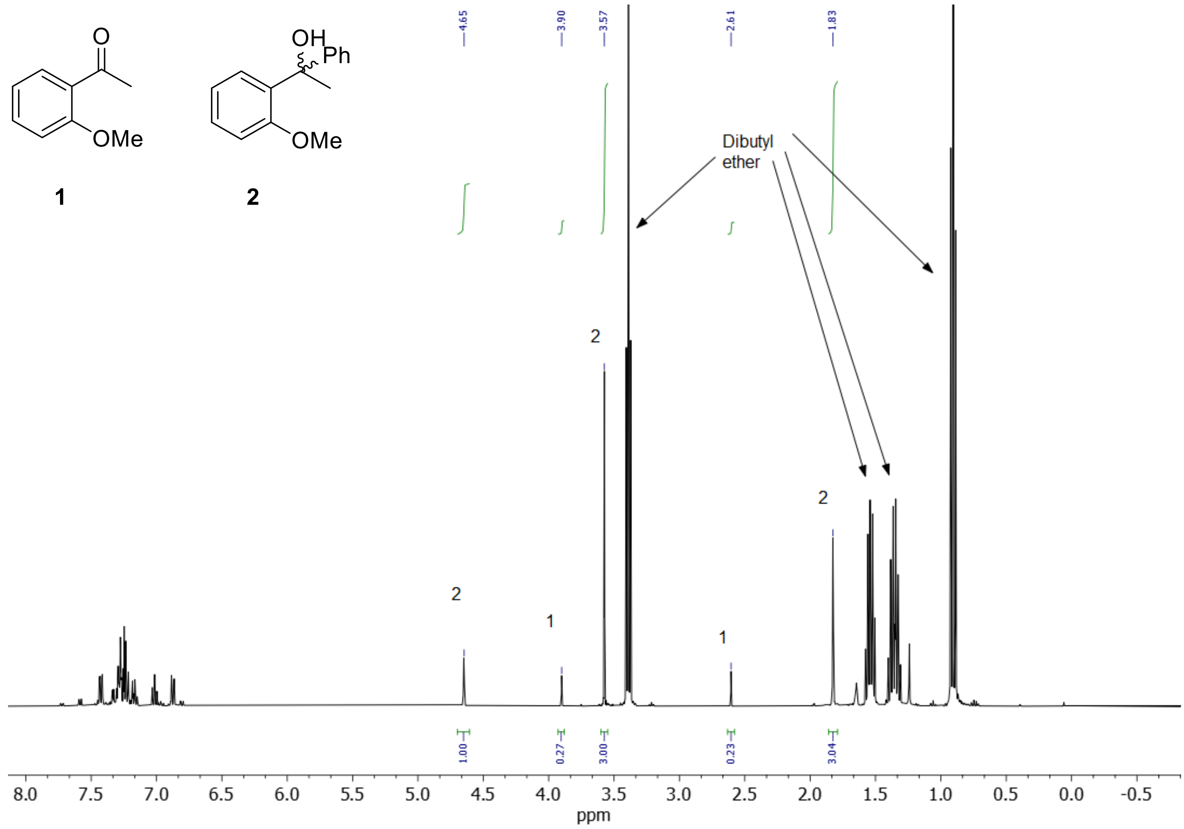

Figure S12. ^{1}H NMR spectrum of the crude product obtained after exposure of $PhLi_{gel}$ to air for 30 min and then stored in a closed vial for 25 days followed by the reaction with 2′-methoxyacetophenone **1** ($CDCl_3$, 298 K, 400 MHz) – Fig. 2A, Entry 6 (Table S1).

General procedure C: Reaction of *n*-BuLi$_{gel}$ with 2′-methoxyacetophenone 1 using an organolithium gel in a vial

n-BuLi$_{gel}$ (1.6 mmol) was prepared in a vial according to general procedure A. The organolithium gel was exposed to air by removing the rubber septum. After the specified time (see Table S2), 2′-methoxyacetophenone **1** (110.4 µL, 0.8 mmol) was added on the top of the gel at room temperature and under air. The mixture was vigorously stirred for 5 s before the reaction was quenched by the addition of water (0.5 mL). The solids in the reaction mixture were removed by filtration using a glass funnel and filter paper. This procedure removed most of the $C_{36}H_{74}$ gelator. The reaction vial and filter paper with the gelator were washed with additional dibutyl ether (3 x 2 mL) and the combined filtrates were dried ($MgSO_4$) and evaporated under reduced pressure to give the crude product. The crude product was analysed by ^{1}H NMR spectroscopy to determine the conversion based on relative integrals of the CH_3 group in the product and starting material. Compound **3** has been previously reported and the spectroscopic data were in agreement.[2]

Table S2 Screening of *n*-BuLi$_{gel}$ stability under ambient conditions (Fig. 2B).

Entry	Exposure to Air Time	Conversion to **3**[a]
1	30 min	70%
2	120 min[b]	77%
3	25 days[b]	77%

[a] Conversions determined by ^{1}H NMR spectroscopy using the relative integrals of key signals in product and starting material. [b] The vial was closed with a lid after 5 min exposure to air.

Reaction of *n*-BuLi$_{gel}$ with 2′-methoxyacetophenone 1 using *n*-BuLi$_{gel}$ in a vial: ^{1}H NMR Spectra

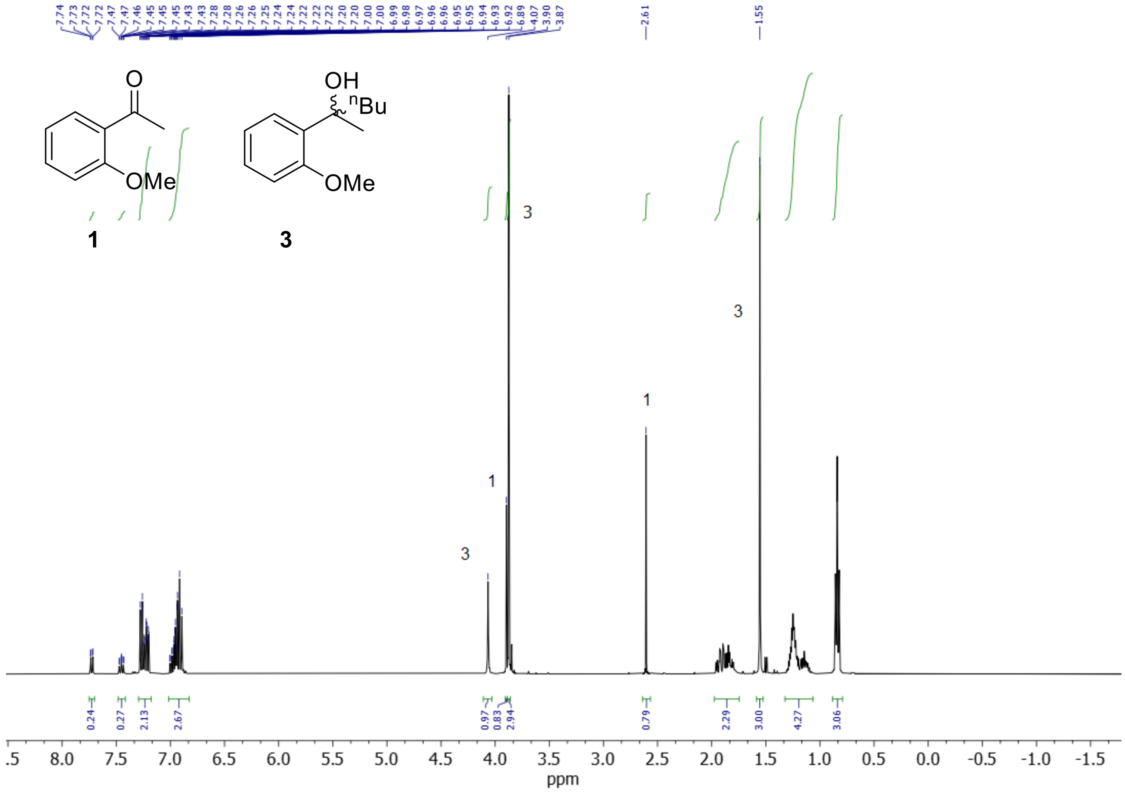

Figure S13. ^{1}H NMR spectrum of the crude product obtained after addition of *n*-BuLi solution to 2′-methoxyacetophenone **1** ($CDCl_3$, 298 K, 400 MHz) – see text in paper.

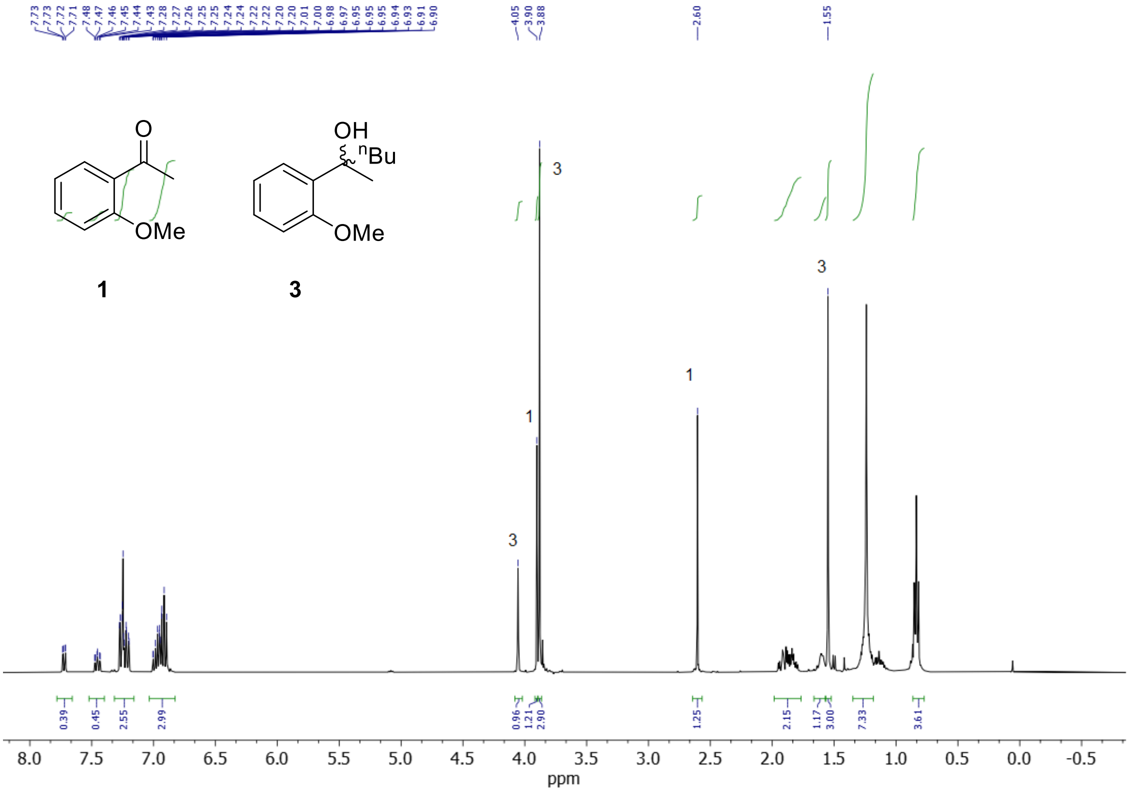

Figure S14. ^{1}H NMR spectrum of the crude product obtained after exposure of *n*-BuLi$_{gel}$ to air for 30 min followed by the reaction with 2′-methoxyacetophenone **1** ($CDCl_3$, 298 K, 400 MHz) – Fig. 2B, Entry 1 (Table S2).

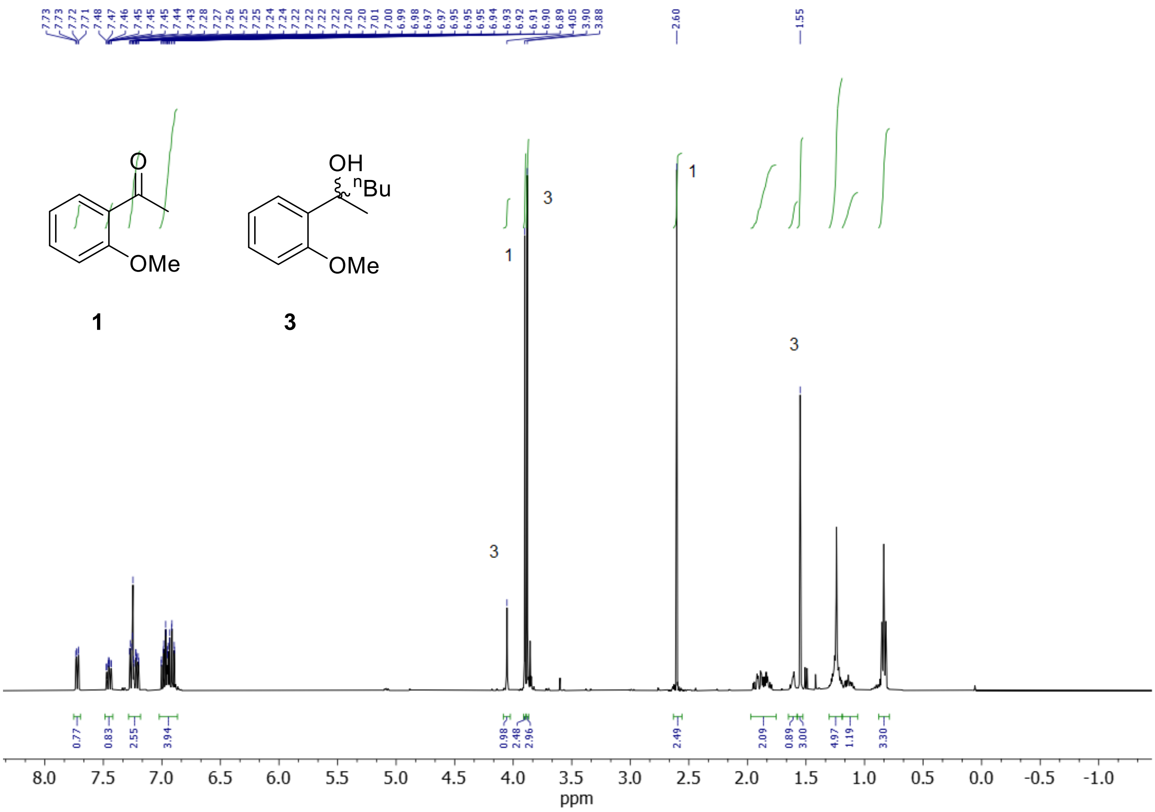

Figure S15. ^{1}H NMR spectrum of the crude product obtained after exposure of *n*-BuLi$_{gel}$ to air for 120 min followed by the reaction with 2′-methoxyacetophenone **1** ($CDCl_3$, 298 K, 400 MHz). 54% conversion to **3** (gel partly dried out).

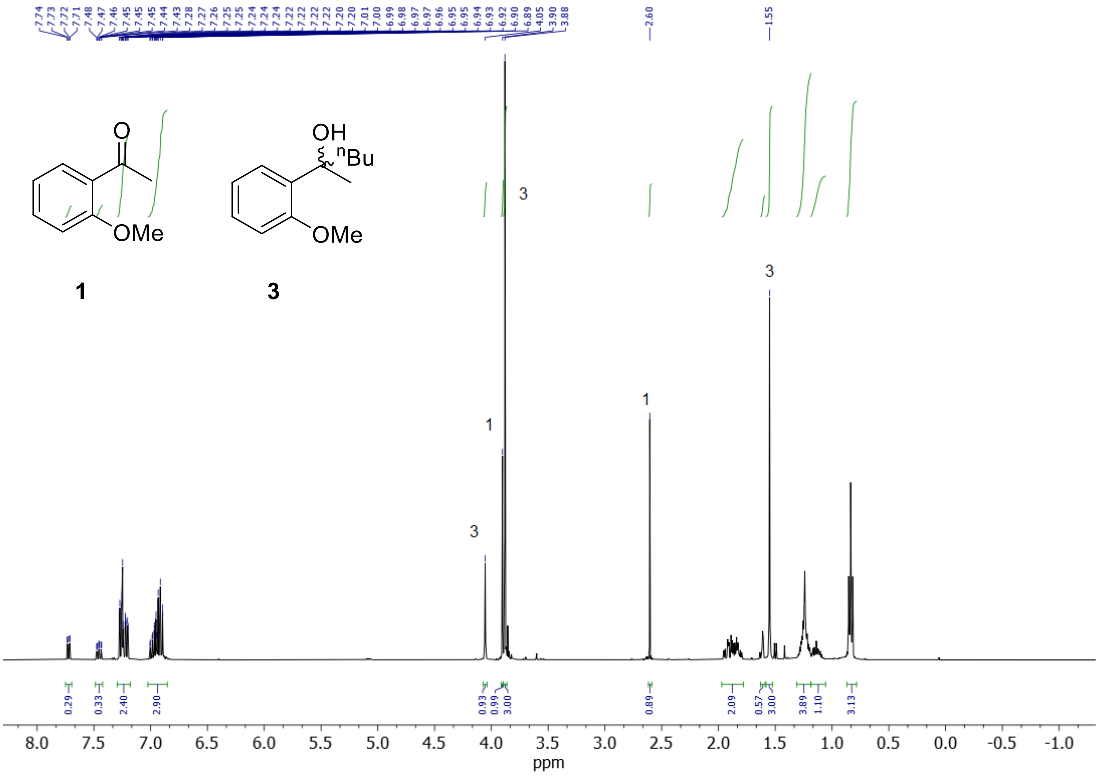

Figure S16. ^{1}H NMR spectrum of the crude product obtained after exposure of *n*-BuLi$_{gel}$ gel to air for 5 min and then stored in a closed vial for 120 min followed by the reaction with 2′-methoxyacetophenone **1** ($CDCl_3$, 298 K, 400 MHz). Fig. 2B, Entry 2 (Table S2).

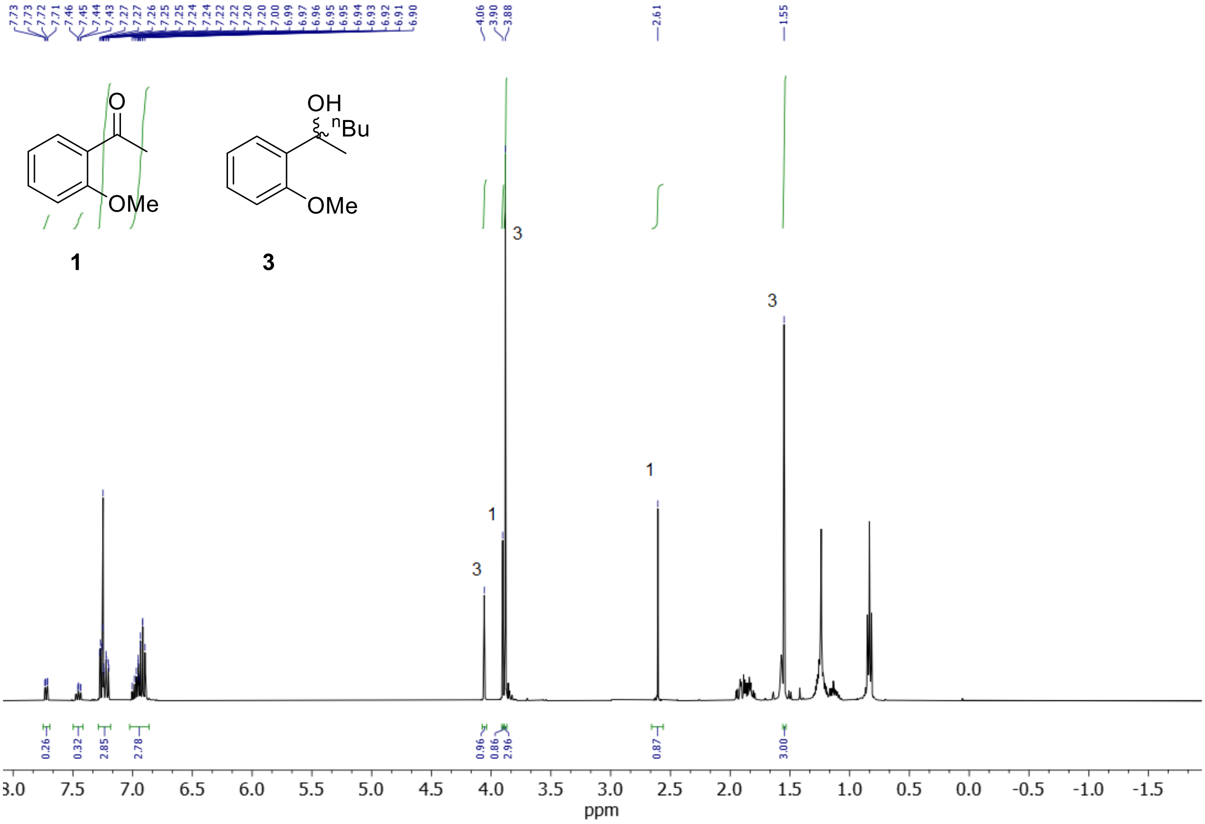

Figure S17. ^{1}H NMR spectrum of the crude product obtained after exposure of B *n*-uLi$_{gel}$ gel to air for 5 min and then stored in a closed vial for 25 days followed by the reaction with 2′-methoxyacetophenone **1** ($CDCl_3$, 298 K, 400 MHz). Fig. 2B, Entry 3 (Table S2).

General procedure D: Reaction of $PhLi_{gel}$ with benzophenone 4 using an organolithium gel in a vial

$PhLi_{gel}$ (1.6 mmol) was prepared in a vial according to general procedure A. The organolithium gel was exposed to air by removing the rubber septum. After the specified time (see Table S3), a solution of benzophenone **4** (0.151 g, 0.8 mmol) in dry dibutyl ether (1 mL) was added on the top of the gel at room temperature and under air. The mixture was vigorously stirred for 5 s before the reaction was quenched by the addition of water (0.5 mL). The solids in the reaction mixture were removed by filtration using a glass funnel and filter paper. This procedure removed most of the $C_{36}H_{74}$ gelator. The reaction vial and filter paper with the gelator were washed with additional dibutyl ether (3 x 2 mL) and the combined filtrates were dried ($MgSO_4$) and evaporated under reduced pressure to give the crude product. The crude product was analysed by ^1H NMR spectroscopy to determine the conversion based on relative integrals of key signals in the product and starting material. Compound **5** has been previously reported and the spectroscopic data were in agreement.[2]

Table S3 Reaction of $PhLi_{gel}$ with benzophenone **4** under ambient conditions (Fig. 2C).

Entry	Exposure to Air Time	Conversion to **5**[a]
1	30 min	98%
2	120 min	98%
3	18 h[b]	96%

[a] Conversions determined by ^1H NMR spectroscopy using the relative integrals of key signals in product and starting material. [b] The vial was closed with a lid after 30 min exposure to air.

Reaction of $PhLi_{gel}$ with benzophenone 4 using $PhLi_{gel}$ in a vial: ^1H NMR Spectra

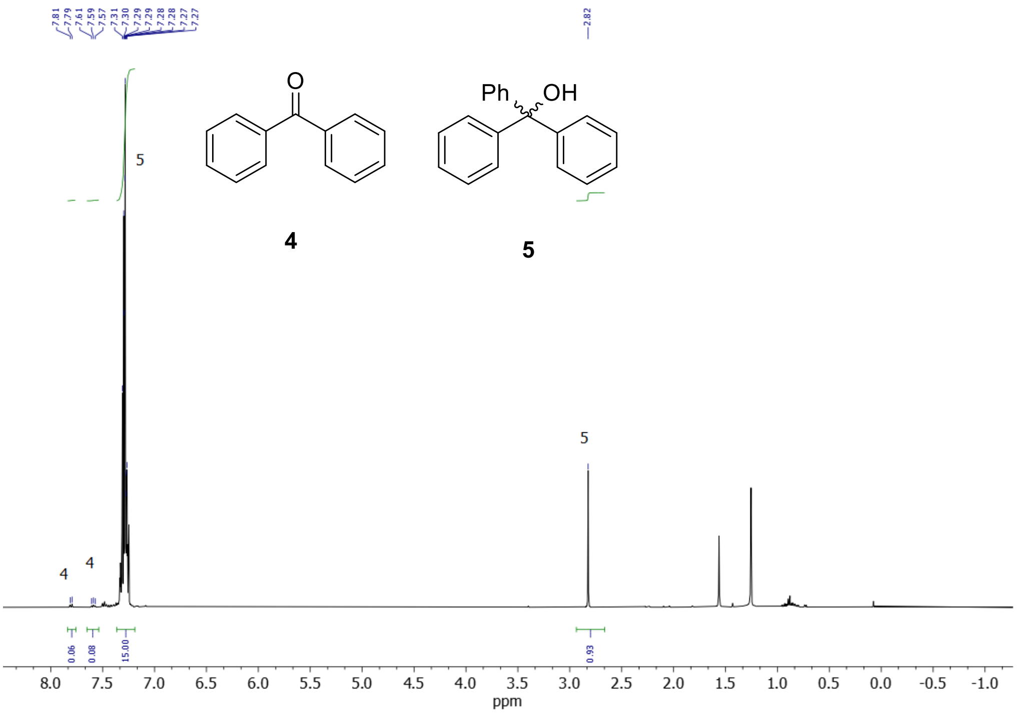

Figure S18. ^1H NMR spectrum of the crude product obtained after exposure of $PhLi_{gel}$ to air for 30 min followed by the reaction with benzophenone **4** ($CDCl_3$, 298 K, 400 MHz). 98% Conversion to **5** (Fig. 2C, Table S3, Entry 1).

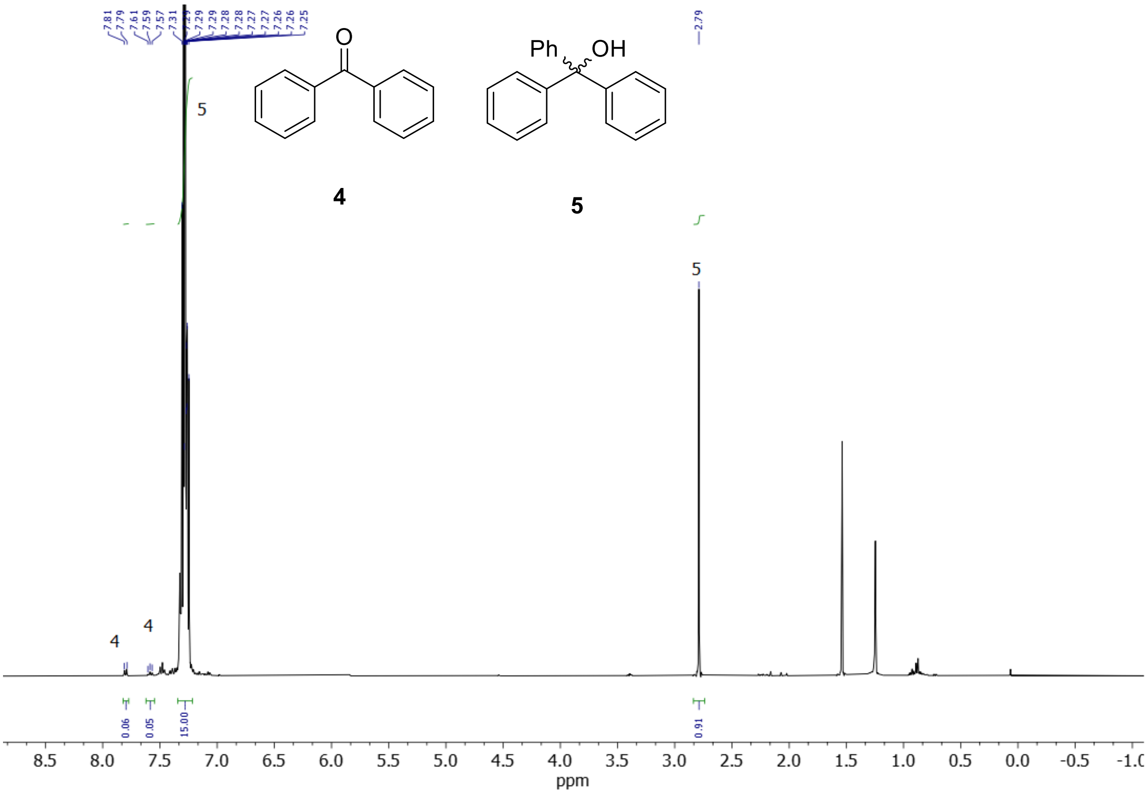

Figure S19. ^{1}H NMR spectrum of the crude product obtained after exposure of $PhLi_{gel}$ to air for 120 min followed by reaction with benzophenone **4** ($CDCl_3$, 298 K, 400 MHz). 98% Conversion to **5** (Table S3, Entry 2).

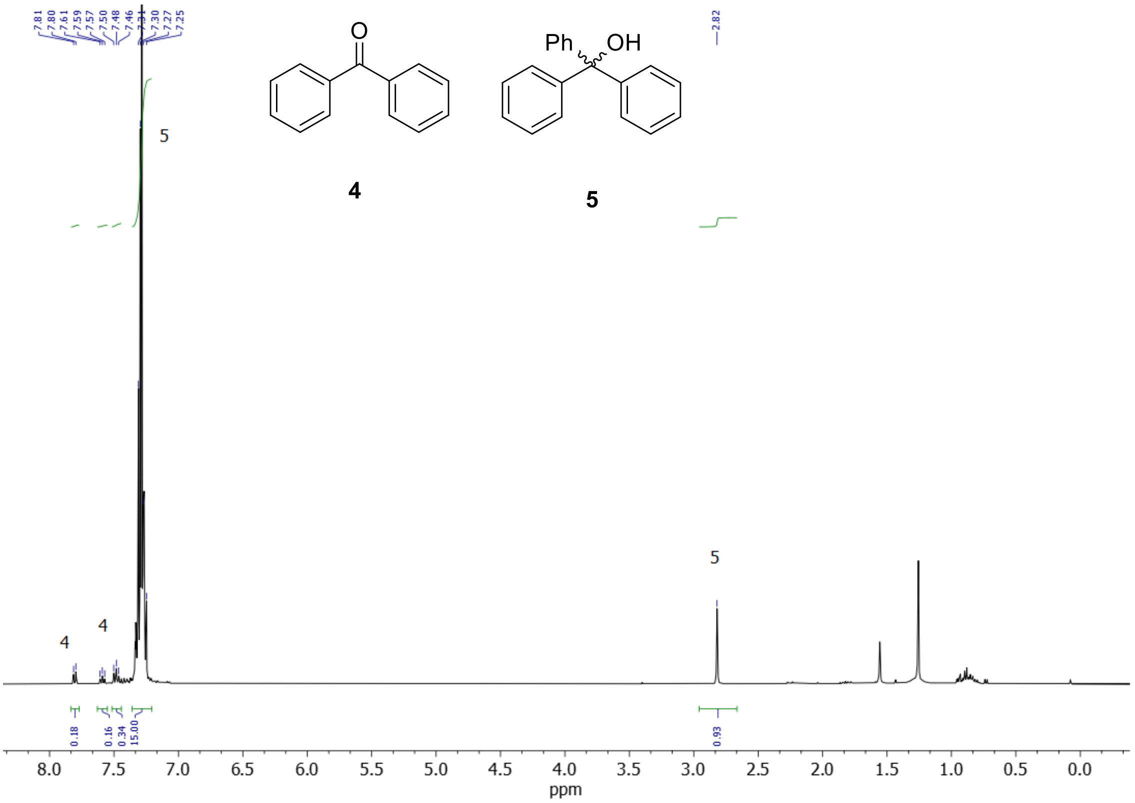

Figure S20. ^{1}H NMR spectrum of the crude product obtained after exposure of $PhLi_{gel}$ to air for 30 min and then stored in a closed vial for 18 h followed by the reaction with benzophenone **4** ($CDCl_3$, 298 K, 400 MHz). 96% Conversion to **5** (Table S3, Entry 3).

General procedure E: Reaction of *n*-BuLi$_{gel}$ with benzophenone 4 using an organolithium gel in a vial

n-BuLi$_{gel}$ (1.6 mmol) was prepared in a vial according to general procedure A. The organolithium gel was exposed to air by removing the rubber septum. After the specified time (see Table S4), a solution of benzophenone **4** (0.151 g, 0.8 mmol) in dry hexane (1 mL) was added on the top of the gel at room temperature and under air. The mixture was vigorously stirred for 5 s before the reaction was quenched by the addition of water (0.5 mL). The solids in the reaction mixture were removed by filtration using a glass funnel and filter paper. This procedure removed most of the $C_{36}H_{74}$ gelator. The reaction vial and filter paper with the gelator were washed with additional dibutyl ether (3 x 2 mL) and the combined filtrates were dried ($MgSO_4$) and evaporated under reduced pressure to give the crude product. The crude product was analysed by ^{1}H NMR spectroscopy to determine the conversion based on relative integrals of key signals in the product and starting material. Compounds **8** and **S1** has been previously reported and the spectroscopic data were in agreement.[2]

Table S4 Reaction of *n*-BuLi$_{gel}$ with benzophenone **4** under ambient conditions (Fig. 2C).

Entry	Exposure to Air Time	Benzophenone **4**	Conversion to **8**[a]	Conversion to **S1**[a]
1	5 min	3%	74%	23%
2	30 min	3%	72%	25%
3	120 min[b]	2%	71%	27%

[a] Conversions determined by ^{1}H NMR spectroscopy using the relative integrals of key signals in product and starting material. [b] The vial was closed with a lid after 5 min exposure to air.

Reaction of *n*-BuLi$_{gel}$ with benzophenone 4 using *n*-BuLi$_{gel}$ in a vial: ^{1}H NMR Spectra

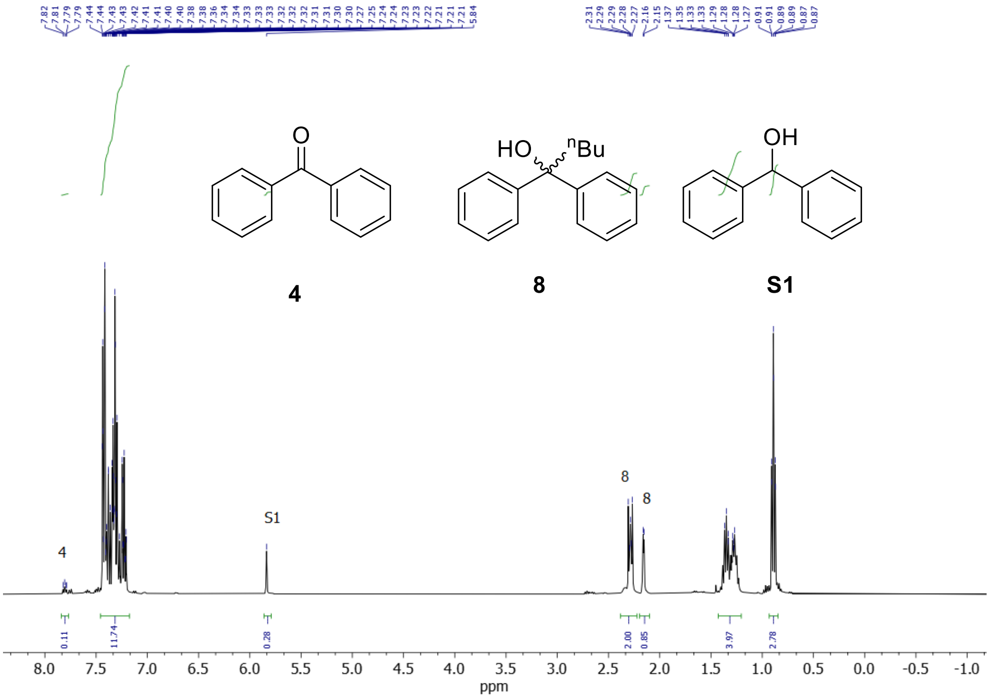

Figure S21. ^{1}H NMR spectrum of the crude product obtained after addition of *n*-BuLi solution to benzophenone **4** ($CDCl_3$, 298 K, 400 MHz). 2% **4**; 74% **8**; 24% **S1**.

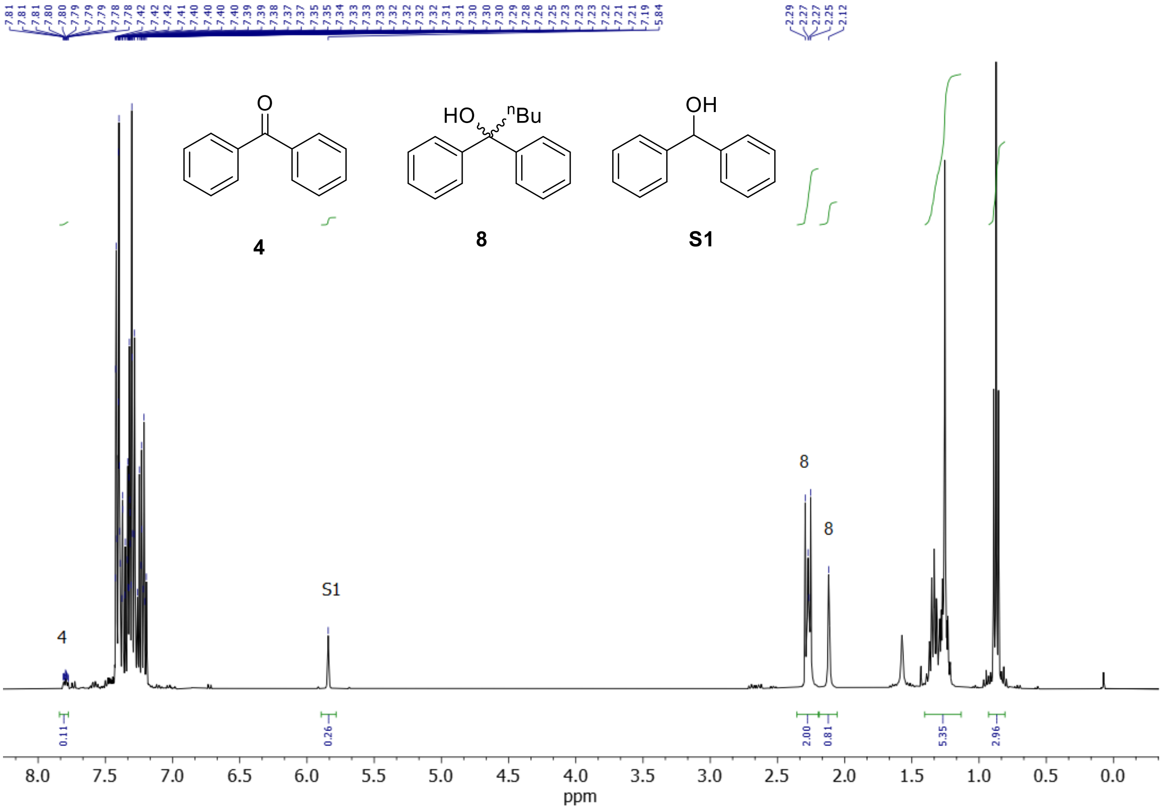

Figure S22. ^1H NMR spectrum of the crude product obtained after exposure of *n*-$BuLi_{gel}$ gel to air for 5 min followed by the reaction with benzophenone **4** ($CDCl_3$, 298 K, 400 MHz) –Table S4, Entry 1.

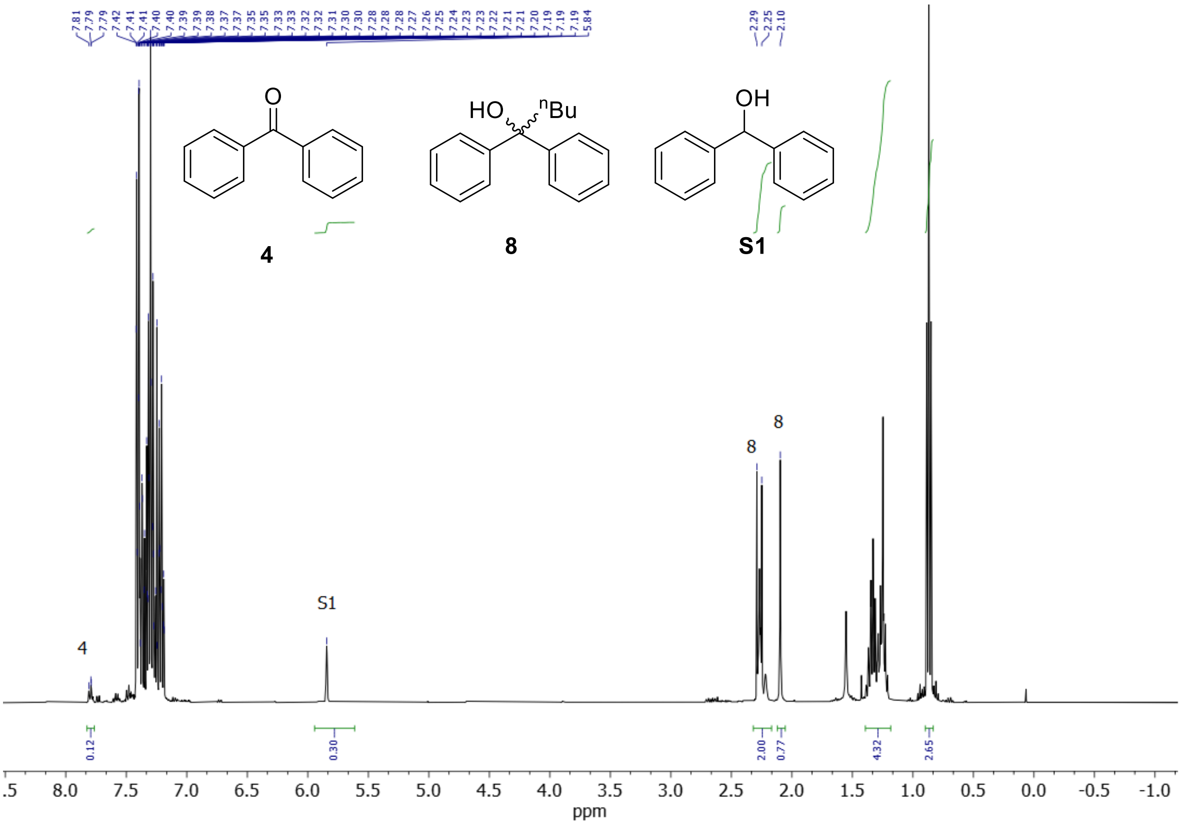

Figure S23. ^1H NMR spectrum of the crude product obtained after exposure of *n*-$BuLi_{gel}$ gel to air for 30 min followed by the reaction with benzophenone **4** ($CDCl_3$, 298 K, 400 MHz) – Fig. 2C, Table S4, Entry 2.

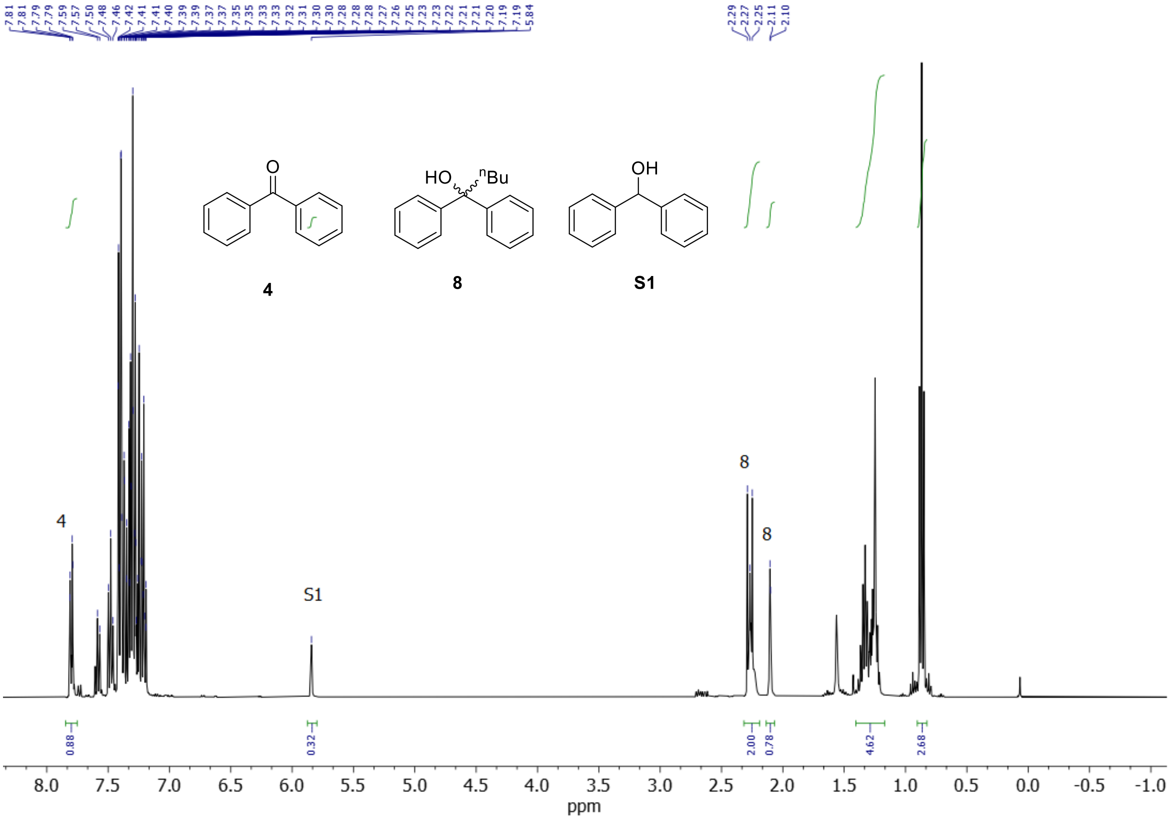

Figure S24. ^{1}H NMR spectrum of the crude product obtained after exposure of *n*-BuLi$_{gel}$ to air for 120 min followed by the reaction with benzophenone **4** ($CDCl_3$, 298 K, 400 MHz). 16% **4**; 60% **8**; 24% **S1** (gel partly dried out).

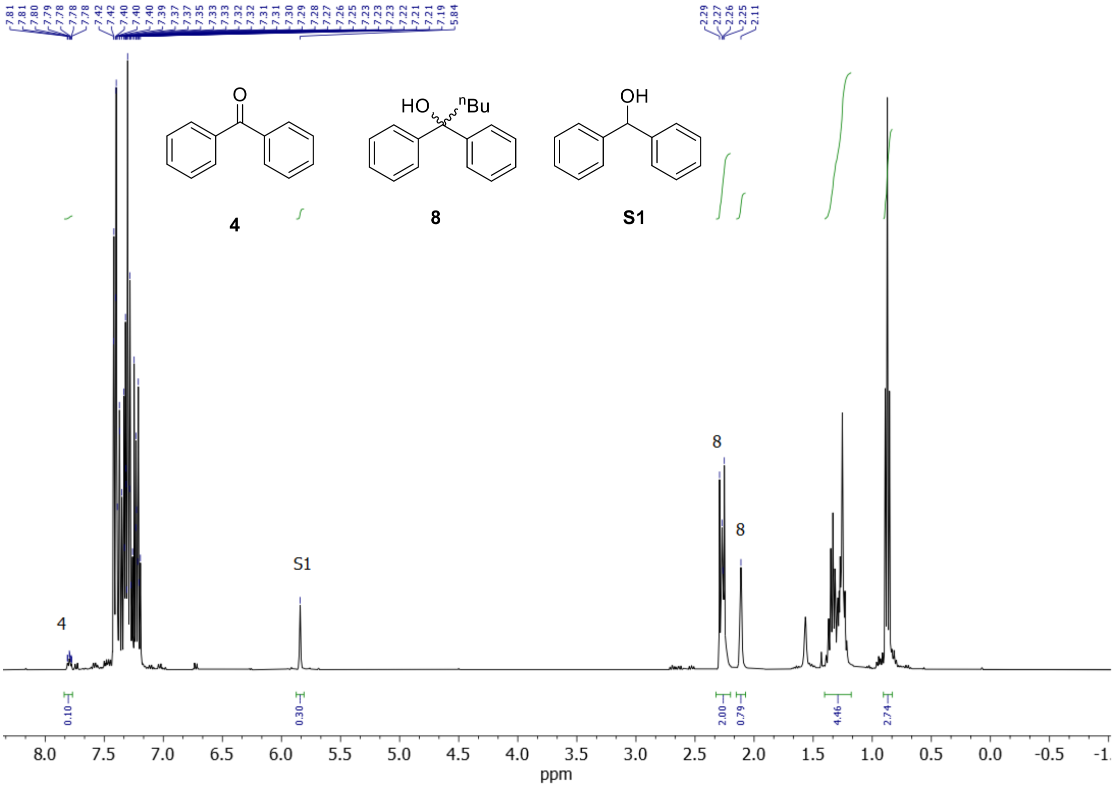

Figure S25. ^{1}H NMR spectrum of the crude product obtained after exposure of *n*-BuLi$_{gel}$ to air for 5 min and then stored in a closed vial for 120 min followed by the reaction with benzophenone **4** ($CDCl_3$, 298 K, 400 MHz) – Table S4, Entry 3.

Reaction of $PhLi_{gel}$ with *N*-benzylideneaniline 6 using an organolithium gel in a vial

$PhLi_{gel}$ (1.6 mmol) was prepared in a vial according to general procedure A. The organolithium gel was exposed to air by removing the rubber septum. After 30 min, a solution of *N*-benzylideneaniline **6** (0.145 g, 0.8 mmol) in dry dibutyl ether (1 mL) was added on the top of the gel at room temperature and under air. The mixture was vigorously stirred for 5 s before the reaction was quenched by the addition of water (0.5 mL). The solids in the reaction mixture were removed by filtration using a glass funnel and filter paper. This procedure removed most of the $C_{36}H_{74}$ gelator. The reaction vial and filter paper with the gelator were washed with additional dibutyl ether (3 x 2 mL) and the combined filtrates were dried ($MgSO_4$) and evaporated under reduced pressure to give the crude product. The crude product was analysed by ^1H NMR spectroscopy to determine the conversion based on relative integrals of key signals in the product and starting material. Compound **7** has been previously reported and the spectroscopic data were in agreement.[3]

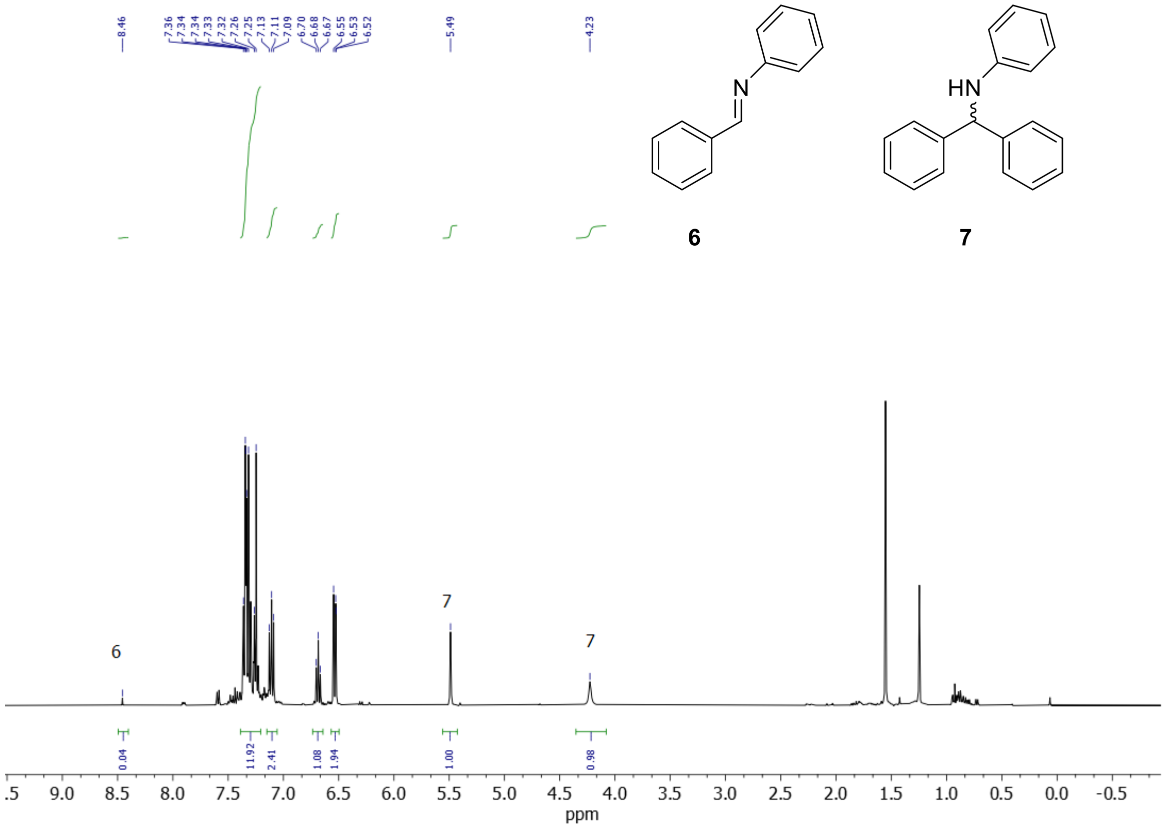

Figure S26. ^1H NMR spectrum of the crude product obtained after exposure of $PhLi_{gel}$ to air for 30 min followed by the reaction with *N*-benzylideneaniline **6** ($CDCl_3$, 298 K, 400 MHz). 96% Conversion to **7** (Fig. 2C).

Reaction of *n*-BuLi$_{gel}$ with *N*-benzylideneaniline 6 using an organolithium gel in a vial

n-BuLi$_{gel}$ (1.6 mmol) was prepared in a vial according to general procedure A. The organolithium gel was exposed to air by removing the rubber septum. After 30 min, a solution of *N*-benzylideneaniline **6** (0.145 g, 0.8 mmol) in dry hexane (1 mL) was added on the top of the gel at room temperature and under air. The mixture was vigorously stirred for 5 s before the reaction was quenched by the addition of water (0.5 mL). The solids in the reaction mixture were removed by filtration using a glass funnel and filter paper. This procedure removed most of the $C_{36}H_{74}$ gelator. The reaction vial and filter paper with the gelator were washed with additional dibutyl ether (3 x 2 mL) and the combined filtrates were dried ($MgSO_4$) and evaporated under reduced pressure to give the crude product. The crude product was analysed by ^{1}H NMR spectroscopy to determine the conversion based on relative integrals of key signals in the product and starting material. Compound **9** has been previously reported and the spectroscopic data were in agreement.[3]

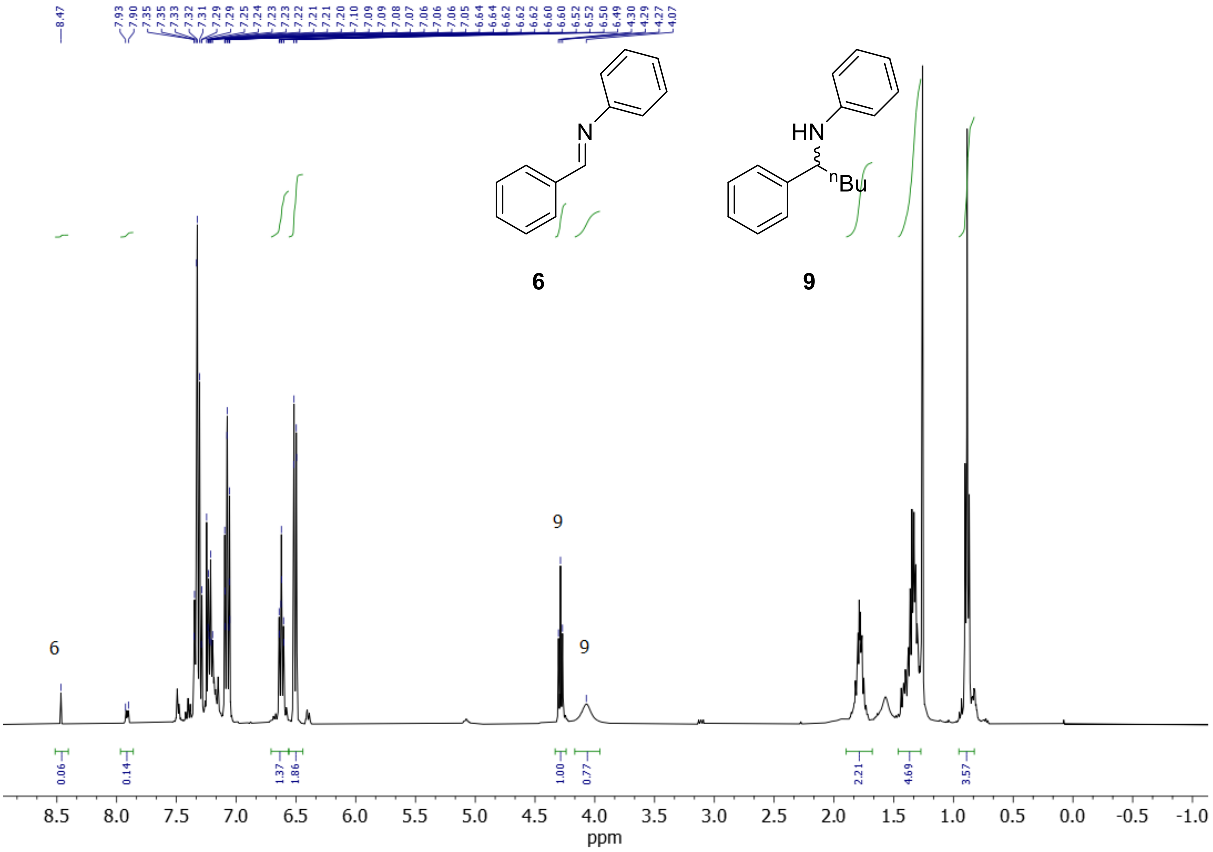

Figure S27. ^{1}H NMR spectrum of the crude product obtained after exposure of *n*-BuLi$_{gel}$ to air for 30 min followed by the reaction with *N*-benzylideneaniline **6** ($CDCl_3$, 298 K, 400 MHz). 94% Conversion to **9** (Fig. 2C).

4. Preparation of $PhLi_{gel}$ and *n*-$BuLi_{gel}$ Blocks and Subsequent Reactions (Results in Fig. 3)

General procedure F: preparation of robust organolithium gels ($PhLi_{gel}$ and *n*-$BuLi_{gel}$ blocks)

A 5 mL vial was dried in the oven and allowed to cool under a nitrogen atmosphere. The vial was charged with the gelator $C_{36}H_{74}$ (250.0 mg, 0.49 mmol, 16.7% wt/vol in the case of PhLi and 15.6% wt/vol in the case of *n*-BuLi), sealed with a rubber septum and flushed with nitrogen three times. Anhydrous and degassed solvent (1 mL of dibutyl ether in the case of PhLi or 1 mL of hexane in the case of *n*-BuLi) was added through the septum followed by the addition of the organolithium reagent (0.50 mL of PhLi – 1.91 M in dibutyl ether or 0.6 mL of *n*-BuLi – 1.6 M in hexane). The vial (kept under a nitrogen atmosphere using a balloon) was carefully heated until all of the gelator had dissolved. The hot hydrosol was quickly transferred under a nitrogen atmosphere via a needle into a 2 mL syringe (previously flushed with nitrogen and pre-heated in the oven). The syringe was immediately placed in iced water for 1 min until the organogel formed. The organolithium gel was kept in the syringe under a nitrogen atmosphere prior to use. In order to use the organolithium gel, the upper part of the syringe was carefully cut with scissors and the gel block was removed. This is shown in Fig. S28.

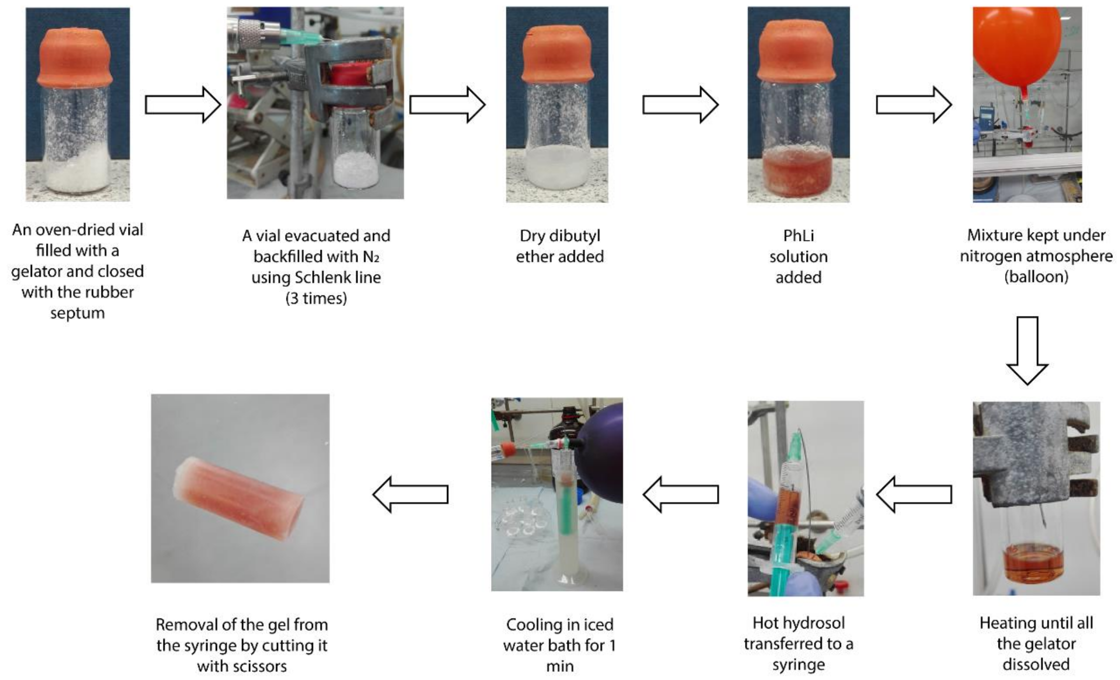

Figure S28. Schematic illustration of the preparation of the $PhLi_{gel}$ block.

It was necessary to vary the concentration of the gelator in order to obtain a stable gel block. The results obtained using PhLi are shown in Table S5.

It was found that a 16.7% wt/vol of gelator was necessary to obtain a robust gel.

Table S5. Screening of the stability of PhLi organogels (see Fig. S29)

Entry	Amount of PhLi (1.9 M) [mL]	Amount of additional dibutyl ether [mL]	Final concentration of PhLi in the gel [M]	Concentration of $C_{36}H_{74}$ gelator (% wt/vol)	Stable gel [Yes/No]
1	1	0	1.9	5%	No
2	1	0	1.9	10%	No
3	1	0	1.9	15%	No
4	1	0	1.9	20%	No
5	1	0	1.9	25%	No
6	1	2	0.6	3.3%	No
7	1	2	0.6	5%	No
8	1	2	0.6	8.3%	No
9	1	2	0.6	10%	No
10	0.5	1	0.6	16.7%	Yes

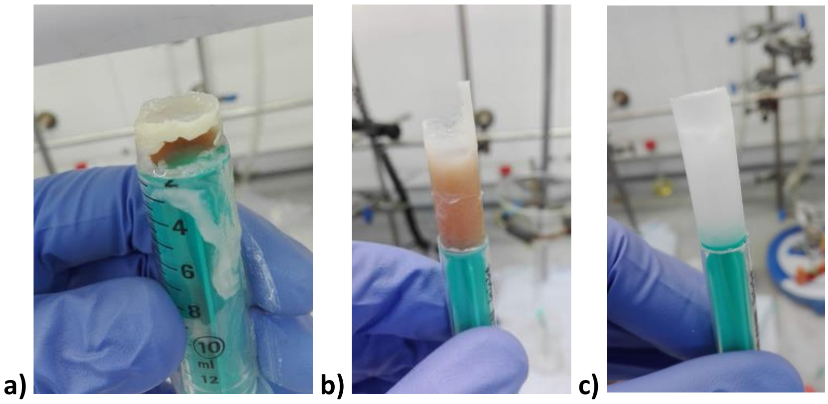

Figure S29. a) Unstable PhLi gel after exposure to air (Table S5, Entries 1-9). b) Robust $PhLi_{gel}$ block (Table S5, Entry 10) after exposure to air. c) Robust *n*-$BuLi_{gel}$ block (15.6% wt/vol of gelator).

Long-term stability studies for $PhLi_{gel}$ (16.7% wt/vol) and *n*-$BuLi_{gel}$ (15.6% wt/vol) using titrations

$PhLi_{gel}$ (16.7% wt/vol)

$PhLi_{gel}$ (0.95 mmol) was prepared in a 7 mL vial according to general procedure A using gelator $C_{36}H_{74}$ (250.0 mg, 0.5 mmol, 16.7% wt/vol), anhydrous and degassed dibutyl ether (2 mL) and PhLi (0.5 mL of PhLi – 1.9 M in dibutyl ether, 1.6 mmol). The organolithium gel was exposed to air for <10 s by removing the rubber septum and replacing it with a screw cap. It was sealed with electrical tape and stored under nitrogen in a desiccator for the time specified in Table S6.

The amount of PhLi in the gel was determined by titration experiments using (+)-menthol (1.0 $molL^{-1}$ in THF) with 2,2'-bipyidine as an indicator.[4]

Table S6. Concentration of PhLi inside the gel determined by titration.

Entry	Days	Concentration ($molL^{-1}$)
1	0	0.61
2	1	0.59
3	7	0.60
4	14	0.61
5	21	0.62
6	28	0.57
7	35	0.61
8	42	0.60

n-$BuLi_{gel}$ (15.6% wt/vol)

n-$BuLi_{gel}$ (0.96 mmol) was prepared in a 7 mL vial according to general procedure A using gelator $C_{36}H_{74}$ (250.0 mg, 0.5 mmol, 15.6% wt/vol), anhydrous and degassed hexane (1 mL) and *n*-BuLi (0.6 mL of *n*-BuLi – 1.6 M in hexane, 0.96 mmol). The organolithium gel was exposed to air for <10 s by removing the rubber septum and replacing it with a screw cap. It was sealed with electrical tape and stored under nitrogen in a desiccator for the time specified in Table S7.

The amount of *n*-BuLi in the gel was determined by titration experiments using (+)-menthol (1.0 $molL^{-1}$ in THF) with 2,2'-bipyidine as an indicator.[4]

Table S7. Concentration of *n*-BuLi inside the gel determined by titration.

Entry	Days	Concentration ($molL^{-1}$)
1	0	0.63
2	1	0.62
3	7	0.63
4	14	0.62
5	21	0.57
6	28	0.64
7	36	0.58
8	42	0.57

General procedure G: Reaction of PhLi$_{gel}$ block with 2′-methoxyacetophenone 1 using an organolithium gel block

A PhLi$_{gel}$ block (0.95 mmol) was prepared according to general procedure F. After the specified time and storage conditions (see Table S8), the gel block was carefully placed in a 5 mL round-bottomed flask containing a solution of 2′-methoxyacetophenone **1** (65.4 µL, 0.475 mmol) in dry dibutyl ether (2 mL) at room temperature and under air. The mixture was vigorously stirred for 5 min before the reaction was quenched by the addition of water (0.5 mL). The solids in the reaction mixture were removed by filtration using a glass funnel and filter paper. This procedure removed most of the $C_{36}H_{74}$ gelator. The reaction vial and filter paper with the gelator were washed with additional dibutyl ether (3 x 2 mL) and the combined filtrates were dried ($MgSO_4$) and evaporated under reduced pressure to give the crude product. The crude product was analysed by ^{1}H NMR spectroscopy to determine the conversion based on relative integrals of the CH_3 group in the product and starting material. Compound **2** has been previously reported and the spectroscopic data were in agreement.[2]

Table S8. Stability of PhLi$_{gel}$ blocks under different conditions (Fig. 3B).

Entry	Storage conditions	Conversion to **2**[a]
1	0.5 min, air[b]	96%
2	30 min, air[b]	78%
3	2 hours, closed vial[c]	98%
4	3 days, vial, N_2, rubber septumd	93%
5	7 days, vial, N_2, rubber septum[d]	86%
6	37 days, vial, N_2, rubber septum[d]	24%
7	10 days, closed vial, N_2[c]	97%
8	32 days, closed vial, N_2[c]	61%

[a] Conversions determined by ^{1}H NMR spectroscopy using the relative integrals of key signals in product and starting material. [b] The PhLi$_{gel}$ block was prepared and exposed to air on a petri dish. [c] The PhLi$_{gel}$ block was transferred to a vial flushed with N_2 and closed with a lid. [d] The PhLi$_{gel}$ block was prepared in a vial and the vial was closed with a rubber septum.

a) b)

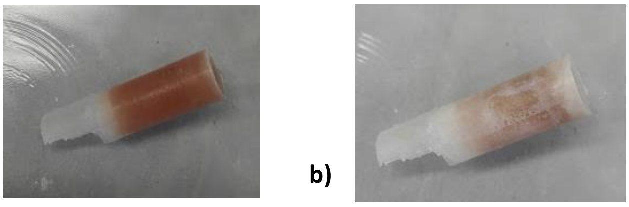

Figure S30. PhLi gel block: a) exposure to air, time = 0 min. b) exposure to air, time = 30 min (Table S8, entry 2).

Reaction of $PhLi_{gel}$ block with 2′-methoxyacetophenone 1: ^1H NMR Spectra

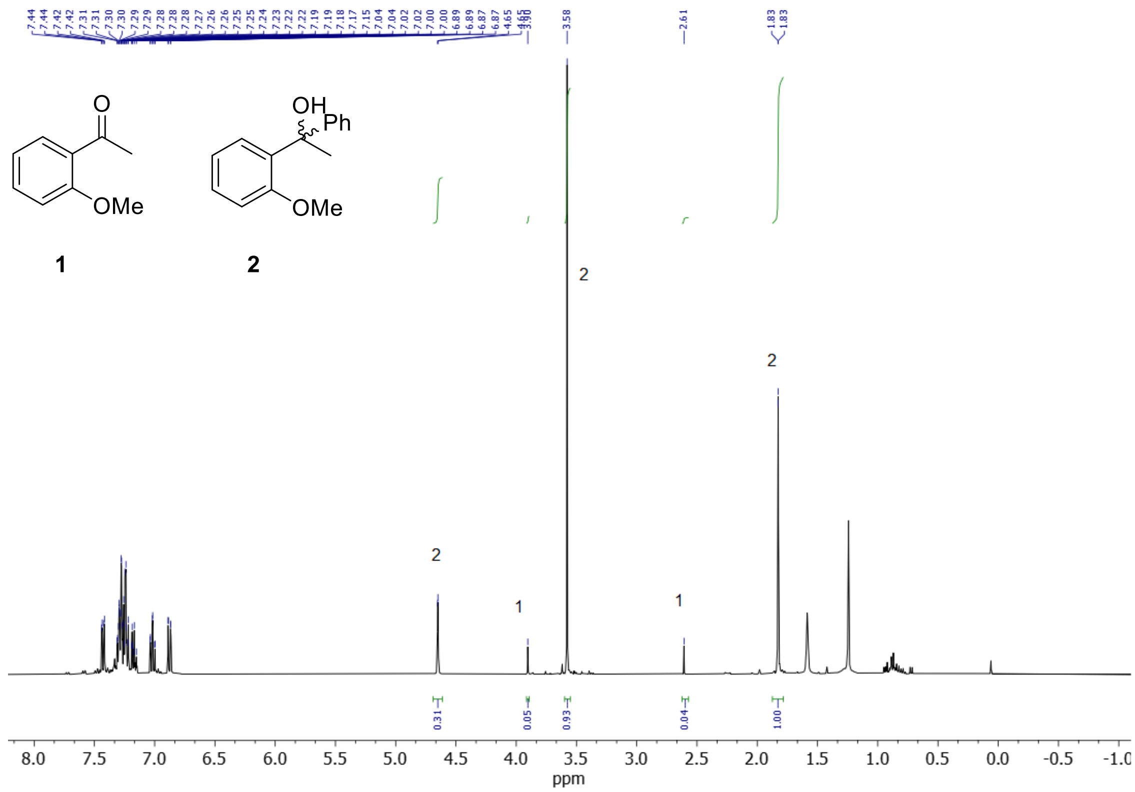

Figure S31. ^1H NMR spectrum of the crude product obtained after exposure of a $PhLi_{gel}$ block to air on a petri dish for 30 sec followed by the reaction with 2′-methoxyacetophenone **1** ($CDCl_3$, 298 K, 400 MHz) – Fig. 3B, Entry 1 (Table S8, Entry 1).

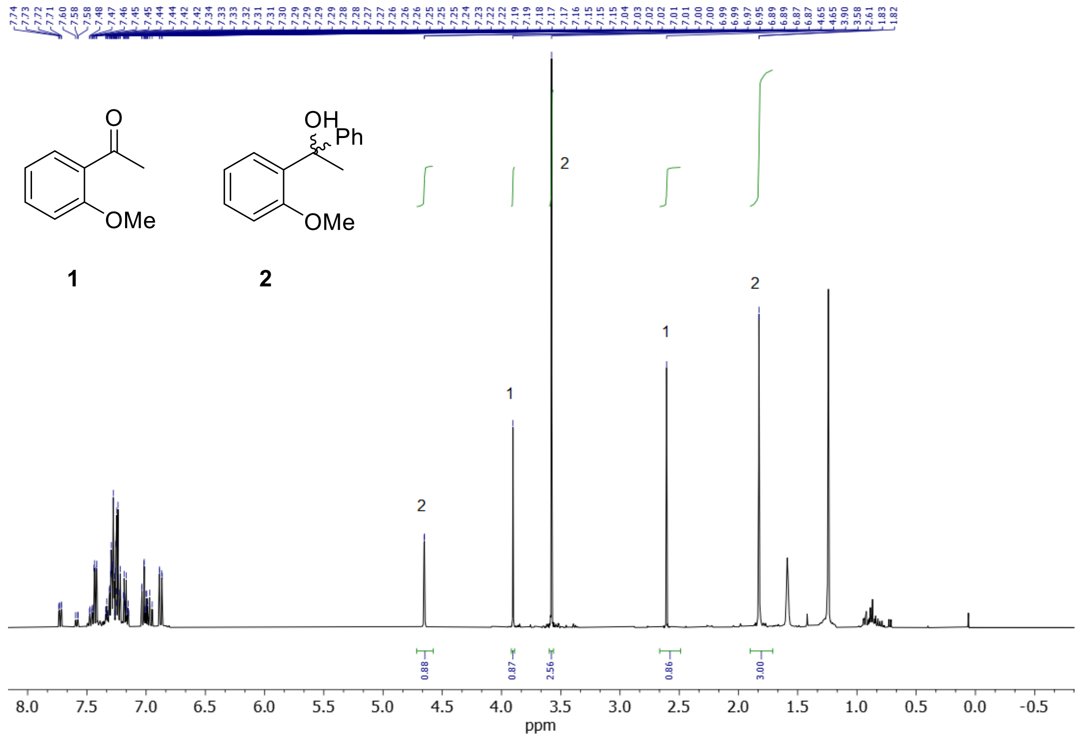

Figure S32. ^1H NMR spectrum of the crude product obtained after exposure of a $PhLi_{gel}$ block to air on a petri dish for 30 min followed by the reaction with 2′-methoxyacetophenone **1** ($CDCl_3$, 298 K, 400 MHz) – Fig. 3B, Entry 2 (Table S8, Entry 2).

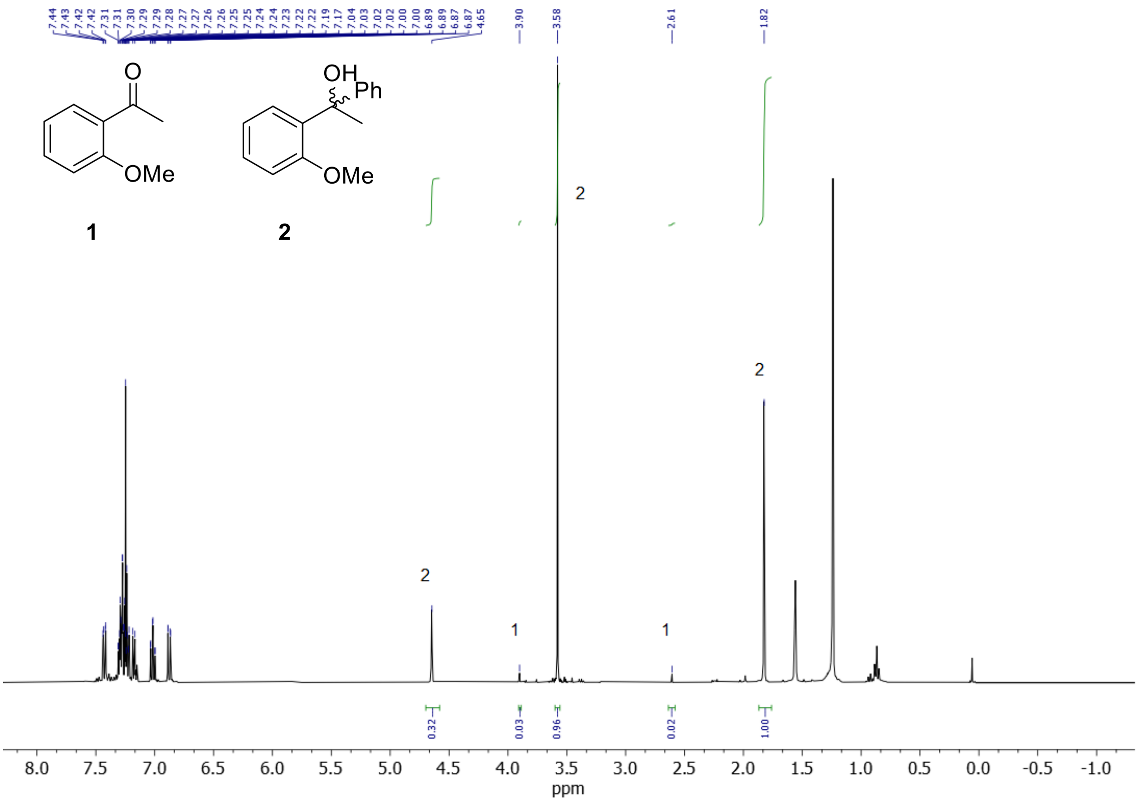

Figure S33. ^{1}H NMR spectrum of the crude product obtained using a $PhLi_{gel}$ block that was stored in a closed vial for 2 hours followed by the reaction with 2′-methoxyacetophenone **1** ($CDCl_3$, 298 K, 400 MHz) – – Fig. 3B, Entry 3 (Table S8, Entry 3).

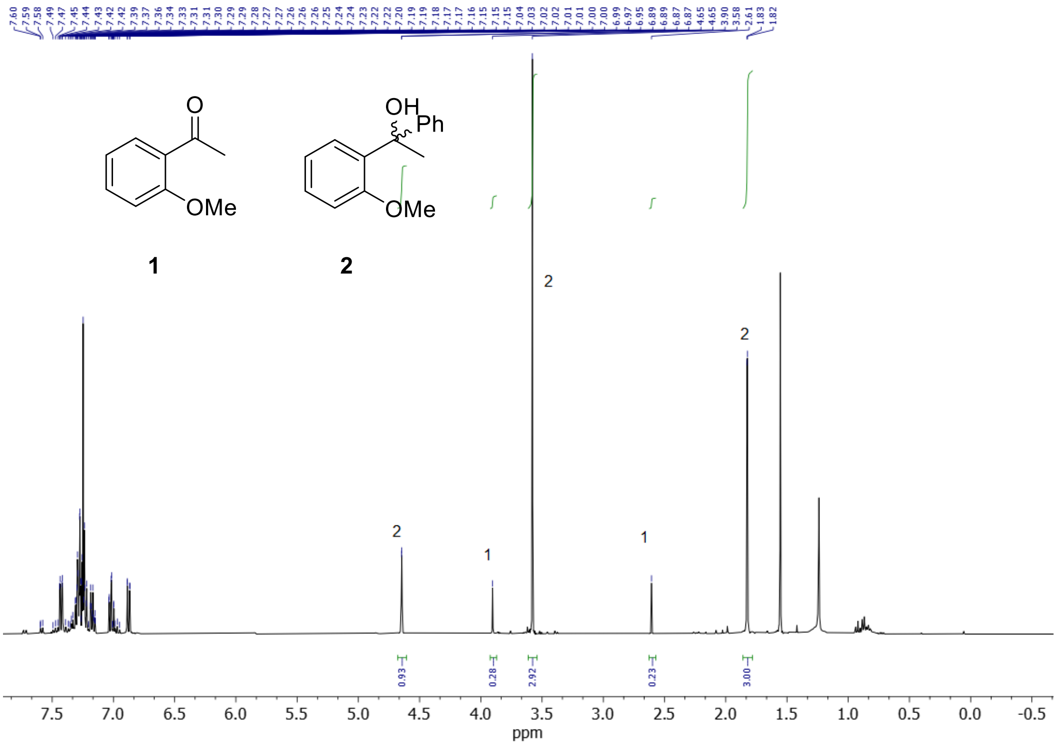

Figure S34. ^{1}H NMR spectrum of the crude product obtained using a $PhLi_{gel}$ block that was stored in a vial under an inert atmosphere closed with a rubber septum for 3 days followed by the reaction with 2′-methoxyacetophenone **1** ($CDCl_3$, 298 K, 400 MHz) – Fig. 3B, Entry 4 (Table S8, Entry 4).

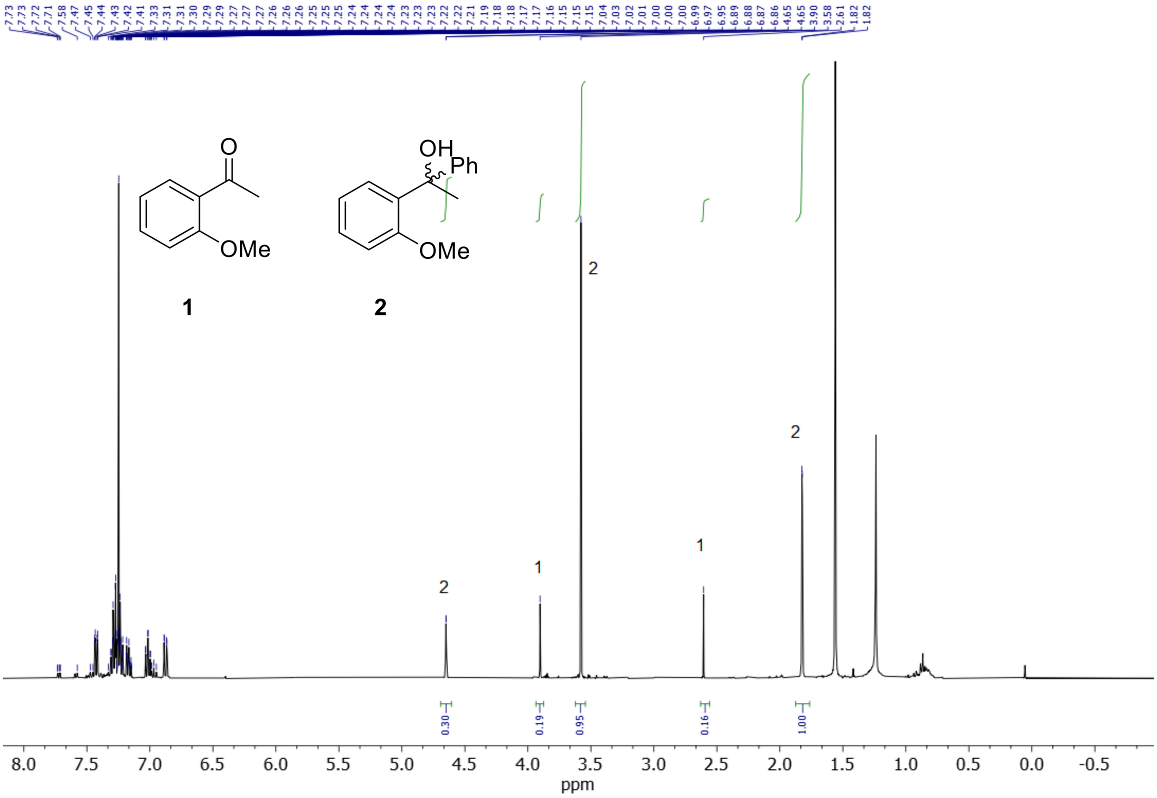

Figure S35. ^{1}H NMR spectrum of the crude product obtained using a PhLi$_{gel}$ block that was stored in a vial under an inert atmosphere closed with a rubber septum for 7 days followed by the reaction with 2′-methoxyacetophenone **1** ($CDCl_3$, 298 K, 400 MHz) – Fig. 3B, Entry 5 (Table S8, Entry 5).

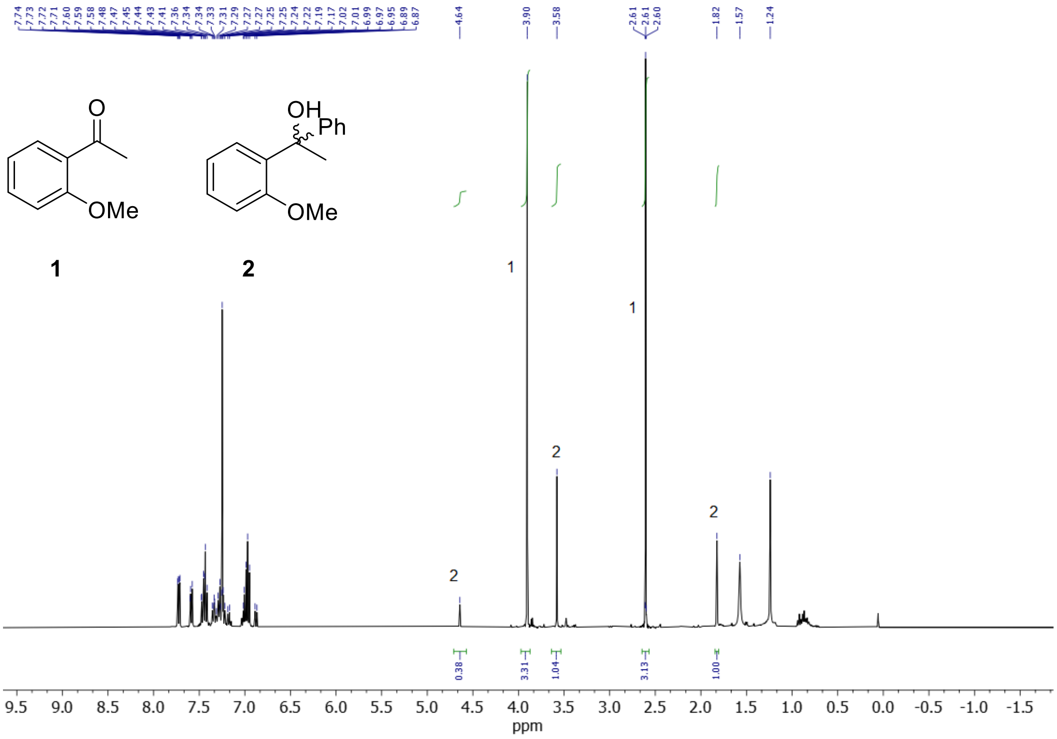

Figure S36. ^{1}H NMR spectrum of the crude product obtained using a PhLi$_{gel}$ block that was stored in a vial under an inert atmosphere closed with a rubber septum for 37 days followed by the reaction with 2′-methoxyacetophenone **1** ($CDCl_3$, 298 K, 400 MHz) – Table S8, Entry 6.

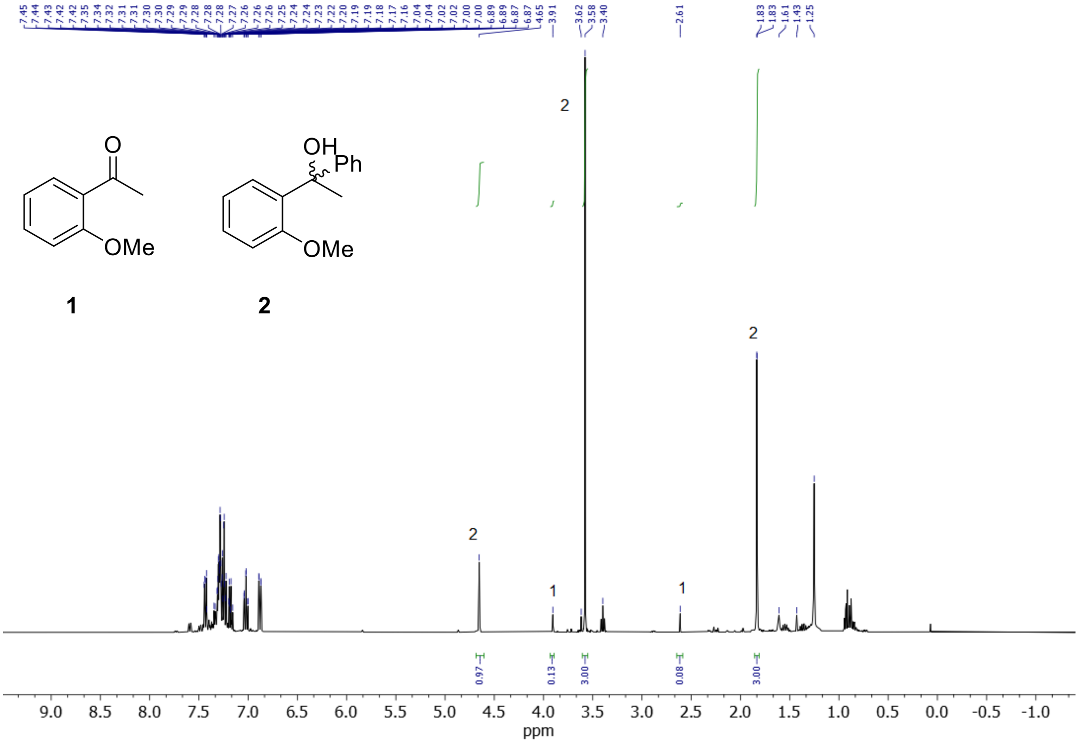

Figure S37. ^{1}H NMR spectrum of the crude product obtained using a $PhLi_{gel}$ block that was stored in a vial under an inert atmosphere closed with a lid for 10 days followed by the reaction with 2′-methoxyacetophenone **1** ($CDCl_3$, 298 K, 400 MHz) – Table S8, Entry 7.

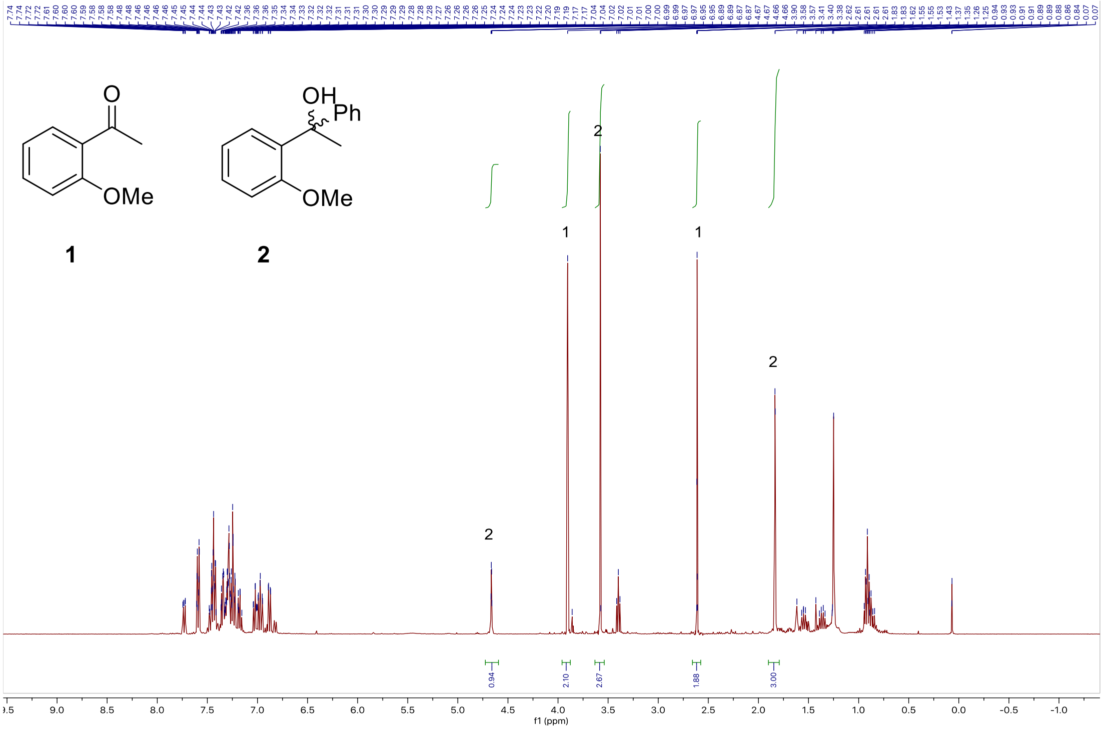

Figure S38. ^{1}H NMR spectrum of the crude product obtained using a $PhLi_{gel}$ block that was stored in a vial under an inert atmosphere closed with a lid for 32 days followed by the reaction with 2′-methoxyacetophenone **1**

($CDCl_3$, 298 K, 400 MHz) – Table S8, Entry 8.

Phenyllithium gel titrations

The amount of PhLi in the gel was determined by titration experiments using butan-2-ol with 1,10-phenanthroline as an indicator.[1] In all titration experiments, 0.5 mL of PhLi (commercially available 1.9 M solution in dibutyl ether) was used together with 250.0 mg of the $C_{36}H_{74}$ gelator.

Table S9. Concentration of PhLi inside the gel determined by titration.

Entry	Preparation	Concentration of PhLi
1	Gel prepared in flask without exposure to air	1.88M
2	Gel prepared in a syringe and further transferred to a flask with approx. 10 s exposure to air	1.70M; 1.76M<br>Average: 1.73M
3	Fresh stock PhLi solution	1.94M; 1.88M<br>Average: 1.91M

Immersion of PhLi$_{gel}$ block in water and subsequent reaction with 2′-methoxyacetophenone 1

A PhLi$_{gel}$ block (0.95 mmol) was prepared according to general procedure F. The PhLi$_{gel}$ block was immersed in water for 30 min (in a beaker). Then, the gel block was removed from the water and carefully dried with a paper towel. The PhLi$_{gel}$ block was used in a reaction with 2′-methoxyacetophenone **1** according to general procedure G. The conversion to **2** was 49% (Fig. 3B, entry 6).

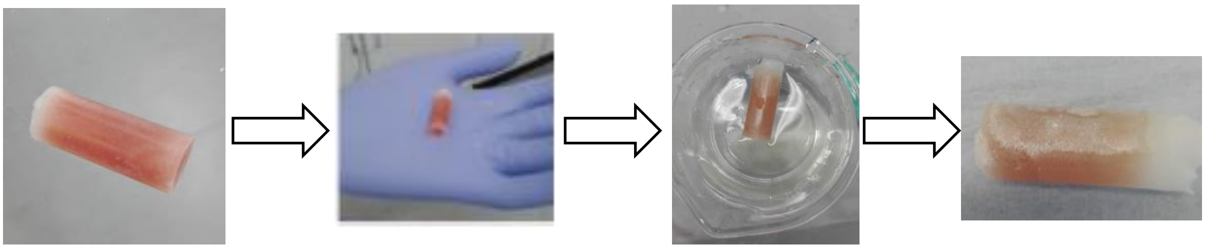

30 min

Figure S39. Immersion of a PhLi$_{gel}$ block in water for 30 min.

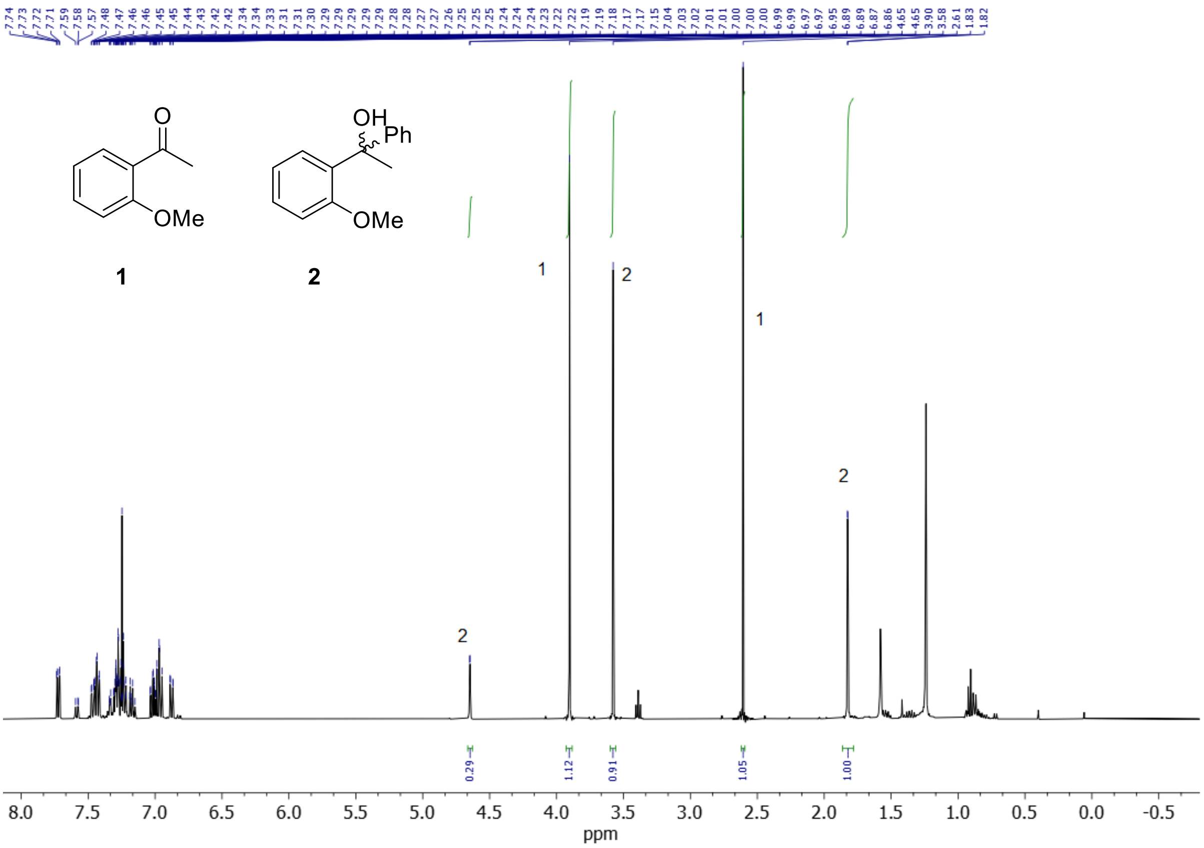

Figure S40. ^{1}H NMR spectrum of the product obtained using a PhLi$_{gel}$ block that was immersed in water for 30 min followed by the reaction with 2′-methoxyacetophenone **1** ($CDCl_3$, 298 K, 400 MHz) – Fig. 3B, entry 6.

Immersion of a more robust PhLi$_{gel}$ block (33% wt/vol $C_{36}C_{74}$ gelator) in water and subsequent reaction with 2′-methoxyacetophenone 1

According to general procedure F, a more robust PhLi$_{gel}$ block (0.95 mmol) was prepared using a larger amount of $C_{36}H_{74}$ gelator (0.50 g, 0.99 mmol), dry dibutyl ether (1 mL) and PhLi (0.5 mL, 1.9 M in dibutyl ether). The PhLi$_{gel}$ block was immersed in water for 30 min (in a beaker). Then, the gel block was removed from the water and carefully dried with a paper towel. The PhLi$_{gel}$ block was used in a reaction with 2′-methoxyacetophenone **1** (0.475 mmol) in dry dibutyl ether (5 mL) according to general procedure G. The conversion to **2** was 69% (Fig. 3B, entry 7).

a) 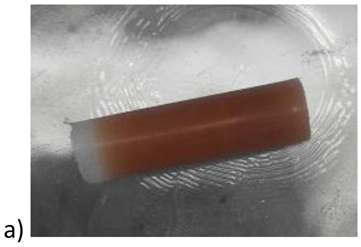 b) 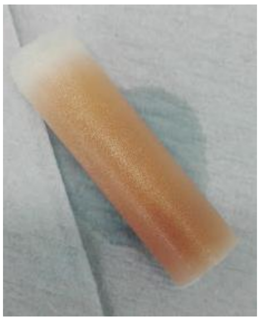

Figure S41. Immersion of a more robust PhLi$_{gel}$ block in water for 30 min: a) before immersion in water; b) after immersion in water for 30 min and drying with a paper towel.

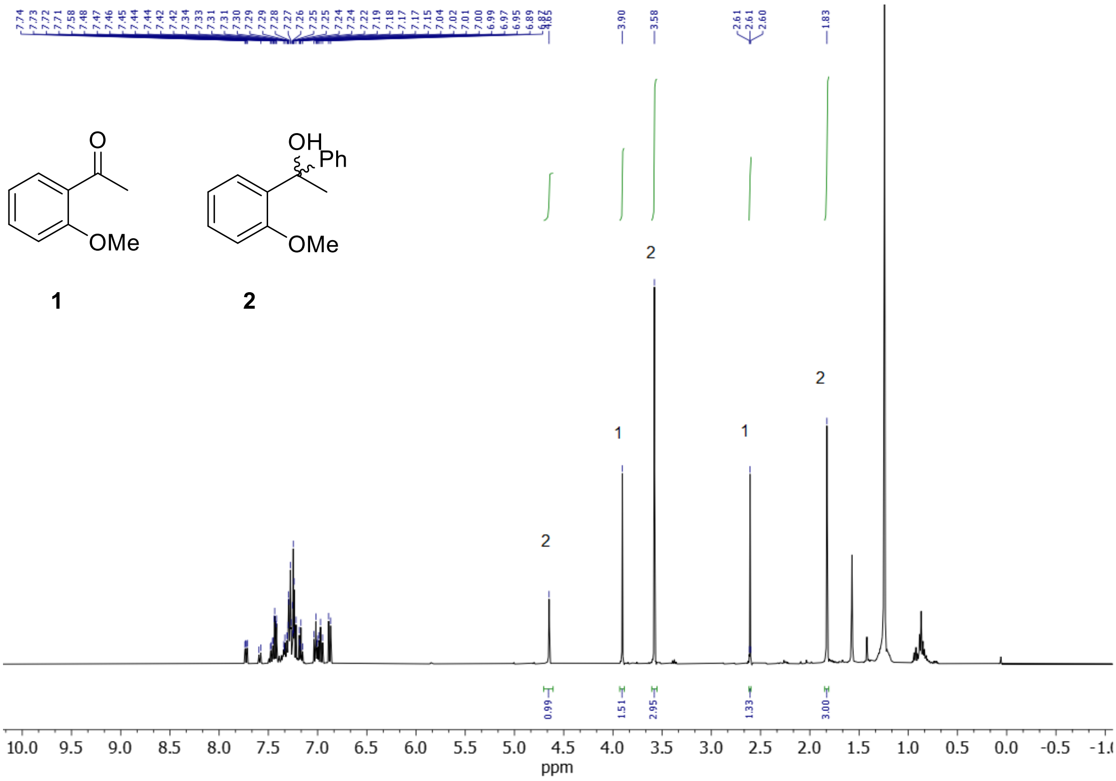

Figure S42. ^{1}H NMR spectrum of the product obtained using a more robust PhLi$_{gel}$ block that was immersed in water for 30 min followed by the reaction with 2′-methoxyacetophenone **1** ($CDCl_3$, 298 K, 400 MHz) – Fig. 3B, entry 7.

Paraffin coating (drill and fill) of a $PhLi_{gel}$ block (16.7% wt/vol $C_{36}C_{74}$ gelator) in water and subsequent reaction with 2′-methoxyacetophenone 1

Paraffin wax (mp 43-95 °C) was melted in a beaker and used to prepare an empty paraffin capsule using a glass rod (Fig. S43a and S43b). This paraffin capsule was filled with a $PhLi_{gel}$ block (prepared from $C_{36}H_{74}$ (166.7 mg, 0.329 mmol, 16.7 %wt/vol), dry dibutyl ether (0.67 mL). and PhLi (0.33 mL, of PhLi – 1.91 M in dibutyl ether) according to general procedure F). The paraffin capsule was sealed with a heated glass rod and quickly immersed in melted paraffin three times (Fig. S43c and S43d). After cooling the paraffin capsule to rt, it was immersed in water for 30 min (in a beaker). Then, the gel block was removed from the water and carefully dried with a paper towel. The paraffin capsule containing the $PhLi_{gel}$ block was used in a reaction with 2′-methoxyacetophenone **1** (0.317 mmol) in dry dibutyl ether (5 mL) according to general procedure G. In this case, it was necessary to use a spatula to break down the paraffin capsule. The conversion to **2** was 84% – see text.

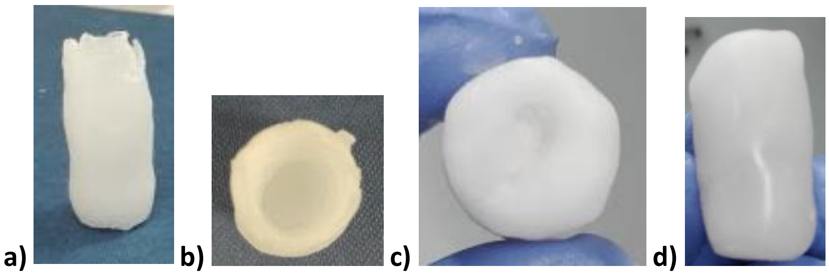

Figure S43. a) and b) empty paraffin capsule; c) and d) paraffin capsule filled with a $PhLi_{gel}$ block and additional wax coating.

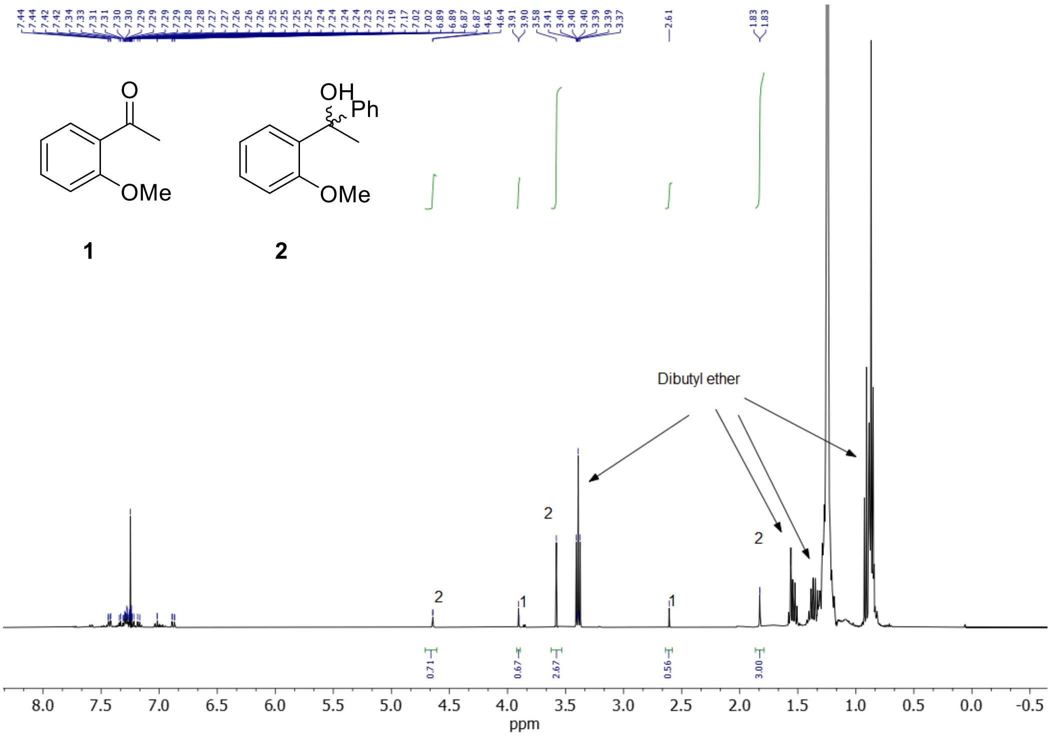

Figure S44. ^1H NMR spectrum of the product obtained using a $PhLi_{gel}$ block inside a paraffin capsule that was immersed in water for 30 min followed by the reaction with 2′-methoxyacetophenone **1** ($CDCl_3$, 298 K, 400 MHz) – see text.

Subdividing a PhLi$_{gel}$ block and subsequent reaction with 2′-methoxyacetophenone 1

A PhLi$_{gel}$ block (0.95 mmol) was prepared according to general procedure F. The PhLi$_{gel}$ block was placed on a dry Petri dish and carefully cut into three equal pieces with a razor blade. Then, each piece of the gel was used in a separate reaction with 2′-methoxyacetophenone **1** (22.5 µL, 0.15 mmol) in dry dibutyl ether (2 mL) according to general procedure G. The conversion to **2** was 96% (Fig. 3B), 97% (Fig. 3B) and 98% (Fig. 3B); average conversion to **2** was 97%.

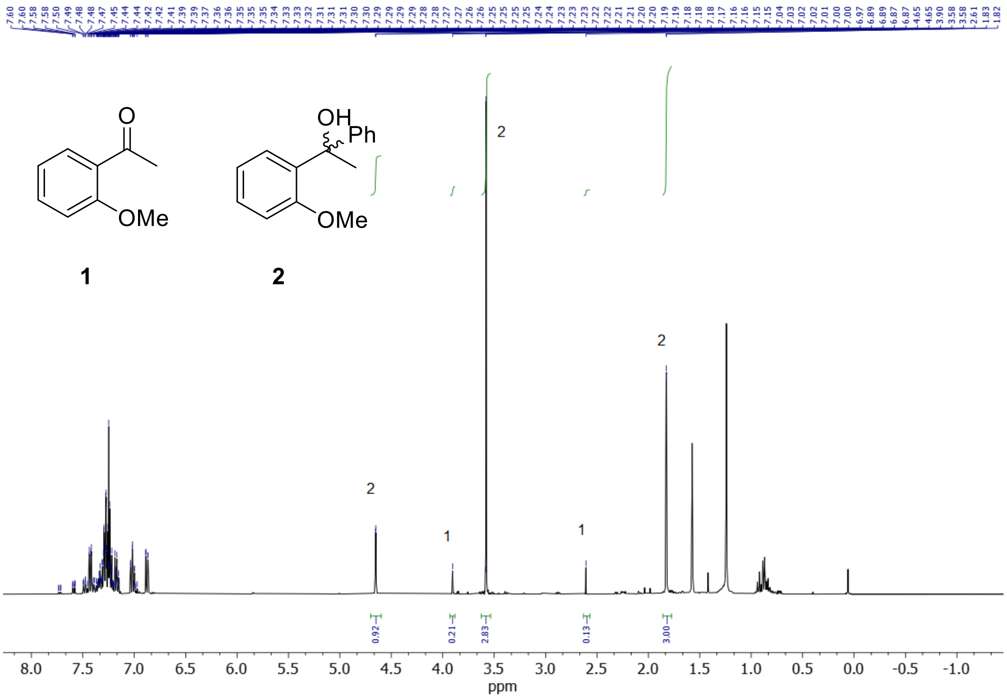

Figure S45. ^{1}H NMR spectrum of the crude product obtained using the first third of the PhLi$_{gel}$ block for the reaction with 2′-methoxyacetophenone **1** ($CDCl_3$, 298 K, 400 MHz). Conversion to **2** 96% (Fig. 3B).

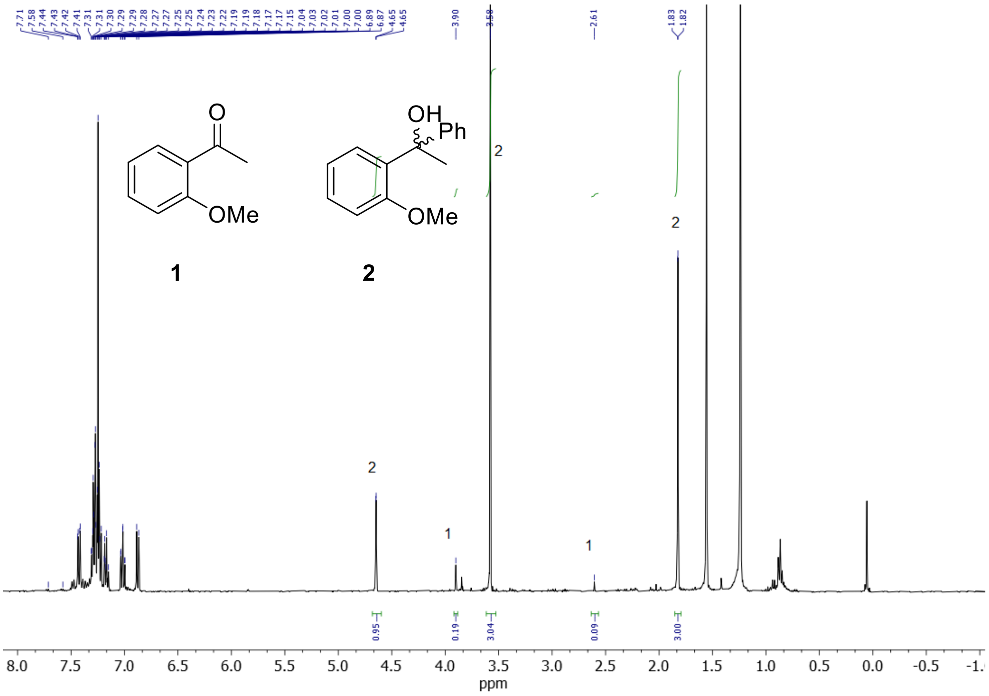

Figure S46. ^{1}H NMR spectrum of the crude product obtained using the second third of the PhLi$_{gel}$ block for the reaction with 2′-methoxyacetophenone **1** ($CDCl_3$, 298 K, 400 MHz). Conversion to **2** 97% (Fig. 3B).

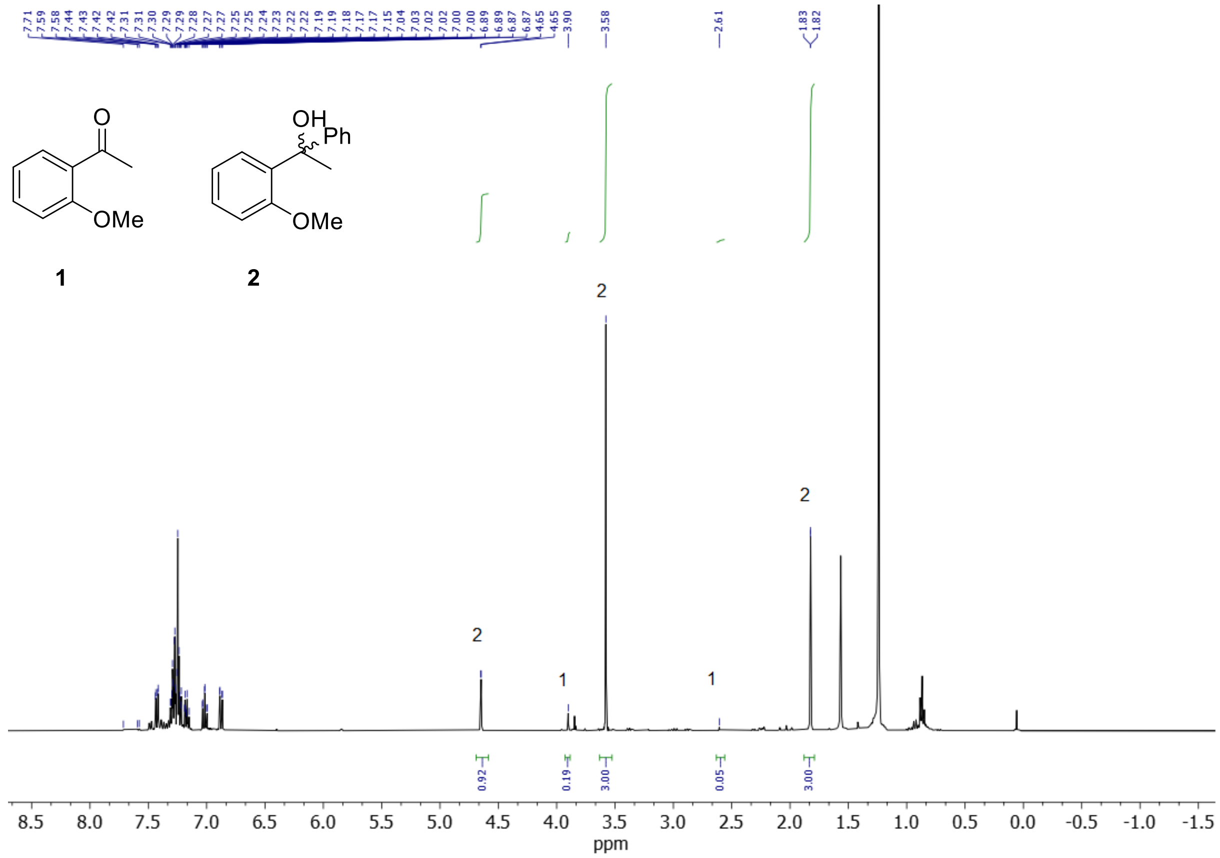

Figure S47. ^{1}H NMR spectrum of the crude product obtained using the third portion of the $PhLi_{gel}$ block for the reaction with 2′-methoxyacetophenone **1** ($CDCl_3$, 298 K, 400 MHz). Conversion to **2** 98% (Fig. 3B).

Subdividing $PhLi_{gel}$ and *n*-$BuLi_{gel}$ blocks (20% wt/vol) and subsequent titration experiments

According to general procedure F, a more robust $PhLi_{gel}$ block (1.9 mmol) was prepared using a larger amount of $C_{36}H_{74}$ gelator (0.60 g, 1.2 mmol, 20% wt/vol), dry dibutyl ether (2 mL) and PhLi (1.0 mL, 1.9 M in dibutyl ether, 1.9 mmol). The upper part of the syringe was carefully cut with scissors and the gel block was removed. The gel block was carefully cut into 3 equal sized blocks using a razor blade. Each block was weighed and placed into a separate vial which had been flushed with nitrogen. The concentration of PhLi in the divided gel blocks was determined by titration experiments using (+)-menthol in THF with 2,2′-bipyridine as an indicator.[4]

Block 1	Block 2	Block 3
Mass: 0.9620 g	Mass: 0.9300 g	Mass: 0.8543 g
Corrected mass:[a] 0.7679 g	Corrected mass: 0.7440 g	Corrected mass: 0.6834 g
$Volume_{PhLi}$:[b] 0.92 mL	$Volume_{PhLi}$: 0.89 mL	$Volume_{PhLi}$: 0.82 mL
$Volume_{menthol}$: 0.80 mL	$Volume_{menthol}$: 0.78 mL	$Volume_{menthol}$: 0.73 mL
Concentration: 0.43 $molL^{-1}$	Concentration: 0.44 $molL^{-1}$	Concentration: 0.44 $molL^{-1}$

[a] Corrected mass = mass of block – mass of gelator in each block. [b] Density of PhLi = 0.835 gmL^{-1} (Aldrich).

According to general procedure F, a more robust n-BuLi$_{gel}$ block (1.9 mmol) was prepared using a larger amount of $C_{36}H_{74}$ gelator (0.64 g, 1.26 mmol, 20% wt/vol), dry dibutyl ether (2 mL) and PhLi (1.0 mL, 1.9 M in dibutyl ether, 1.9 mmol). The upper part of the syringe was carefully cut with scissors and the gel block was removed. The gel block was carefully cut into 3 equal sized blocks using a razor blade. Each block was weighed and placed into a separate vial which had been flushed with nitrogen. The concentration of n-BuLi in the divided gel blocks was determined by titration experiments using (+)-menthol in THF with 2,2'-bipyridine as an indicator.[4]

**Block 1**	**Block 2**	**Block 3**
Mass: 0.8130 g	Mass: 0.8430 g	Mass: 0.8201 g
Corrected mass:[a] 0.6504 g	Corrected mass: 0.6784 g	Corrected mass: 0.6561 g
Volume$_{n\text{-BuLi}}$:[b] 0.96 mL	Volume$_{BuLi}$: 1.0 mL	Volume$_{BuLi}$: 0.96 mL
Volume$_{menthol}$: 0.68 mL	Volume$_{menthol}$: 0.70 mL	Volume$_{menthol}$: 0.70 mL
Concentration: 0.35 molL^{-1}	Concentration: 0.35 molL^{-1}	Concentration: 0.36 molL^{-1}

[a] Corrected mass = mass of block – mass of gelator in each block. [b] Density of n-BuLi = 0.68 gmL^{-1} (Fisher).

General procedure H: Reaction of *n*-BuLi$_{gel}$ block with 2′-methoxyacetophenone 1 using an organolithium gel block

A *n*-BuLi$_{gel}$ block (0.96 mmol) was prepared according to general procedure F. After the specified time and storage conditions (see Table S8), the gel block was carefully placed in a 5 mL round-bottomed flask containing a solution of 2′-methoxyacetophenone **1** (66.1 µL, 0.48 mmol) in dry hexane (2 mL) at room temperature and under air. The mixture was vigorously stirred for 5 min before the reaction was quenched by the addition of water (0.5 mL). The solids in the reaction mixture were removed by filtration using a glass funnel and filter paper. This procedure removed most of the $C_{36}H_{74}$ gelator. The reaction vial and filter paper with the gelator were washed with additional dibutyl ether (3 x 2 mL) and the combined filtrates were dried ($MgSO_4$) and evaporated under reduced pressure to give the crude product. The crude product was analysed by ^{1}H NMR spectroscopy to determine the conversion based on relative integrals of the CH_3 group in the product and starting material. Compound **3** has been previously reported and the spectroscopic data were in agreement.[2]

Table S10 Screening of *n*-BuLi$_{gel}$ block stability under ambient conditions

Entry	Storage conditions	Conversion to **3**[a]
1	30 sec[b]	83%
2	3 days[c]	76%
3	7 days[c]	73%

[a] Conversions determined by ^{1}H NMR spectroscopy using the relative integrals of key signals in product and starting material. [b] The *n*-BuLi$_{gel}$ block was prepared and exposed to air on a petri dish for 30 s.[c] The *n*-BuLi$_{gel}$ block was stored in the syringe used to prepare it under an inert atmosphere for the specified length of time.

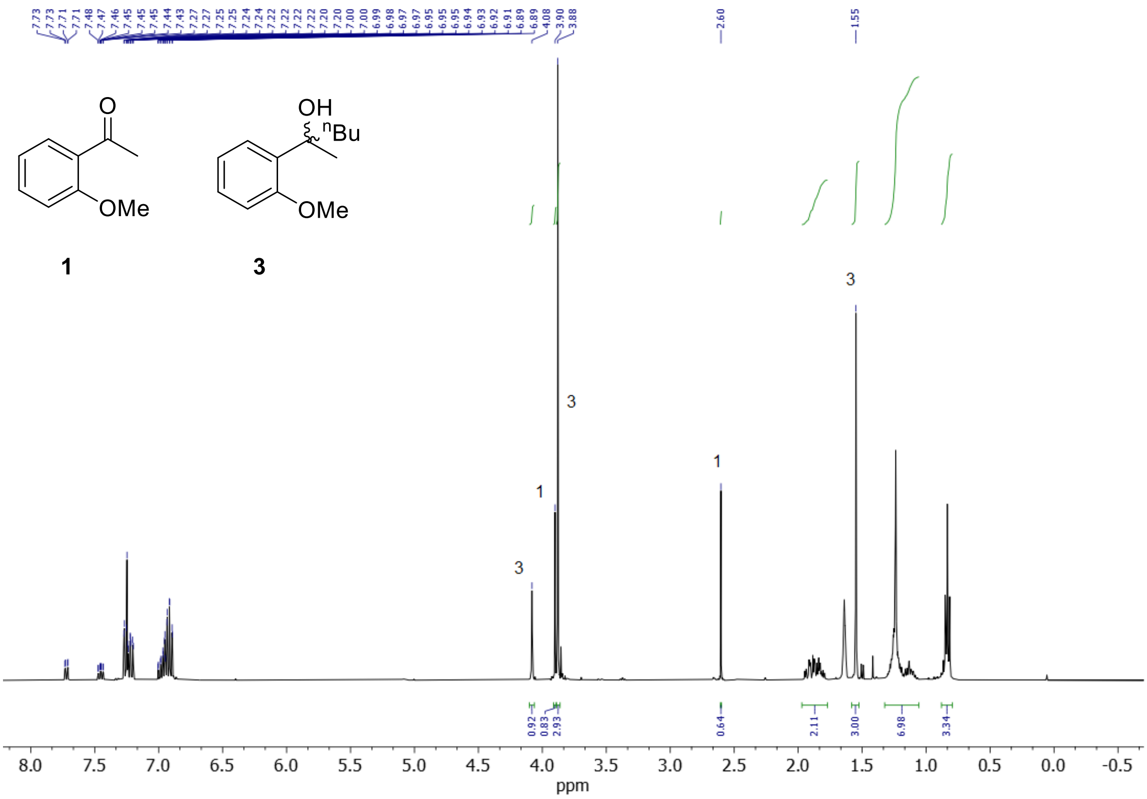

Figure S48. ^{1}H NMR spectrum of the crude product obtained using a *n*-$BuLi_{gel}$ block that was exposed to air on a petri dish for 30 s followed by the reaction with 2′-methoxyacetophenone **1** ($CDCl_3$, 298 K, 400 MHz). Conversion to **3**: 83% (Fig. 3C).

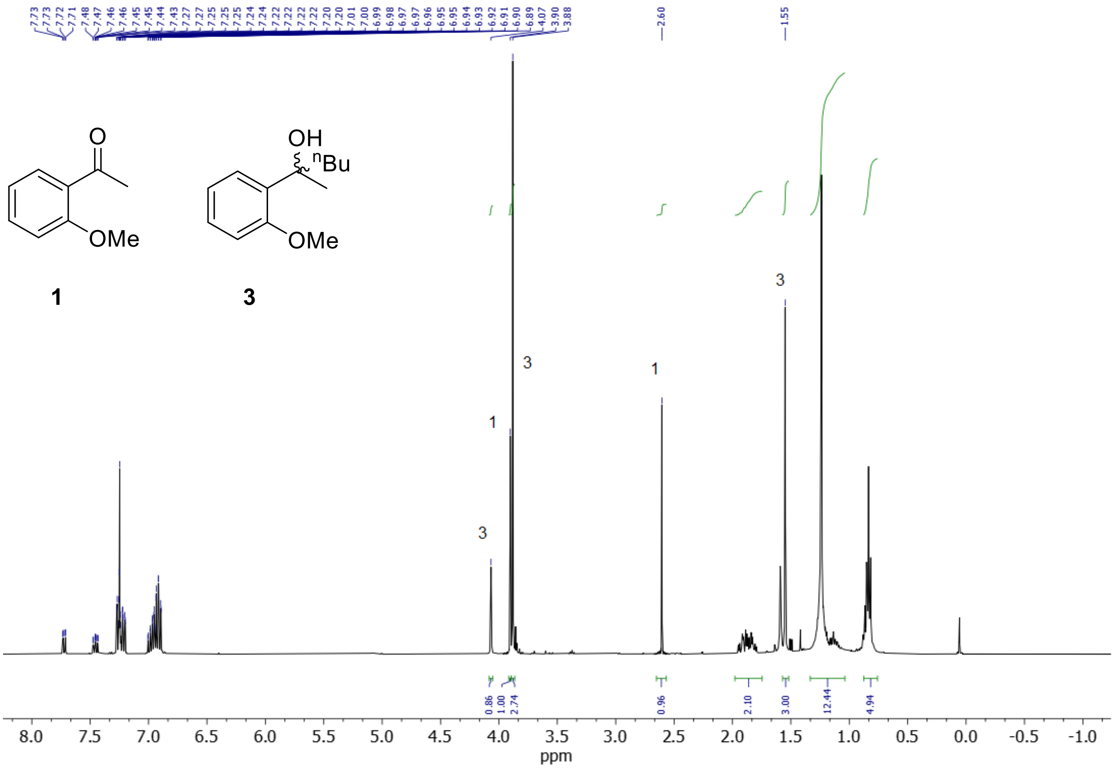

Figure S49. ^{1}H NMR spectrum of the crude product obtained using a *n*-$BuLi_{gel}$ block that was stored in a syringe under an inert atmosphere for 3 days followed by the reaction with 2′-methoxyacetophenone **1** ($CDCl_3$, 298 K, 400 MHz). Conversion to **3**: 76%.

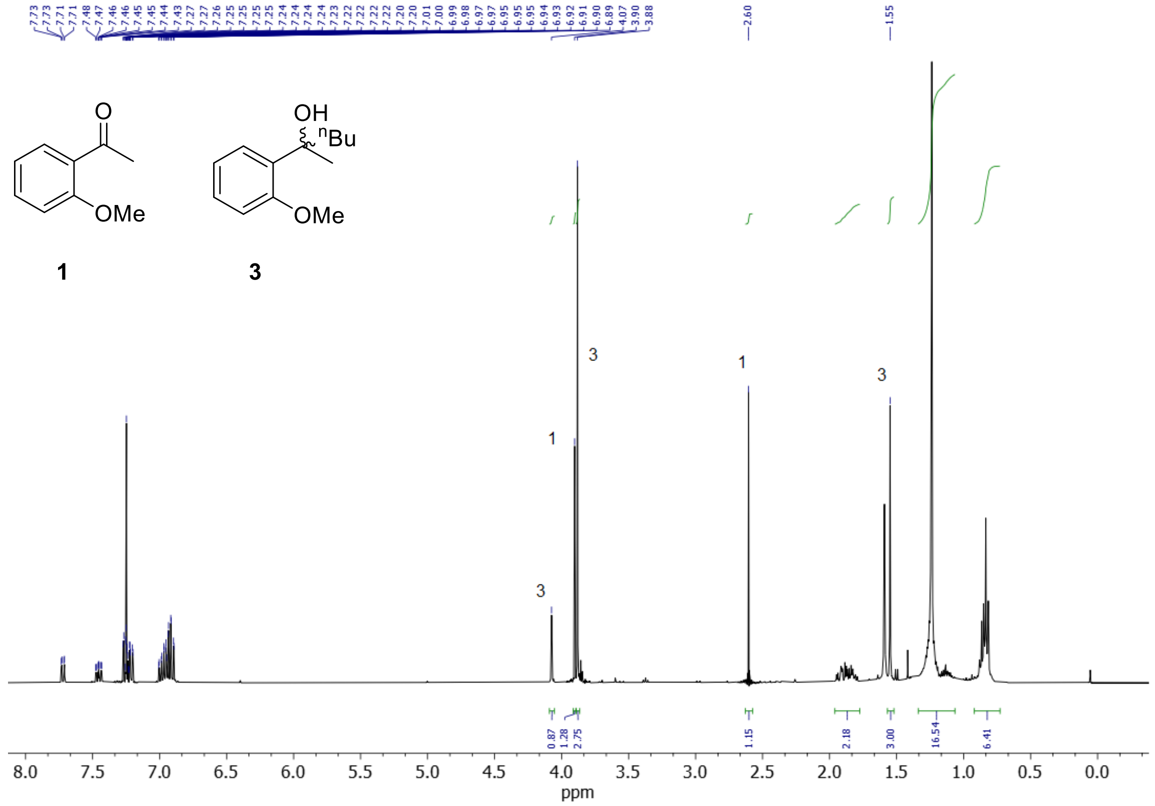

Figure S50. ^{1}H NMR spectrum of the crude product obtained using a *n*-BuLi$_{gel}$ block that was stored in a syringe under an inert atmosphere for 7 days followed by the reaction with 2′-methoxyacetophenone **1** ($CDCl_3$, 298 K, 400 MHz). Conversion to **3**: 73%.

Reaction of a $PhLi_{gel}$ block with benzophenone 4

A $PhLi_{gel}$ block (0.95 mmol) was prepared according to general procedure F. After brief exposure to air on a petri dish (10 s), the gel block was carefully placed in a 5 mL round-bottom flask containing benzophenone **4** (0.0894 g, 0.49 mmol) in dry dibutyl ether (2 mL) at room temperature and under air. The mixture was vigorously stirred for 5 min before the reaction was quenched by the addition of water (0.5 mL). The solids in the reaction mixture were removed by filtration using a glass funnel and filter paper. This procedure removed most of the $C_{36}H_{74}$ gelator. The reaction vial and filter paper with the gelator were washed with additional dibutyl ether (3 x 2 mL) and the combined filtrates were dried ($MgSO_4$) and evaporated under reduced pressure to give the crude product. The crude product was analysed by ^1H NMR spectroscopy to determine the conversion to **5** (99%) based on relative integrals of key signals in the product and starting material. To fully remove the remaining gelator, the crude product was purified by column chromatography (silica gel, eluent: hexane →hexane:dichloromethane 1:1). Compound **5** was isolated as white crystals (0.126 g, 98%). Compound **5** has been previously reported and the spectroscopic data were in agreement.[2]

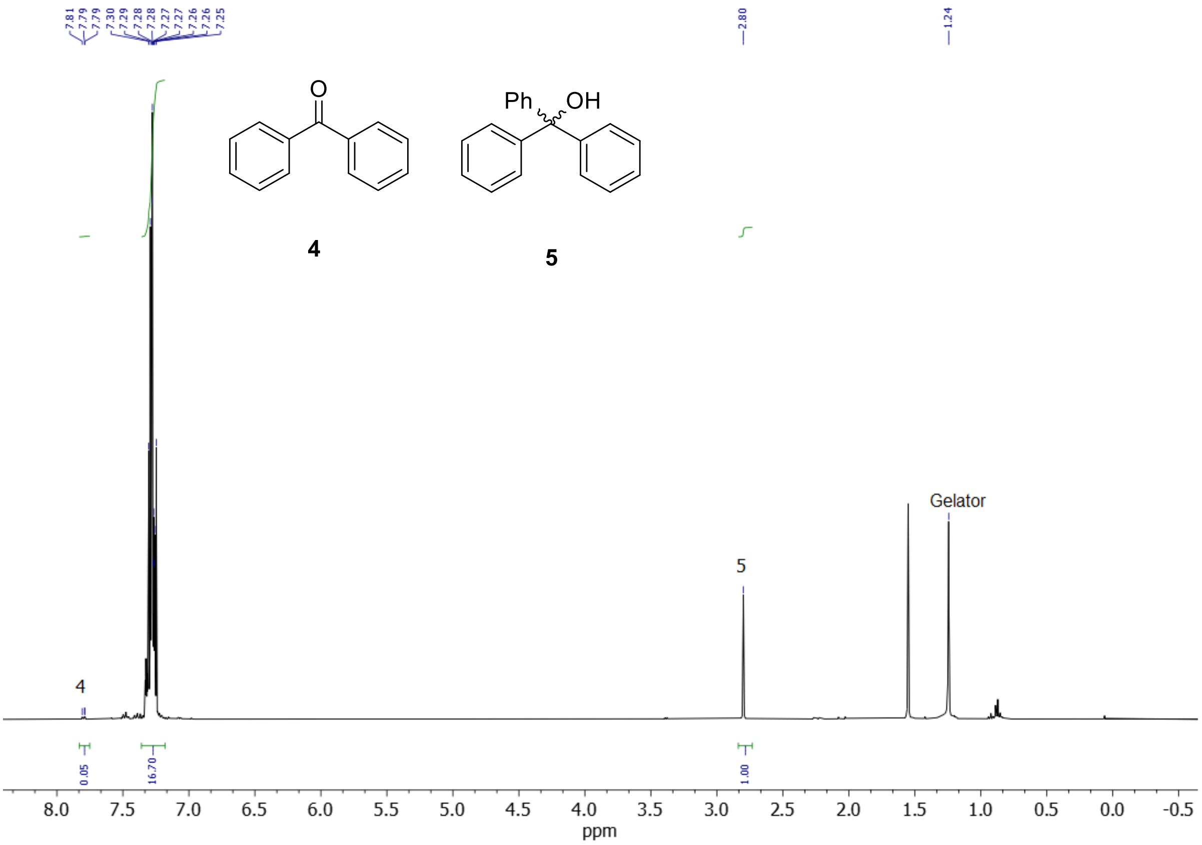

Figure S51. ^1H NMR spectrum of the crude product obtained using a $PhLi_{gel}$ block for the reaction with benzophenone **4** ($CDCl_3$, 298 K, 400 MHz). Conversion to **5**: 99% (Fig. 3C).

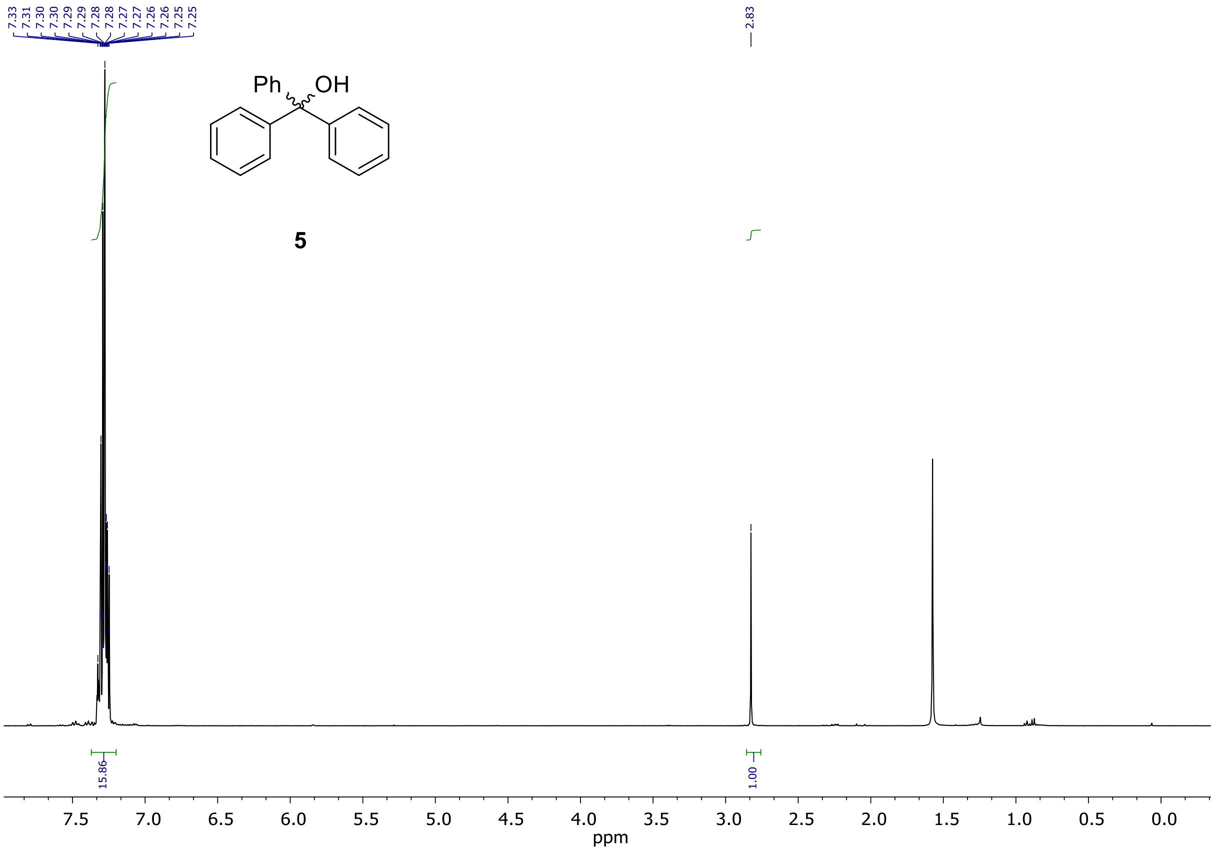

Figure S52. ^{1}H NMR spectrum of triphenylmethanol **5** obtained using a $PhLi_{gel}$ block for the reaction with benzophenone **4** after purification by column chromatography ($CDCl_3$, 298 K, 400 MHz) (Fig. 3C).

Reaction of a *n*-BuLi$_{gel}$ block with benzophenone 4

A *n*-BuLi$_{gel}$ block (0.96 mmol) was prepared according to general procedure F. After brief exposure to air on a petri dish (10 s), the gel block was carefully placed in a 5 mL round-bottom flask containing benzophenone **4** (0.0875 g, 0.48 mmol) in dry hexane (2 mL) at room temperature and under air. The mixture was vigorously stirred for 5 min before the reaction was quenched by the addition of water (0.5 mL). The solids in the reaction mixture were removed by filtration using a glass funnel and filter paper. This procedure removed most of the $C_{36}H_{74}$ gelator. The reaction vial and filter paper with the gelator were washed with additional dibutyl ether (3 x 2 mL) and the combined filtrates were dried ($MgSO_4$) and evaporated under reduced pressure to give the crude product. The crude product was analysed by ^{1}H NMR spectroscopy to determine the conversion (75%) based on relative integrals of key signals in the product and starting material. Compounds **8** and **S1** has been previously reported and the spectroscopic data were in agreement.[2]

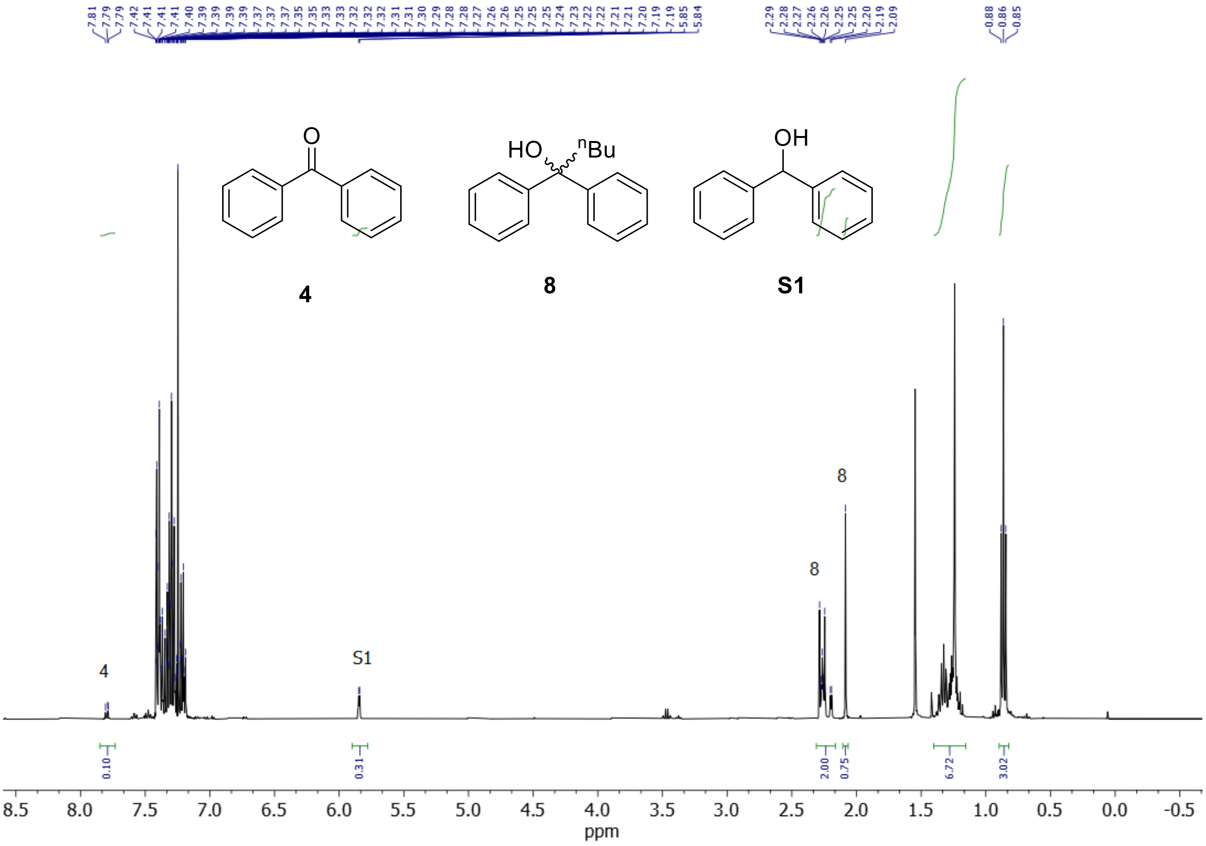

Figure S53. ^{1}H NMR spectrum of the crude product obtained using a *n*-BuLi$_{gel}$ block for the reaction with benzophenone **4** ($CDCl_3$, 298 K, 400 MHz). 1% **4**; 75% **8**; 24% **S1**

Reaction of a PhLi$_{gel}$ block with *N*-benzylideneaniline 6

A PhLi$_{gel}$ block (0.95 mmol) was prepared according to general procedure F. After brief exposure to air on a petri dish (10 s), the gel block was carefully placed in a 5 mL round-bottom flask containing *N*-benzylideneaniline **6** (0.0861 g, 0.475 mmol) in dry dibutyl ether (2 mL) at room temperature and under air. The mixture was vigorously stirred for 5 min before the reaction was quenched by the addition of water (0.5 mL). The solids in the reaction mixture were removed by filtration using a glass funnel and filter paper. This procedure removed most of the $C_{36}H_{74}$ gelator. The reaction vial and filter paper with the gelator were washed with additional dibutyl ether (3 x 2 mL) and the combined filtrates were dried ($MgSO_4$) and evaporated under reduced pressure to give the crude product. The crude product was analysed by ^1H NMR spectroscopy to determine the conversion to **7** (99%) based on relative integrals of key signals in the product and starting material. Compound **7** has been previously reported and the spectroscopic data were in agreement.[3]

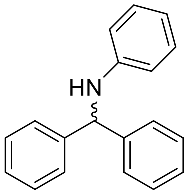

7

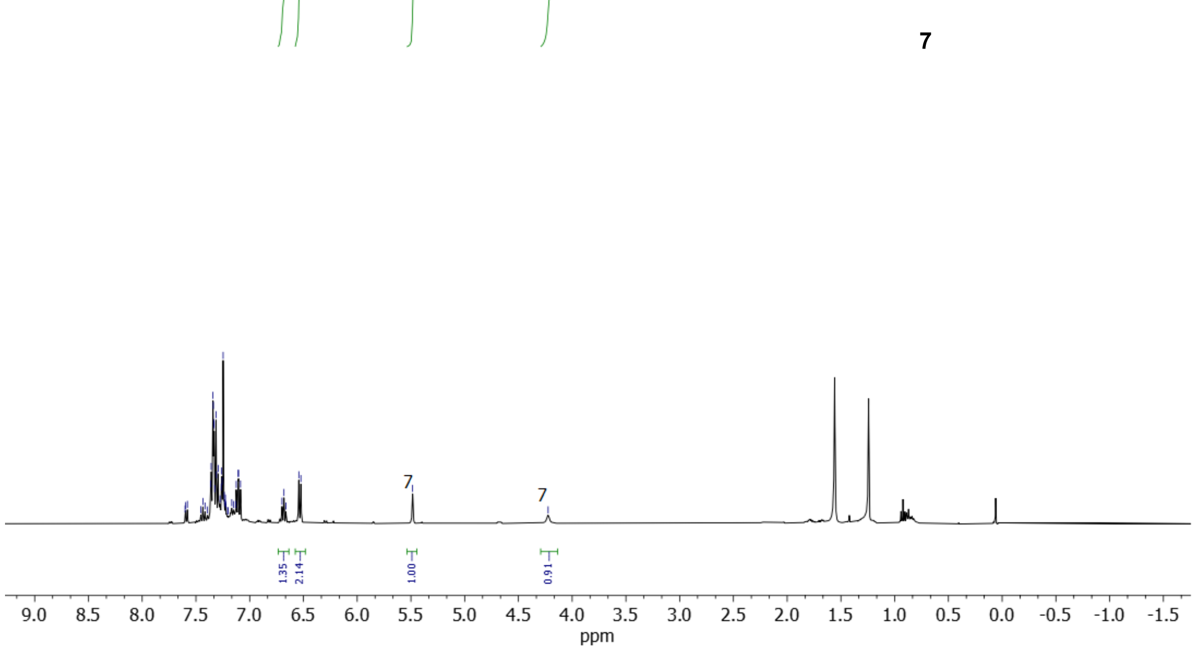

Figure S54. ^1H NMR spectrum of the crude product obtained using a PhLi$_{gel}$ block for the reaction with *N*-benzylideneaniline **6** ($CDCl_3$, 298 K, 400 MHz). Conversion to **7**: 99% (Fig. 3C). No evidence of starting materiál **6**.

Reaction of a *n*-BuLi$_{gel}$ block with *N*-benzylideneaniline 6

A *n*-BuLi$_{gel}$ block (0.96 mmol) was prepared according to general procedure F. After brief exposure to air on a petri dish (10 s), the gel block was carefully placed in a 5 mL round-bottom flask containing *N*-benzylideneaniline **6** (0.0870 g, 0.48 mmol) in dry hexane (2 mL) at room temperature and under air. The mixture was vigorously stirred for 5 min before the reaction was quenched by the addition of water (0.5 mL). The solids in the reaction mixture were removed by filtration using a glass funnel and filter paper. This procedure removed most of the $C_{36}H_{74}$ gelator. The reaction vial and filter paper with the gelator were washed with additional dibutyl ether (3 x 2 mL) and the combined filtrates were dried ($MgSO_4$) and evaporated under reduced pressure to give the crude product. The crude product was analysed by ^{1}H NMR spectroscopy to determine the conversion (>98%) based on relative integrals of key signals in the product and starting material. Compound **9** has been previously reported and the spectroscopic data were in agreement.[3]

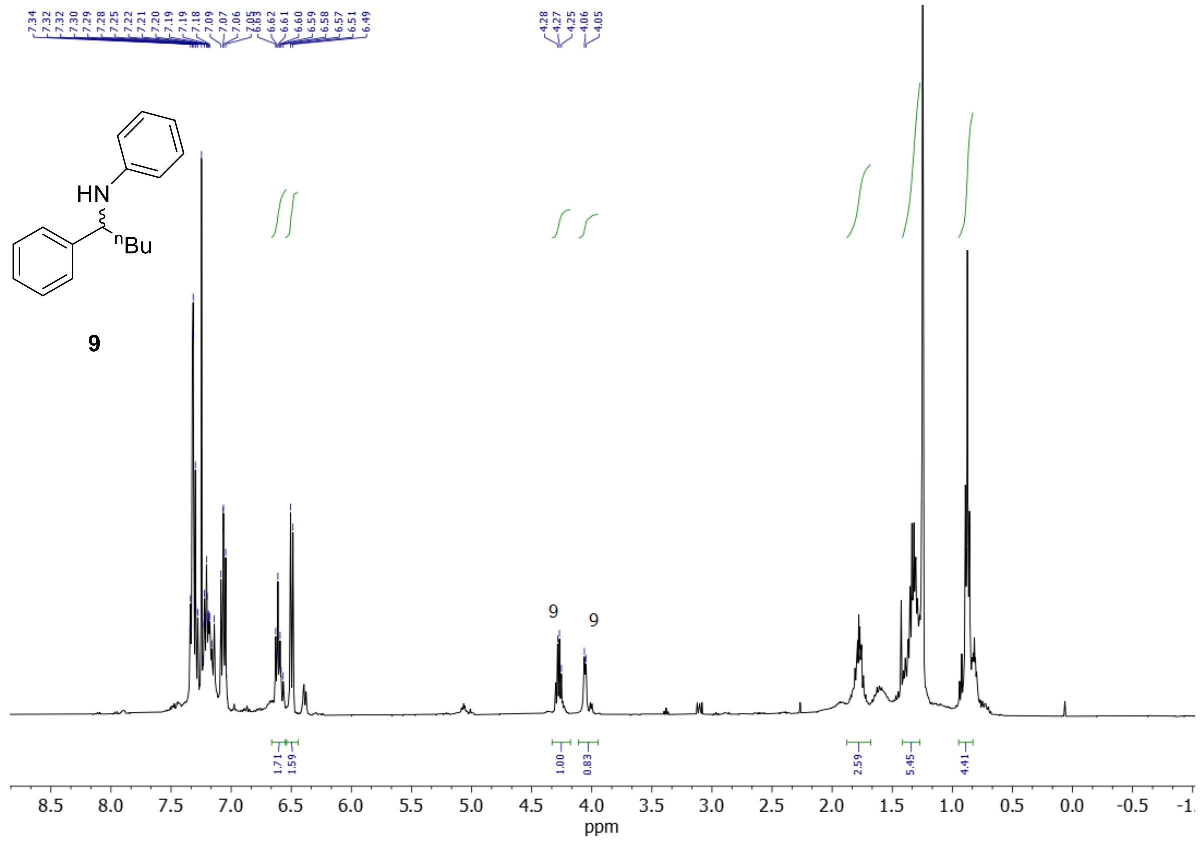

Figure S55. ^{1}H NMR spectrum of the crude product obtained using a *n*-BuLi$_{gel}$ block for the reaction with *N*-benzylideneaniline **6** (CDCl$_3$, 298 K, 400 MHz). Conversion to **9**: >98% (Fig. 3C). No evidence of starting material **6**.

5. Synthetic Applications of the $PhLi_{gel}$ and *n*-$BuLi_{gel}$ Blocks (Results in Fig. 4)

Reaction of a $PhLi_{gel}$ block with benzonitrile 10 to give ketone 4 (Fig. 4A)

A $PhLi_{gel}$ block (0.95 mmol) was prepared according to general procedure F. After brief exposure to air (10 s), the gel block was carefully placed in a 5 mL round-bottom flask containing benzonitrile **10** (49 µL, 0.475 mmol) in dry dibutyl ether (2 mL) at room temperature and under air. The mixture was vigorously stirred for 5 min before the reaction was quenched by the addition of water (0.5 mL). The mixture was transferred to a 50 mL round-bottomed flask and 2 M $HCl_{(aq)}$ (5 mL) was added. The resulting mixture was stirred vigorously and heated at 100 °C for 30 min. After cooling to room temperature, the mixture was extracted with diethyl ether, washed with sat. $NaHCO_{3(aq)}$ (3 x 20 mL) and water (3x20 mL), dried ($MgSO_4$) and evaporated under reduced pressure to give the crude product. The solids in the reaction mixture were removed by filtration using a glass funnel and filter paper. This procedure removed most of the $C_{36}H_{74}$ gelator. The reaction vial and filter paper with the gelator were washed with additional dibutyl ether (3 x 2 mL) and the combined filtrates were dried ($MgSO_4$) and evaporated under reduced pressure to give the crude product. The crude product was analysed by ^1H NMR spectroscopy with dry DMF (40 µL, 0.5166 mmol) as an external standard to determine the yield of **4** (96%). Compound **4** has been previously reported and the spectroscopic data were in agreement.[5]

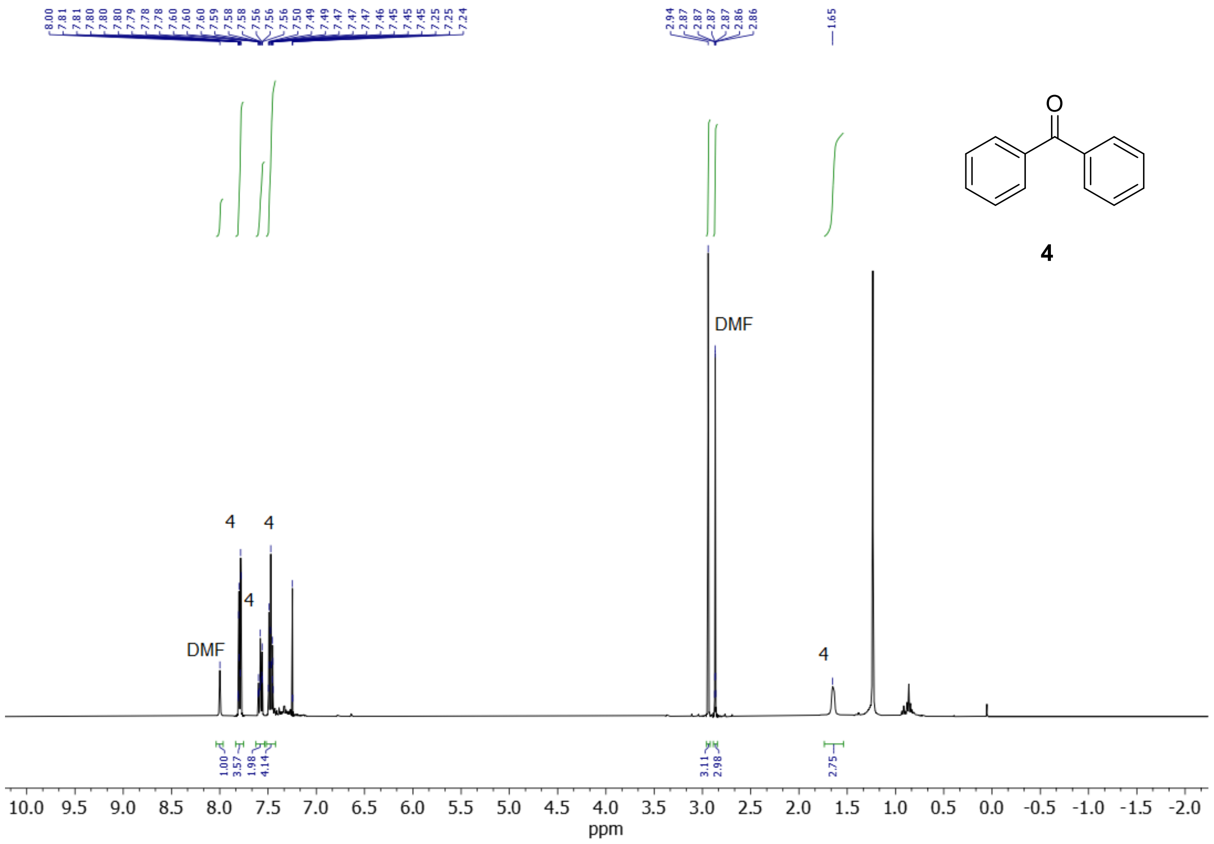

Figure S56. ^1H NMR spectrum of the crude product obtained using a $PhLi_{gel}$ block for the reaction with benzonitrile **10** ($CDCl_3$, 298 K, 400 MHz) with DMF (0.5166 mmol) as an external standard. Fig. 4A.

Reaction of a *n*-BuLi$_{gel}$ block with benzonitrile 10 to give ketone 11 (Fig. 4A)

A *n*-BuLi$_{gel}$ block (0.96 mmol) was prepared according to general procedure F. After brief exposure to air (10 s), the gel block was carefully placed in a 5 mL round-bottom flask containing benzonitrile **10** (49.5 µL, 0.48 mmol) in dry hexane (2 mL) at room temperature and under air. The mixture was vigorously stirred for 5 min before the reaction was quenched by the addition of water (0.5 mL). The mixture was transferred to a 50 mL round-bottomed flask and 2 M $HCl_{(aq)}$ (5 mL) was added. The resulting mixture was stirred vigorously and heated at 100 °C for 30 min. After cooling to room temperature, the mixture was extracted with diethyl ether, washed with sat. $NaHCO_{3(aq)}$ (3 x 20 mL) and water (3x20 mL), dried ($MgSO_4$) and evaporated under reduced pressure to give the crude product. The crude product was analysed by ^{1}H NMR spectroscopy with dry DMF (40 µL, 0.5166 mmol) as an external standard to determine the yield of **11** (87%). Compound **11** has been previously reported and the spectroscopic data were in agreement.[5]

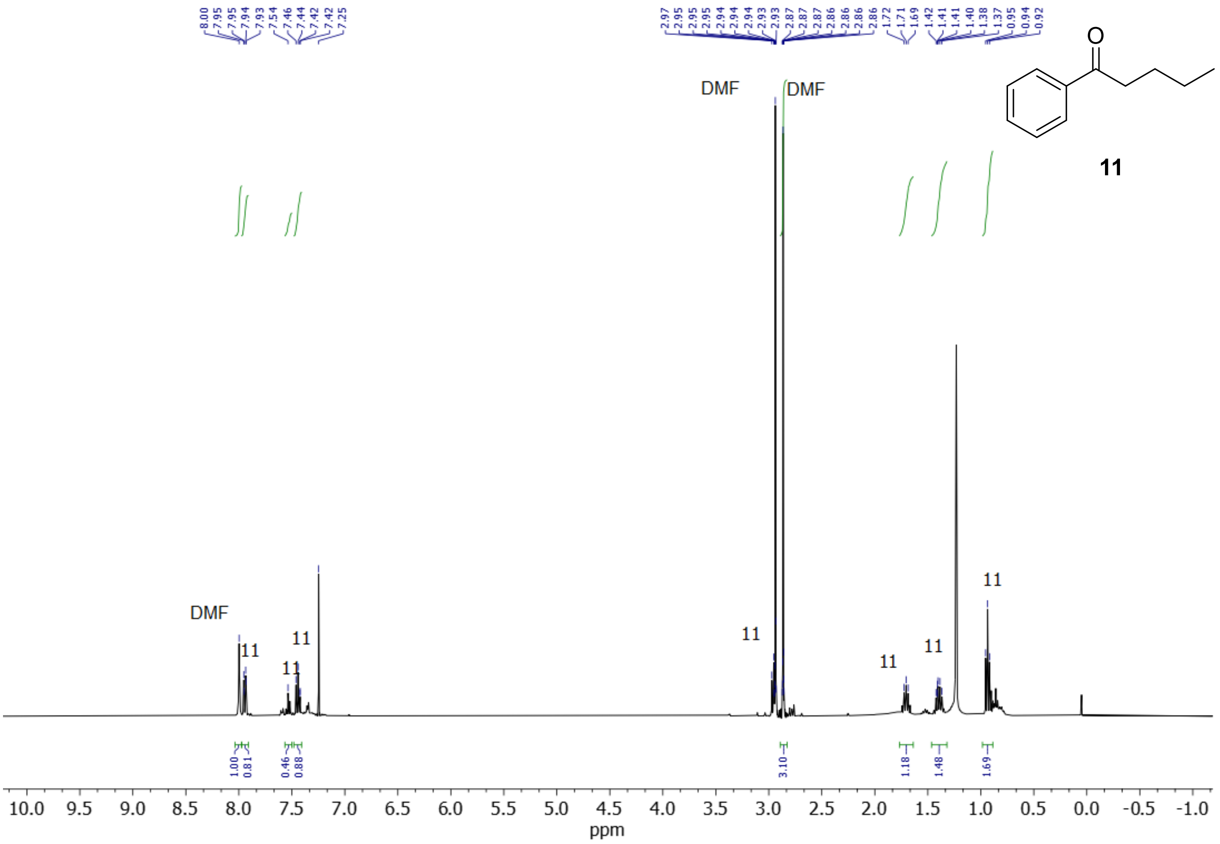

Figure S57. ^{1}H NMR spectrum of the crude product obtained using a *n*-BuLi$_{gel}$ block for the reaction with benzonitrile **10** ($CDCl_3$, 298 K, 400 MHz) with DMF (0.5166 mmol) as an external standard. Fig. 4A.

Two-step Synthesis of Orphenadrine 14 (Fig. 4B)

Large-scale procedure for preparation of a $PhLi_{gel}$ block

A 25 mL round-bottomed flask was dried in the oven and allowed to cool under a nitrogen atmosphere. The flask was charged with the gelator $C_{36}H_{74}$ (2.5 g, 4.9 mmol, 16.7% wt/vol), sealed with a rubber septum and flushed with nitrogen via a needle for 5 min. Anhydrous and degassed dibutyl ether (10 mL) was added through the septum followed by the addition of PhLi (5 mL, 1.91 M in dibutyl ether, 9.5 mmol). The flask (kept under a nitrogen atmosphere using a balloon) was carefully heated until all of the gelator had dissolved. The hot hydrosol was quickly transferred under a nitrogen atmosphere via a needle into a 20 mL syringe (previously flushed with nitrogen and pre-heated in the oven). The syringe was immediately placed in iced water for 1 min until the organogel formed. The PhLi gel was kept in the syringe under a nitrogen atmosphere prior to use. In order to use the PhLi gel, the upper part of the syringe was carefully cut with scissors and the gel block was removed. Examples of the procedure are shown in Fig. S59.

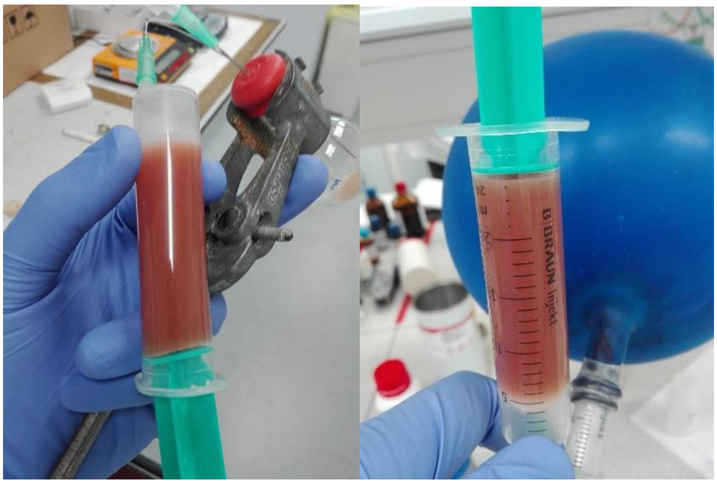

Figure S58. Examples of the large-scale procedure for preparation of a $PhLi_{gel}$ block (9.5 mmol).

Phenyl(2-tolyl)methanol 13

An oven-dried 50 mL round-bottomed flask was charged with 2-methylbenzaldehyde **12** (0.55 mL, 4.75 mmol) and dry THF (15 mL) and the resulting solution was stirred at rt under air. To this solution, a $PhLi_{gel}$ block (9.5 mmol PhLi, prepared according to the large-scale procedure above) was added and the mixture was stirred vigorously at rt for 10 min. The reaction was quenched by addition of MeOH (3 mL) and the mixture was filtered through filter paper to remove the solids (gelator). The filtrate was washed with water (3 x 30 mL) and the organic layer was dried ($MgSO_4$), filtered and evaporated under reduced pressure to give the crude product. Purification by column chromatography (SiO_2, hexane → hexane:EtOAc, 4:1) gave phenyl(2-tolyl)methanol **13** (0.8952 g, 95%) as a white/yellow solid. The recorded spectroscopic data correlate with those reported in the literature.[6]

^{1}H NMR ($CDCl_3$, 400 MHz, 293 K): δ = 7.55-7.50 (m, 1H), 7.40-7.14 (m, 8H), 5.96 (br s, 1H), 2.51 (br s, 1H), 2.25 (s, 3H). ^{13}C NMR ($CDCl_3$, 101 MHz, 293 K): δ = 143.0, 141.6, 135.5, 130.7, 128.6, 127.7 (2x), 127.3, 126.4, 126.3, 73.4, 19.5 .

N,*N*-Dimethyl-2-(phenyl(*o*-tolyl)methoxy)ethanamine – Orphenadrine 14

KOH (2.533 g, 45.15 mmol) was ground up using a pestle and mortar and added to a stirred suspension of phenyl(2-tolyl)methanol **13** (0.8952 g, 4.52 mmol) and 2-(*N,N*-dimethylamino)ethyl chloride (1.301 g, 9.03 mmol) in DMSO (13 mL) at rt. The reaction mixture was stirred at rt for 16 hours. The reaction mixture was diluted with diethyl ether (10 mL) and $NaOH_{(aq)}$ (1 M, 30 mL) was added and the mixture was vigorously stirred for 30 min.

The organic layer was separated and the aqueous layer was extracted with diethyl ether (3 x 20 mL). The combined organic layers were dried ($MgSO_4$), filtered and evaporated under reduced pressure to give the crude product. Purification by column chromatography (SiO_2, hexane:EtOAc:Et_3N, 5:1:0.1) gave Orphenadrine **14** (0.873 g, 72%) as a yellow oil. The recorded spectroscopic data correlate with those reported in literature.[6]

^{1}H NMR ($CDCl_3$, 400 MHz, 293 K): δ = 7.49-7.44 (m, 1H), 7.40-7.11 (m, 8H), 5.56 (s, 1H), 3.64-3.54 (m, 2H), 2-64-2.58 (m, 2H), 2.30-2.26 (m, 9H). ^{13}C NMR ($CDCl_3$, 101 MHz, 293 K): δ = 141.3, 140.0, 136.0, 130.6, 128.4, 127.7, 127.5 (2x), 127.2, 126.1, 81.5, 67.8, 59.2, 46.2, 19.6.

Bis(4-methoxyphenyl)methanol 17 (Fig. 4C)

An oven-dried 10 mL Schlenk flask was sealed with a rubber septum and filled with nitrogen. 4-Bromoanisole **15** (137.8 µL, 1.1 mmol) and dry THF (4 mL) were added through the septum and the solution was cooled to –78 °C. To this mixture, a *n*-$BuLi_{gel}$ block (0.96 mmol of *n*-BuLi, prepared according to general procedure F) was quickly added by removing the rubber septum while keeping the flask under a positive pressure of nitrogen. After the gel block was added, the flask was sealed with the rubber septum. As the gel did not break up immediately during the stirring, it was partly cut into smaller pieces using a long needle. The mixture was left to stir at –78 °C for 45 min and then 4-methoxybenzaldehyde **16** (85.2 µL, 0.7 mmol) was slowly added and the mixture was stirred at -78 °C for 15 min. The reaction mixture was allowed to warm to rt and the reaction was quenched by the addition of MeOH (1 mL). The mixture was diluted with diethyl ether (10 mL) and filtered through a short pad of celite to remove the solids (gelator). The filtrate was washed with water (3 x 30 mL). The organic layer was collected and dried ($MgSO_4$), filtered and evaporated under reduced pressure to give the crude product. Purification by column chromatography (SiO_2, hexane → hexane:EtOAc, 4:1) gave *bis*(4-methoxyphenyl)methanol **17** (0.170 g, 99%) as a white powder. The recorded spectroscopic data correlate with those reported in literature.[7]

^{1}H NMR ($CDCl_3$, 400 MHz, 293 K): δ = 7.30-7.23 (m, 4H), 6.88-6.82 (m, 4H), 5.77 (d, *J* = 3.5 Hz, 1H), 3.78 (s, 6H), 2.07 (d, *J* = 3.5 Hz, 1H). ^{13}C NMR ($CDCl_3$, 101 MHz, 293 K): δ = 159.0, 136.5, 127.9, 113.9, 75.4, 55.4.

Ethene-1,1-diyldibenzene 19 (Fig 4D)

An oven-dried 10 mL Schlenk flask was charged with ethyltriphenylphosphonium bromide **18** (0.343 g, 0.96 mmol), sealed with a rubber septum and filled with nitrogen. Dry THF (4 mL) was added through the septum and the solution was cooled to 0 °C. To this mixture, a *n*-$BuLi_{gel}$ block (0.96 mmol of *n*-BuLi, prepared according to general procedure F) was quickly added by removing the rubber septum while keeping the flask under a positive pressure of nitrogen. After the gel block was added, the flask was sealed with the rubber septum. As the gel did not break up immediately during the stirring, it was partly cut into smaller pieces using a long needle. The mixture was stirred at 0 °C for 30 min and then a solution of benzophenone **4** (0.139 g, 0.74 mmol) in dry THF (1 mL) was slowly added and the mixture was stirred at rt for 18 h. The mixture was diluted with diethyl ether (10 mL) and filtered through filter paper to remove the solids (gelator). The filtrate was washed with brine (3 x 30 mL). The organic layer was collected, dried ($MgSO_4$), filtered and evaporated under reduced pressure to give the crude product. Purification by column chromatography (SiO_2, hexane) gave ethane-1,1-diyldibenzene **19** (0.109 g, 82%) as a colourless oil. The recorded spectroscopic data correlate with those reported in literature.[8]

^{1}H NMR ($CDCl_3$, 400 MHz, 293 K): δ = 7.40-7.29 (m, 10H), 5.46 (s, 2H). ^{13}C NMR ($CDCl_3$, 101 MHz, 293 K): δ = 150.1, 141.6, 128.4, 128.3, 127.8, 114.4.

Methyl 2-phenylpentanoate 23 (Fig. 4E)

An oven-dried 10 mL Schlenk tube flask was sealed with a rubber septum and filled with nitrogen. Freshly distilled di-*iso*-propylamine **20** (0.16 mL, 1.1 mmol) and dry THF (3 mL) were added through the septum and the solution was cooled to 0 °C. To this mixture, a *n*-$BuLi_{gel}$ block (0.96 mmol of *n*-BuLi, prepared according to general

procedure F) was quickly added by removing the rubber septum while keeping the flask under a positive pressure of nitrogen. After the gel block was added, the flask was sealed with the rubber septum. As the gel did not break up immediately during the stirring, it was partly cut into smaller pieces using a long needle. The mixture was stirred at 0 °C for 30 min. Then, the mixture was cooled to –78 °C and methyl 2-phenylacetate **22** (106.3 µL, 0.75 mmol) was slowly added and stirred at -78 °C for 1 hour. Then, *n*-propyl iodide (2.25 mL, 0.22 mL) was added dropwise. The mixture was allowed to warm to rt and was stirred at rt for 3 hours. The reaction was quenched by addition of sat. $NH_4Cl_{(aq)}$ (1 mL). The mixture was diluted with diethyl ether (10 mL) and filtered through filter paper to remove the solids (gelator). The filtrate was washed with water (3 x 30 mL) and brine (3 x 30 mL). The organic layer was collected and dried ($MgSO_4$), filtered and evaporated under reduced pressure to give the crude product. The crude product was analysed by ^{1}H NMR spectroscopy with DMF (40 µL, 0.5166 mmol) as an external standard. Compound **23** was prepared in 68% yield, a double substituted product **24** was prepared in 9% yield and there was no evidence of starting material **22**. Attempted purification by column chromatography (SIO_2, hexane →hexane:EtOAc, 10:1) gave an inseparable 88:12 mixture of **23** and **24** by ^{1}H NMR spectroscopy. The recorded spectroscopic data for **23** correlate with those reported in literature.[9]

23: ^{1}H NMR ($CDCl_3$, 400 MHz, 293 K): δ = 7.32-7.21 (m, 5H), 3.63 (s, 3H), 3.54 (t, *J* = 7.7 Hz, 1H), 2.08-1.93 (m, 1H), 1.79-1.68 (m, 1H), 1.30-1.18 (m, 2H), 0.89 (t, *J* = 7.3 Hz, 3H). ^{13}C NMR ($CDCl_3$, 101 MHz, 293 K): δ = 174.7, 139.4, 128.7, 128.0, 127.3, 52.0, 51.5, 35.7, 20.8, 13.9.

Synthesis of *N*-(*tert*-Butoxycarbonyl)-2-ethyl 2-phenylpyrrolidine-2-carboxylate 28

2-Phenylpyrrolidine 26

An oven-dried 10 mL Schlenk flask was charged with a solution of pyrrolidine **25** (82 mL, 1.0 mmol) and benzophenone **4** (219 mg, 1.2 mmol) in dry diethyl ether (2 mL) under a nitrogen atmosphere. The solution was cooled to –78 °C and, to this mixture, a $PhLi_{gel}$ block (2.5 mmol, prepared using 0.66 g of $C_{36}H_{74}$, 2.6 mL dry dibutyl ether and 1.32 mL PhLi (1.91 M in dibutyl ether), prepared according to general procedure F) was quickly added by removing the rubber septum while keeping the flask under a positive pressure of nitrogen. After the gel block was added, the flask was sealed with the rubber septum. As the gel did not break up immediately during the stirring, it was partly cut into smaller pieces using a long needle. The mixture was stirred at 0 °C for 30 min. The resulting mixture was stirred at –78 °C for 15 min and then allowed to warm to room temperature and stirred at room temperature for 2 hours. The reaction was quenched by the addition of MeOH (1 mL). The mixture was diluted with diethyl ether (10 mL) and filtered through filter paper to remove the solids (gelator). The filtrate was washed with water (3 x 30 mL). The organic layer was collected and dried ($MgSO_4$), filtered and evaporated under reduced pressure to give the crude product. Purification by column chromatography (SiO_2, EtOAc:Et_3N, 10:0.1 → EtOAc:MeOH:Et_3N, 10:1:0.1) gave 2-phenylpyrrolidine **26** (0.0546 g, 37%) as a yellow oil. The recorded spectroscopic data correlate with those reported in the literature.[10]

^{1}H NMR ($CDCl_3$, 400 MHz, 293 K): δ = 7.39-7.27 (m, 4H), 7.26-7.18 (m, 1H), 4.10 (t, *J* = 7.7 Hz, 1H), 3.24-3.15 (m1H), 3.04-2.95 (m, 1H), 2.23-2.13 (m, 1H), 2.09 (bs, 1H), 1.98-1.78 (m, 2H), 1.72-1.60 (m, 1H). ^{13}C NMR ($CDCl_3$, 101 MHz, 293 K): δ = 144.9, 128.5, 126.9, 126.6, 62.7, 47.1, 34.4, 25.7.

For comparison, the preparation of 2-phenylpyrrolidine **26** was carried out according to the original procedure[10] using PhLi solution: pyrrolidine **25** (0.41 mL, 5.0 mmol), benzophenone **4** (1.095 g, 6.0 mmol) and PhLi (6.6 mL, 12.5 mmol, 1.91 M in dibutyl ether). Purification by column chromatography (SiO_2, EtOAc:Et_3N, 10:0.1 → EtOAc:MeOH:Et_3N, 10:1:0.1) gave 2-phenylpyrrolidine **26** (0.370 g, 50%) as a yellow oil.

N-(*tert*-Butoxycarbonyl)-2-phenylpyrrolidine 27

A solution of 2-phenylpyrrolidine **26** (0.214 g, 1.452 mmol), Boc_2O (0.634 g, 2.904 mmol), 4-(dimethylamino)pyridine (0.194 g, 1.452 mmol) and Et_3N (0.22 mL, 1.5 mmol) in dry dichloromethane (20 mL) was stirred at room temperature under a nitrogen atmosphere for 16 hours. The mixture was diluted with diethyl ether (50 mL), washed with 1 M $HCl_{(aq)}$ (3 x 20 mL) and saturated aqueous $NaHCO_{3(aq)}$ (3 x 20 mL). The organic layer was dried ($MgSO_4$), filtered and evaporated under reduced pressure to give the crude product. Purification by column chromatography (SiO_2, hexane:diethyl ether, 5:1) gave the *N*-Boc 2-phenylpyrrolidine **27** (0.3355 g, 93%) as a colourless oil. The recorded spectroscopic data correlate with those reported in the literature.[11]

The product gives two sets of NMR signals, due to the presence of rotamers.

^1H NMR ($CDCl_3$, 400 MHz, 293 K): δ = 7.31-7.23 (m, 2H), 7.21-7.11 (m, 3H), 4.94 (br s, 0.3H), 4.74 (br s, 0.7H), 3.69-3.43 (m, 2H), 2.36-2.18 (br m, 1H), 1.97-1.74 (m, 3H), 1.44 (s, 3H), 1.16 (s, 6H). ^{13}C NMR ($CDCl_3$, 101 MHz, 293 K): δ = 154.7, 145.2, 144.2, 128.2, 126.6, 125.6, 79.2, 61.4, 60.8, 47.2, 36.1, 34.9, 28.6, 28.2, 23.5, 23.3.

N-(*tert*-Butoxycarbonyl)-2-ethyl 2-phenylpyrrolidine-2-carboxylate 28

An oven-dried 10 mL Schlenk flask was charged with *N*-Boc 2-phenylpyrrolidine **27** (0.075 g, 0.302 mmol) and dry THF (5 mL) and kept under a nitrogen atmosphere. The solution was cooled to 0 °C and, to this mixture, a *n*-$BuLi_{gel}$ block (prepared using 0.119 g of $C_{36}H_{74}$, 0.47 mL dry hexane and 0.28 mL *n*-BuLi (1.6 M in hexane), prepared according to general procedure F) was quickly added by removing the rubber septum while keeping the flask under a positive pressure of nitrogen. After the gel block was added, the flask was sealed with the rubber septum. As the gel did not break up immediately during the stirring, it was partly cut into smaller pieces using a long needle. The resulting mixture was stirred at 0 °C for 5 min. Then, ethyl chloroformate (60.5 µL, 0.635 mmol) was added dropwise. The mixture was stirred for at 0 °C for 10 min and then allowed to warm to room temperature. The reaction was quenched by the addition of sat. $NH_4Cl_{(aq)}$ (1 mL). The mixture was diluted with diethyl ether (10 mL) and filtered through a short pad of celite to remove the solids (gelator). The filtrate was washed with water (3 x 30 mL). The organic layer was collected and dried ($MgSO_4$), filtered and evaporated under reduced pressure to give the crude product. Purification by column chromatography (SiO_2, hexane:EtOAc, 4:1) gave disubstituted *N*-Boc pyrrolidine **28** (0.0752 g, 78%) as a colourless oil. The recorded spectroscopic data correlate with those reported in the literature.[12]

The product gives two sets of NMR signals, due to the presence of rotamers.

^1H NMR ($CDCl_3$, 400 MHz, 293 K): δ = 7.38-7.19 (m, 5H), 4.36-4.07 (m, 2H), 3.76-3.55 (m, 2H), 2.64-2.50 (m, 1H), 2.29-2.21 (m, 1H), 1.96-1.81 (m, 1H), 1.75-1.60 (m, 1H), 1.48 (s, 3H), 1.33-1.17 (m, 9H). ^{13}C NMR ($CDCl_3$, 101 MHz, 293 K): δ = 172.8, 154.0, 140.7, 127.6, 127.4, 127.3, 127.2, 127.0, 80.3, 79.9, 71.6, 61.5, 48.0, 43.7, 42.0, 28.5, 28.1, 28.0, 23.6, 22.7, 14.3.

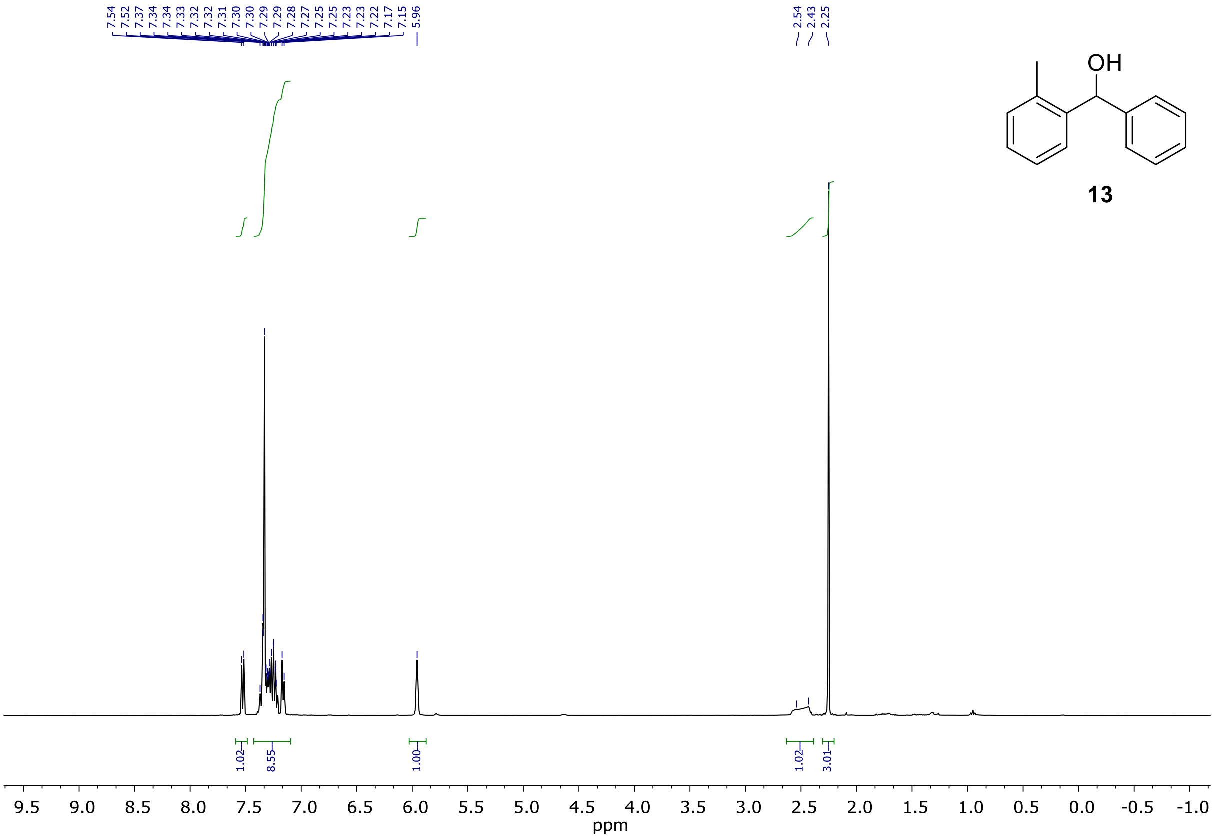

Figure S59. ^{1}H NMR spectrum of **13** ($CDCl_3$, 298 K, 400 MHz).

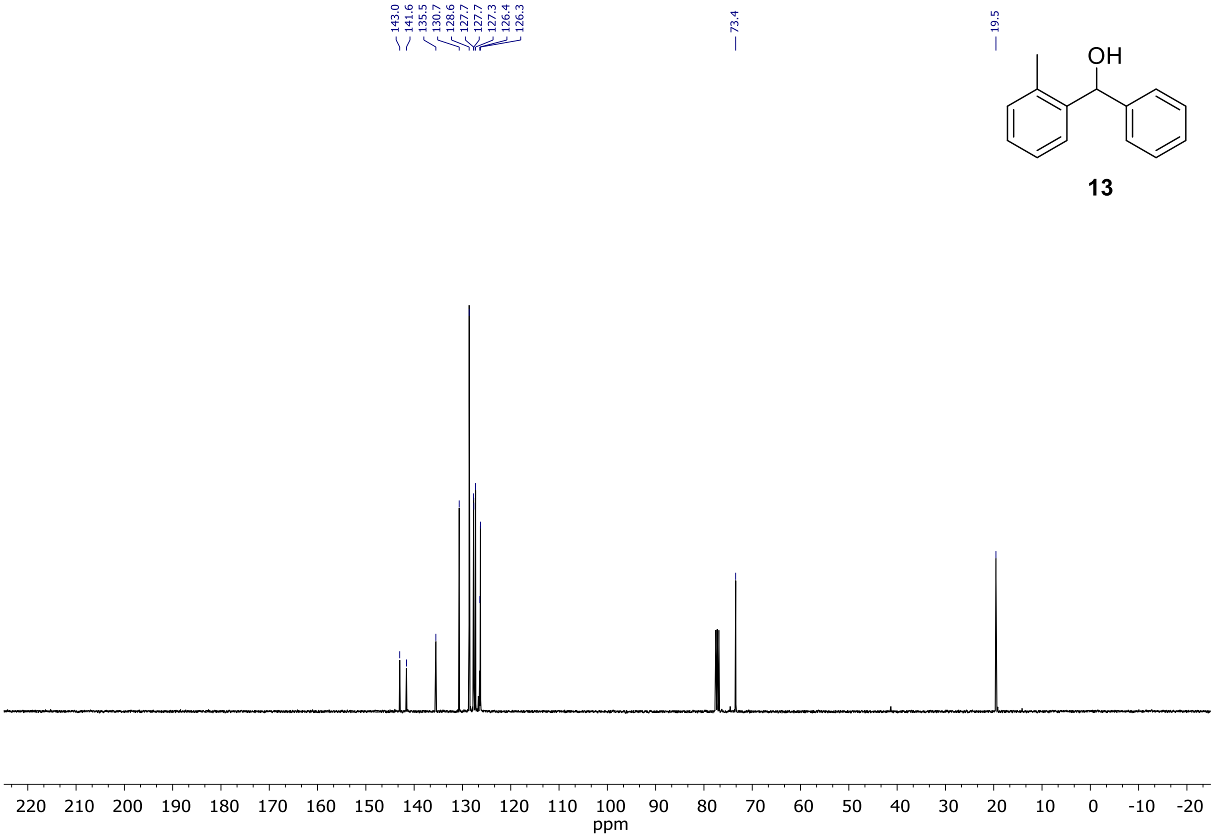

Figure S60. ^{13}C NMR spectrum of **13** ($CDCl_3$, 298 K, 101 MHz).

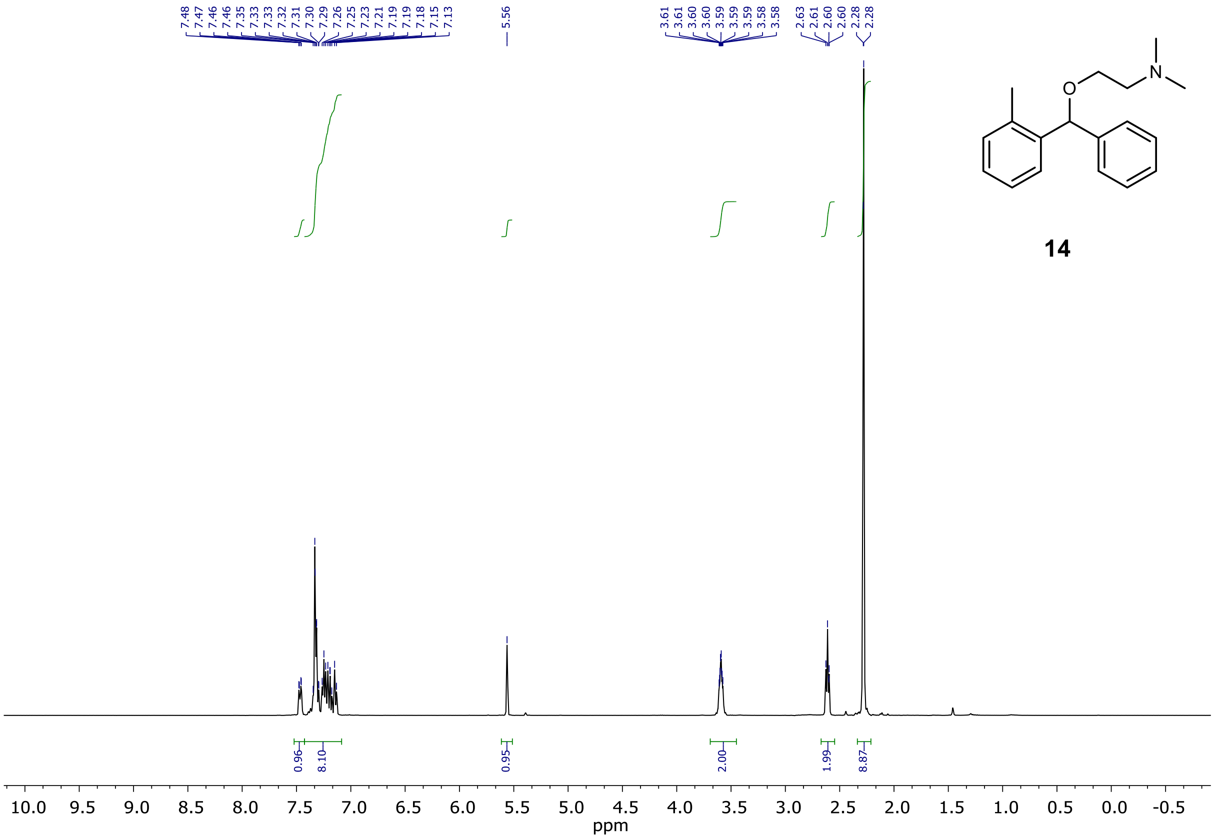

Figure S61. ^{1}H NMR spectrum of **14** ($CDCl_3$, 298 K, 400 MHz).

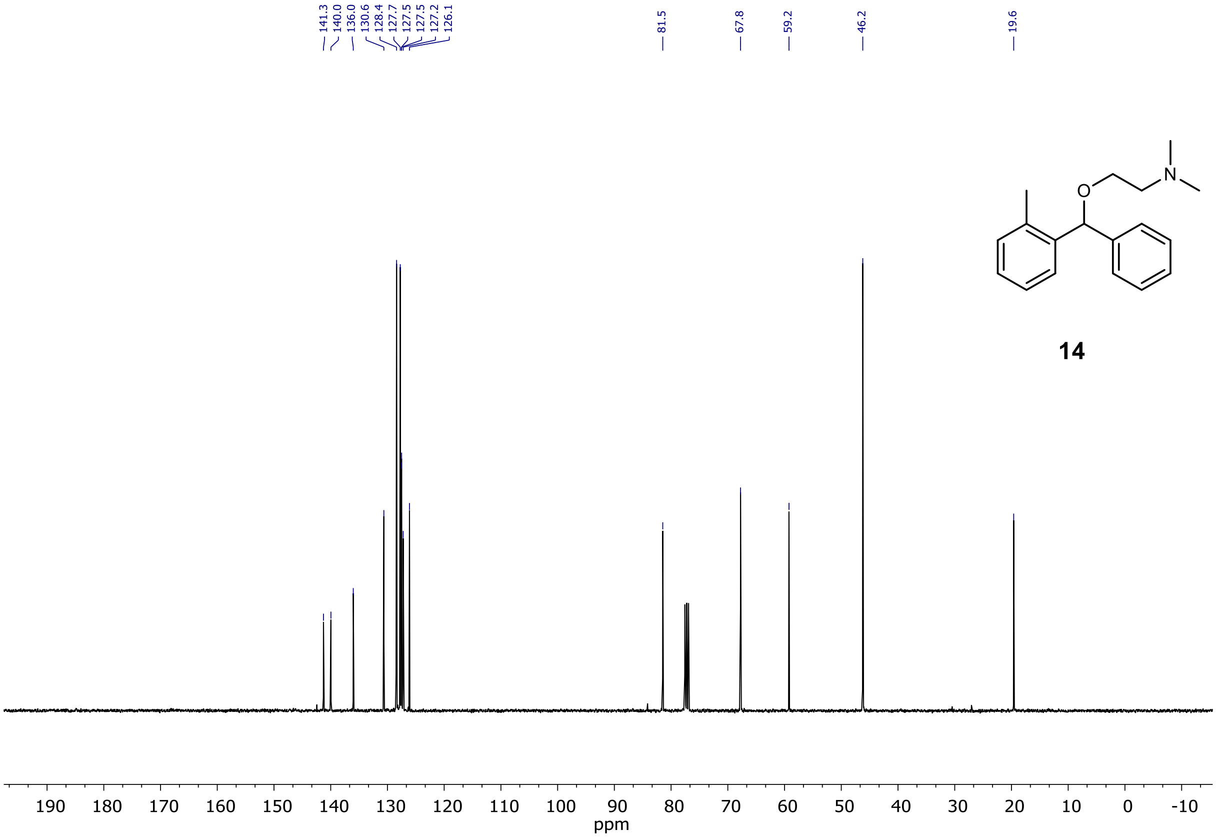

Figure S62. ^{13}C NMR spectrum of **14** ($CDCl_3$, 298 K, 101 MHz).

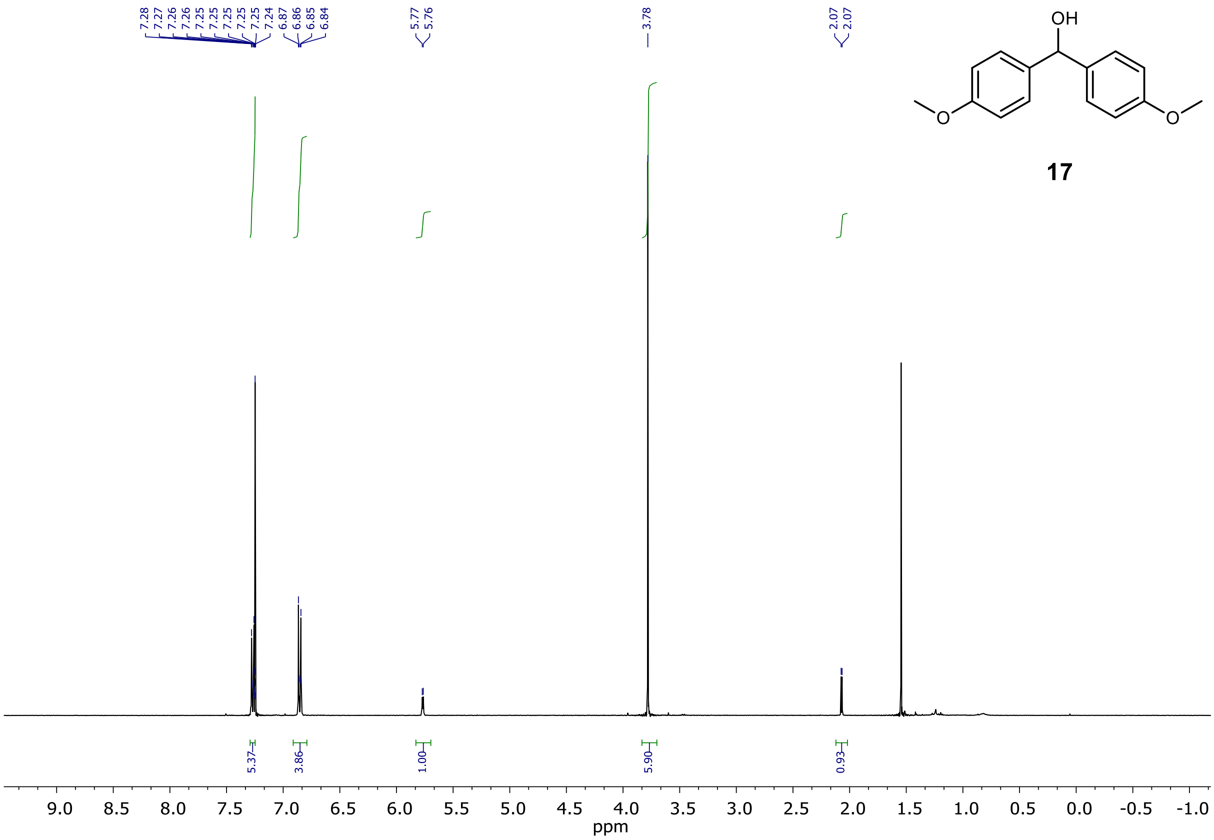

Figure S63. ^{1}H NMR spectrum of **17** ($CDCl_3$, 298 K, 400 MHz).

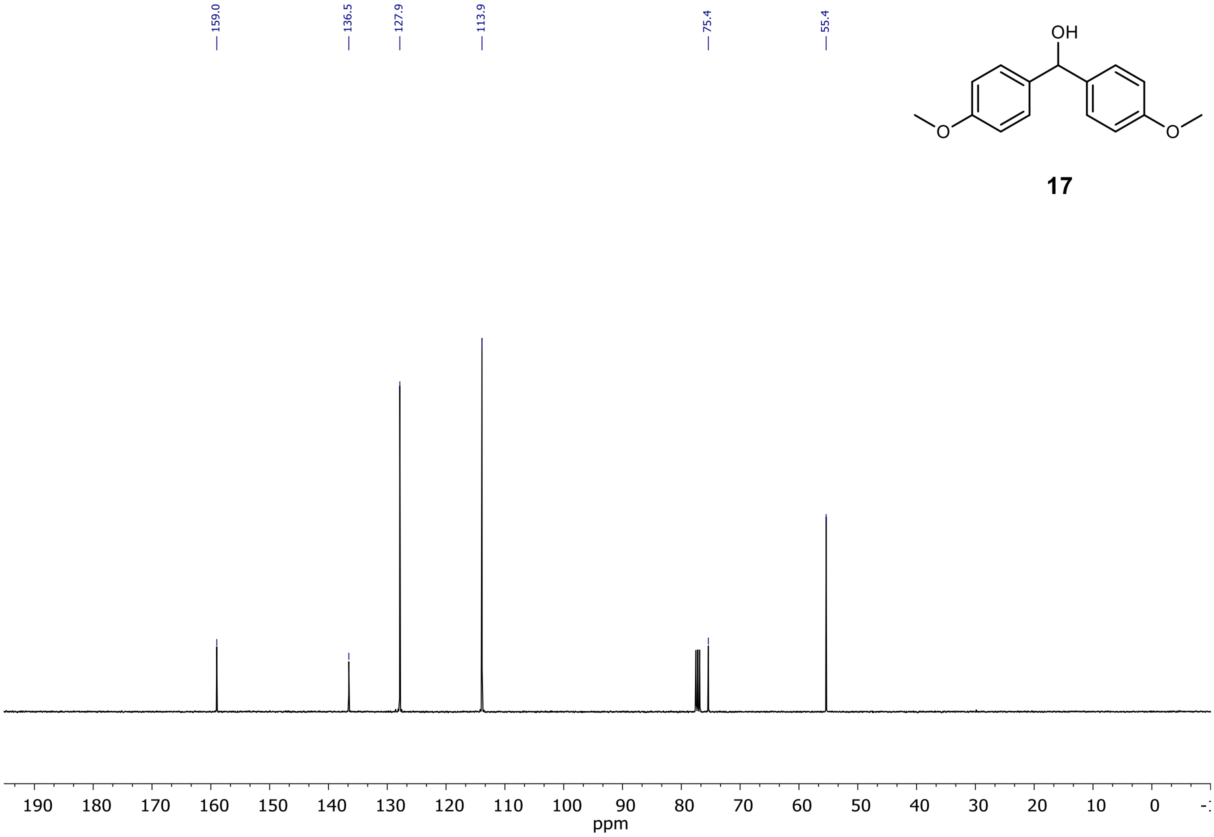

Figure S64. ^{13}C NMR spectrum of **17** ($CDCl_3$, 298 K, 101 MHz).

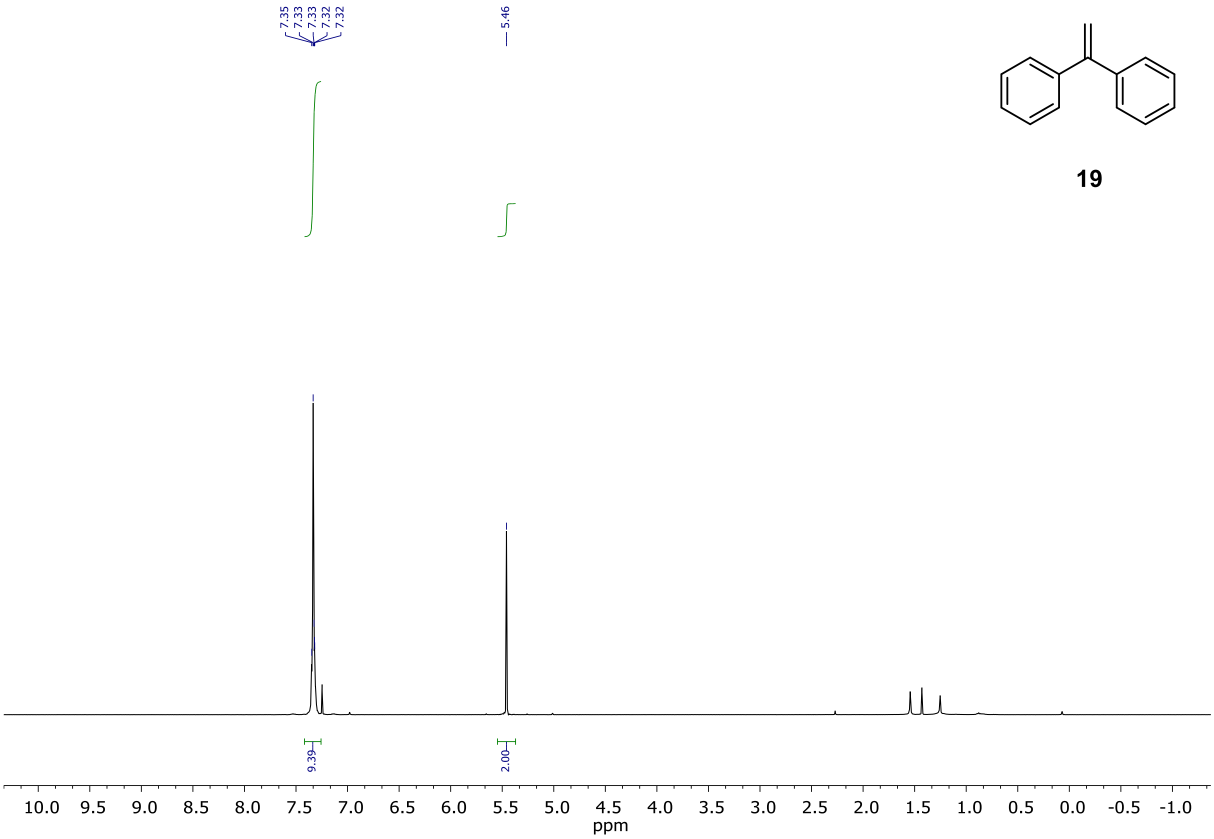

Figure S65. ^{1}H NMR spectrum of **19** ($CDCl_3$, 298 K, 400 MHz).

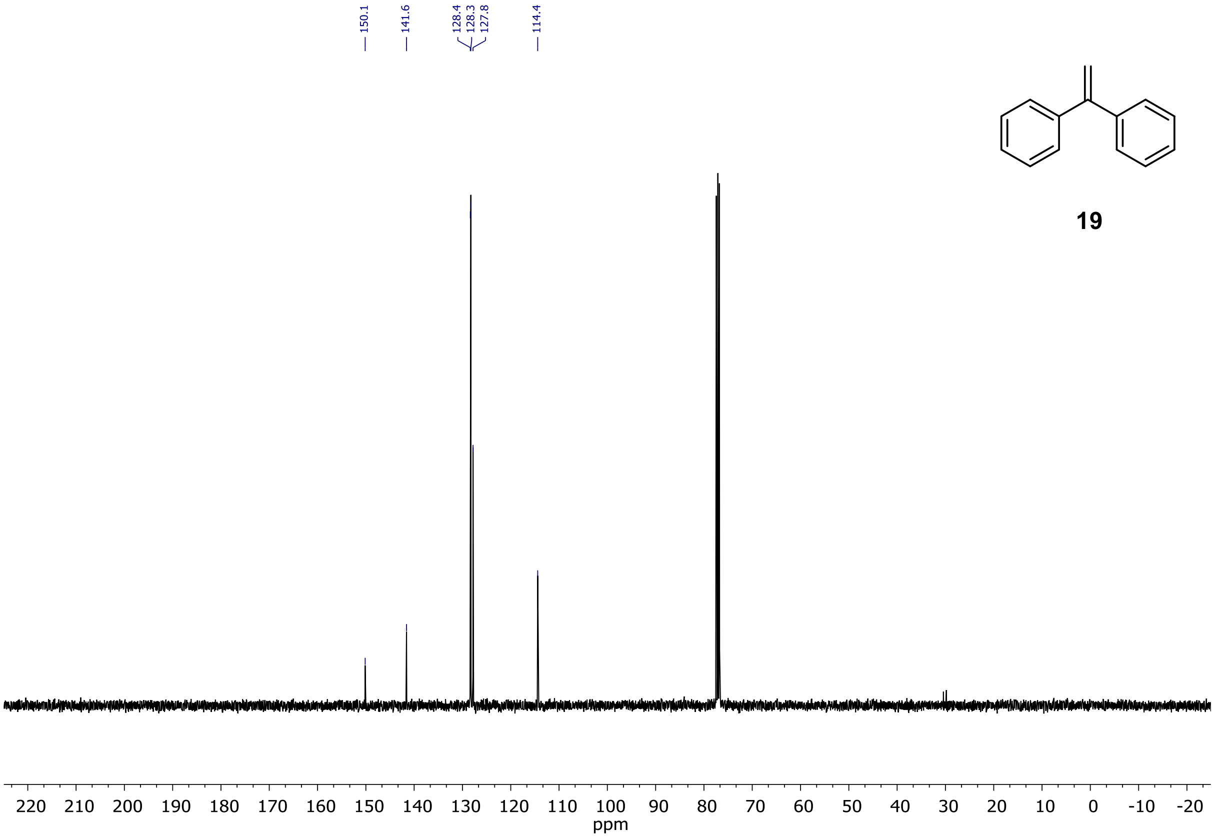

Figure S66. ^{13}C NMR spectrum of **19** ($CDCl_3$, 298 K, 101 MHz).

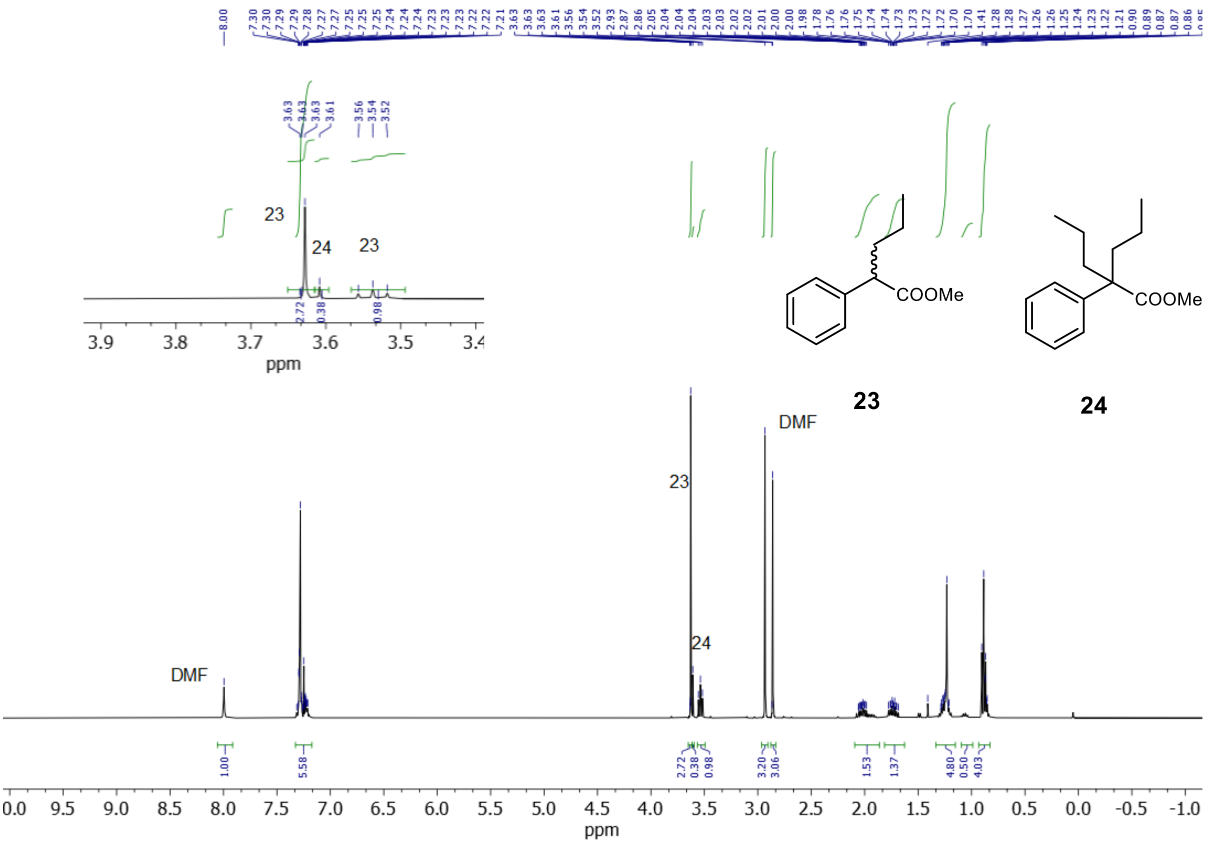

Figure S67. ^{1}H NMR spectrum of the crude product containing **23** and **24** ($CDCl_3$, 298 K, 400 MHz) with DMF (0.5166 mmol) as an external standard.

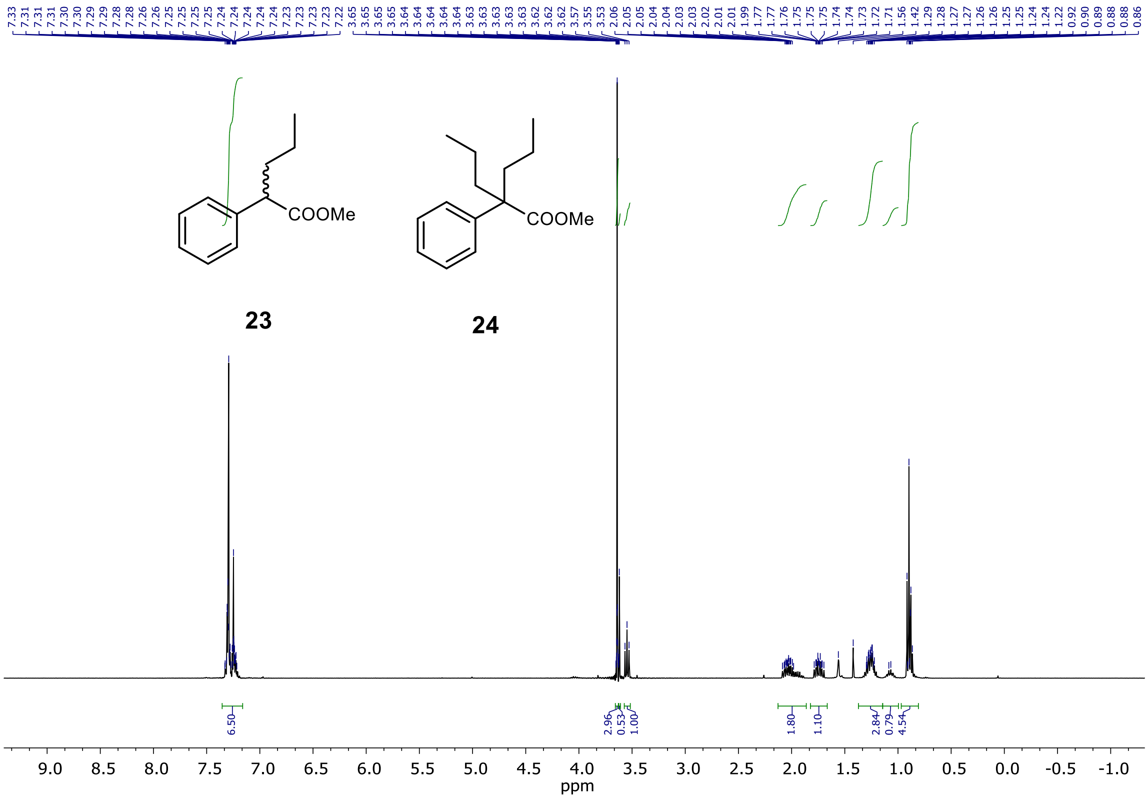

Figure S68. ^{1}H NMR spectrum of the inseparable 88:12 mixture of **23** and **24** obtained after column chromatography ($CDCl_3$, 298 K, 400 MHz).

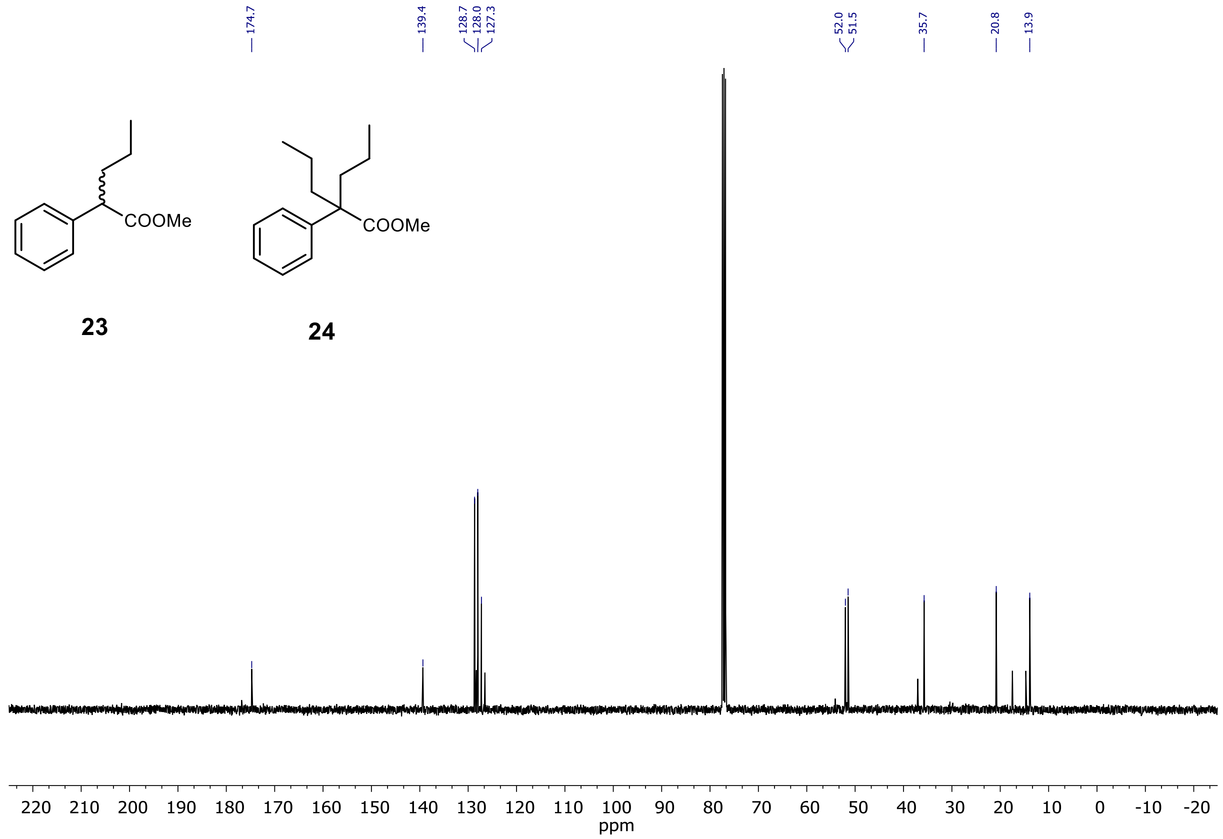

Figure S69. ^{13}C NMR spectrum of the inseparable 88:12 mixture of **23** and **24** obtained after column chromatography ($CDCl_3$, 298 K, 101 MHz)- annotated signals belong to **23.**

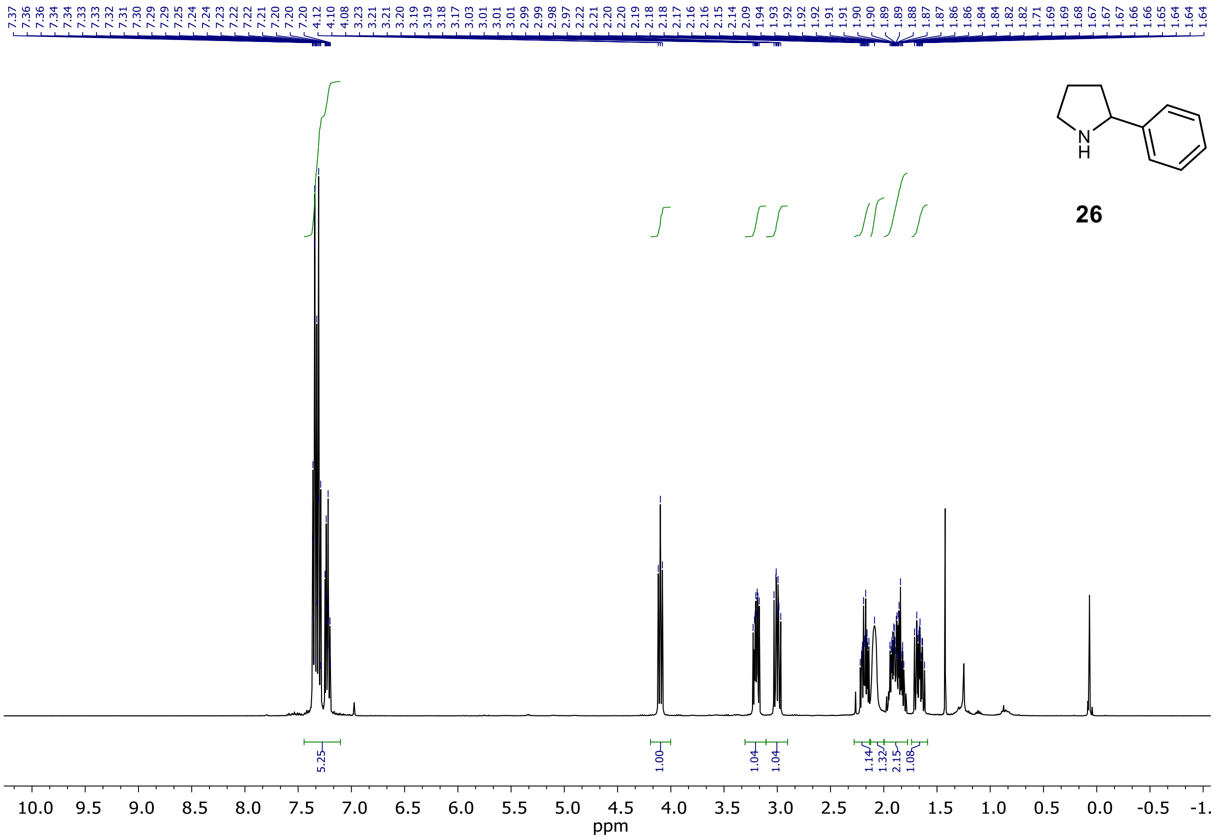

Figure S70. ^{1}H NMR spectrum of **26** ($CDCl_3$, 298 K, 400 MHz).

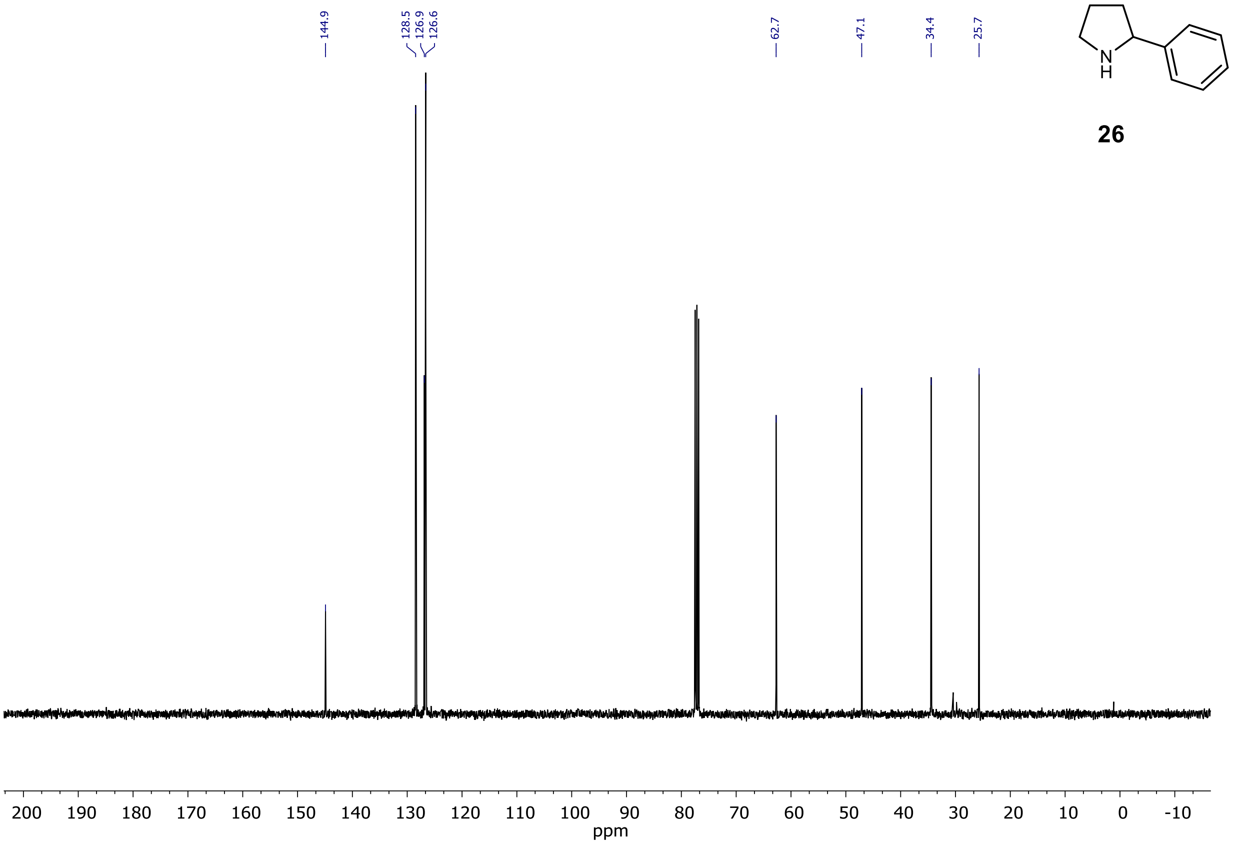

Figure S71. ^{13}C NMR spectrum of **26** ($CDCl_3$, 298 K, 101 MHz).

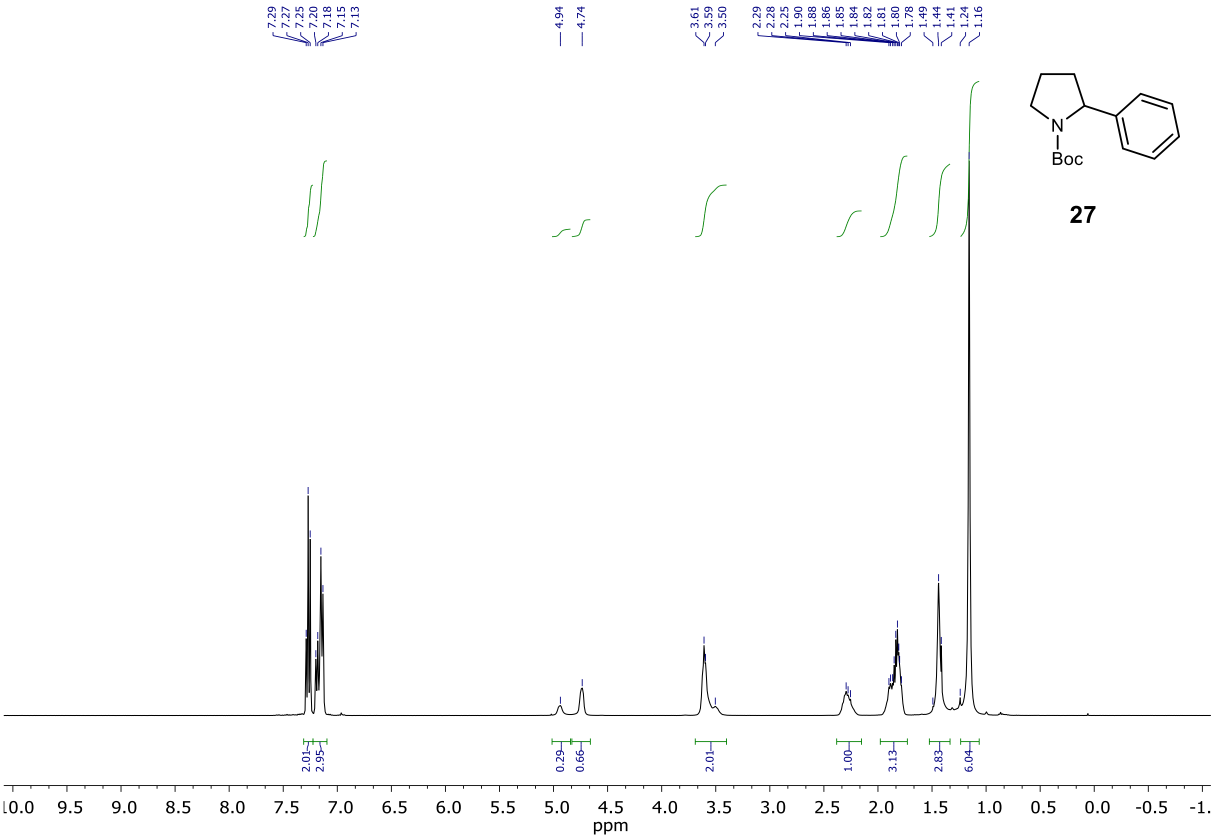

27

Figure S72. ^{1}H NMR spectrum of **27** ($CDCl_3$, 298 K, 400 MHz).

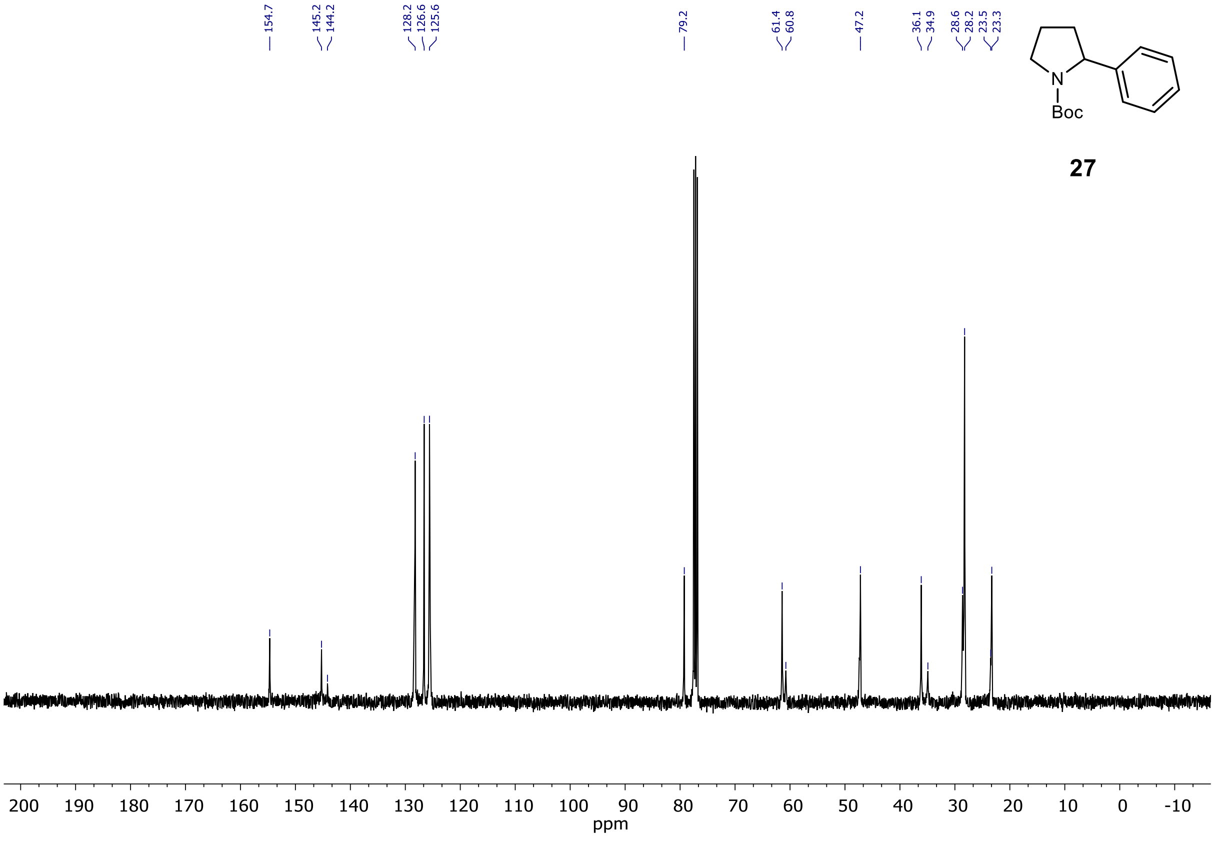

27

Figure S73. ^{13}C NMR spectrum of **27** ($CDCl_3$, 298 K, 101 MHz).

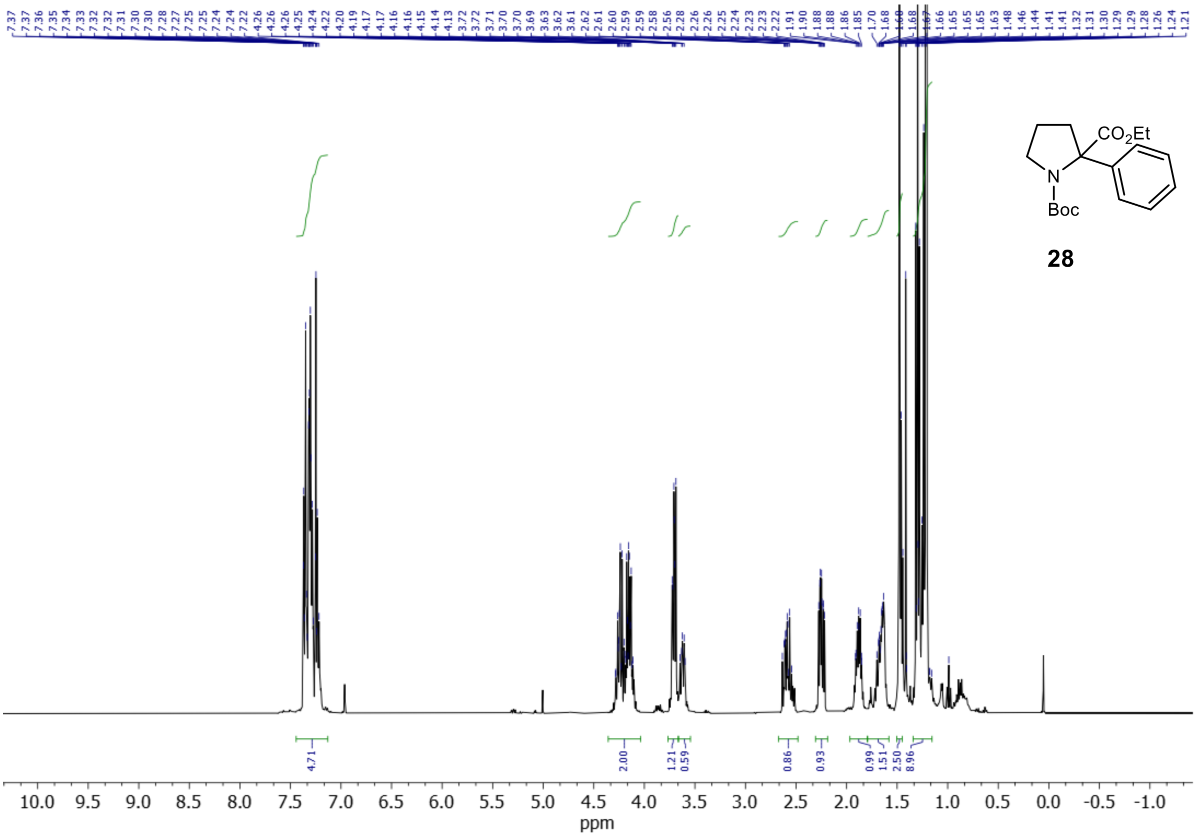

Figure S74. ^{1}H NMR spectrum of **28** ($CDCl_3$, 298 K, 400 MHz).

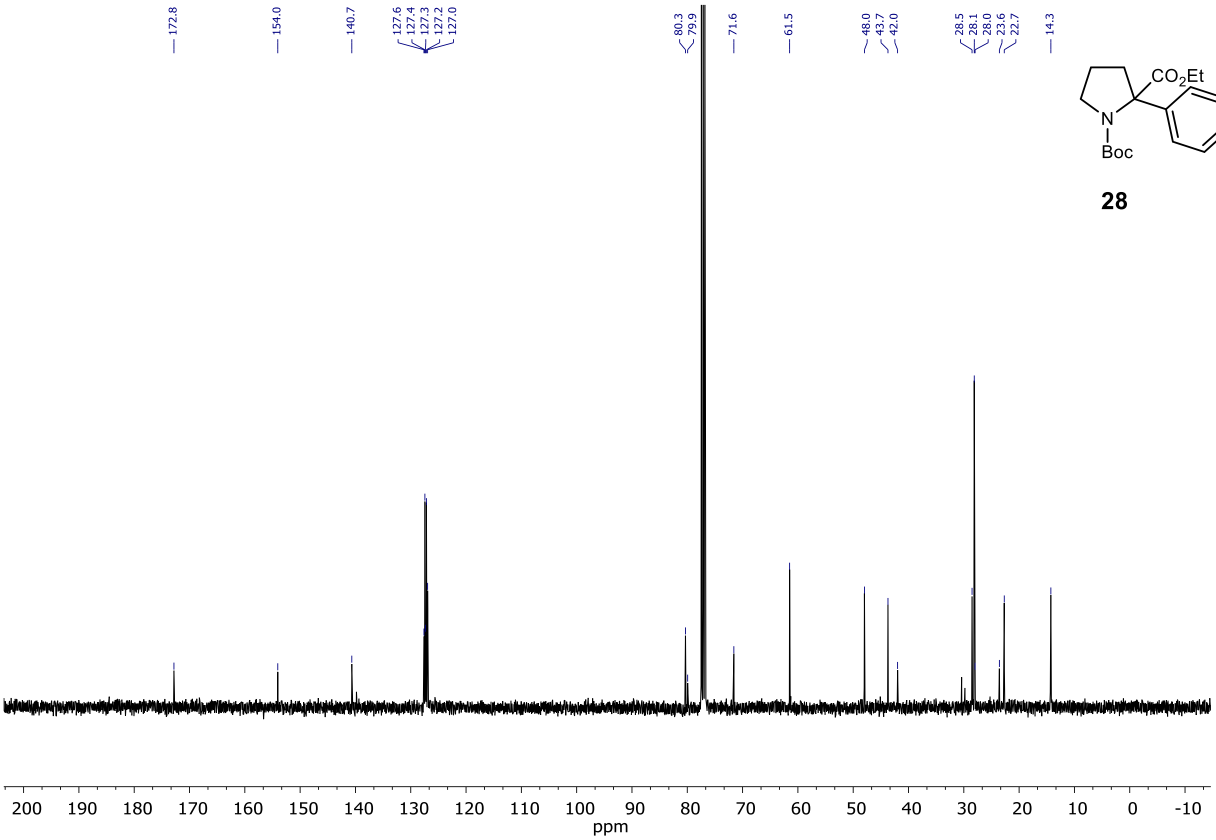

Figure S75. ^{13}C NMR spectrum of **28** ($CDCl_3$, 298 K, 101 MHz).

6. Preparation of *s*-BuLi Gel in a Vial and Subsequent Reactions (Results in Fig. 4)

General procedure I: preparation of *s*-BuLi$_{gel}$ in a vial and determination of stability by titration

A 5 mL vial was dried in the oven, evacuated and allowed to cool under a nitrogen atmosphere. The vial was charged with gelator $C_{36}H_{74}$, sealed with a rubber septum and flushed with nitrogen three times. Anhydrous and degassed hexane (2.0 mL) was added followed by the addition of *s*-BuLi (1.15 M in hexanes, 1.0 mL, 1.15 mmol). The vial (kept under a nitrogen atmosphere using a balloon) was carefully heated until all of the gelator was dissolved. Then, the vial was immediately placed in iced water for 1 min until the organogel formed. The septum was removed from the vial and the organogel was exposed to air for a specified amount of time (see Table S11). The concentration of *s*-BuLi present in the gel was determined by titration experiments using (+)-menthol in THF with 2,2'-bipyridine as an indicator.[4]

Table S11 Screening of *s*-BuLi$_{gel}$ stability under ambient conditions

Entry	Gelator loading (% w/v)	Exposure	Concentration (molL^{-1})	% left of *s*-BuLi
1	0 (solution)	No exposure	0.380	100%
2	0 (solution)	5 min, air	0.140	37%
3	0 (solution)	10 min, air	0.090	24%
4	2.7	Gel did notform	N.D	-
5	5.0	Gel did not form	N.D	-
6	10	30 min, air	0.102	27%
7	15	30 min, air	0.187	49%
8	20	No exposure	0.370	97%
9	20	5 min, air	0.297	78%
10	20	10 min, air	0.277	73%
11	20	30 min, air	0.200	53%
12	22.5	30 min, air	0.230	61%

Stock solution of *s*-BuLi was 1.15 molL^{-1}

Reaction of *s*-BuLi$_{gel}$ with benzophenone 4 using an organolithium gel in a vial (Fig. 4G)

s-BuLi$_{gel}$ (1.2 mmol, 2.0 equiv, 20.0% wt/vol) in a vial was prepared according to general procedure I. The organolithium gel was exposed to air for 10 min by removing the rubber septum. At room temperature, a solution of benzophenone (0.11 g, 0.6 mmol, 1.0 equiv) in dry hexane (1 mL) was added on top of the gel and the vial was closed with a screw cap. The mixture was vigorously stirred for 5 s before the reaction was quenched by the addition of water (0.5 mL). The solids in the reaction mixture were removed by filtration using a glass funnel and filter paper. This procedure removed most of the $C_{36}H_{74}$ gelator. The reaction vial and filter paper with the gelator were washed with additional hexane (3 × 3 mL) and the combined filtrates were dried ($MgSO_4$) and evaporated under reduced pressure to give the crude product. Purification by column chromatography (SiO_2, hexane → hexane:EtOAc 49:1) gave 2-methyl-1,1-diphenyl-1-butanol **29** (0.06 g, 42%) as a colourless oil. Compound **29** has been previously reported and the spectroscopic data were in agreement.[13]

^{1}H NMR ($CDCl_3$, 400 MHz, 293 K): δ 7.53−7.48 (m, 4H), 7.33−7.27 (m, 4H), 7.21−7.14 (m, 2H), 2.61−2.50 (m, 1H), 2.05 (br s, 1H), 1.58−1.44 (m, 1H), 1.13−1.01 (m, 1H), 0.96 (t, *J* = 7.5 Hz, 3H), 0.90 (d, *J* = 6.5 Hz, 3H); ^{13}C NMR ($CDCl_3$, 101 MHz, 293 K) δ 146.9, 128.3, 128.2, 126.5, 126.4, 125.84, 125.76, 81.3, 42.4, 24.3, 13.6, 12.7.

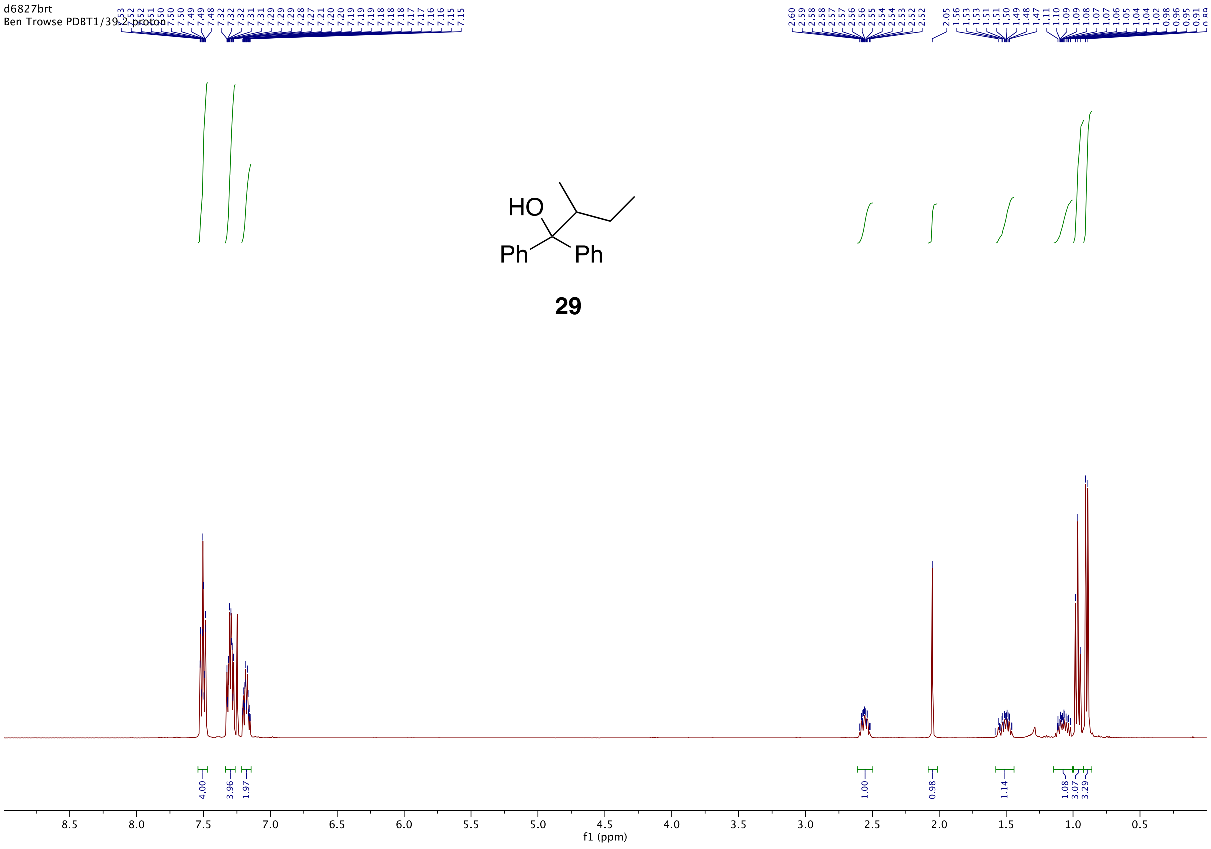

Figure S76. ^{1}H NMR spectrum of **29** ($CDCl_3$, 298 K, 400 MHz).

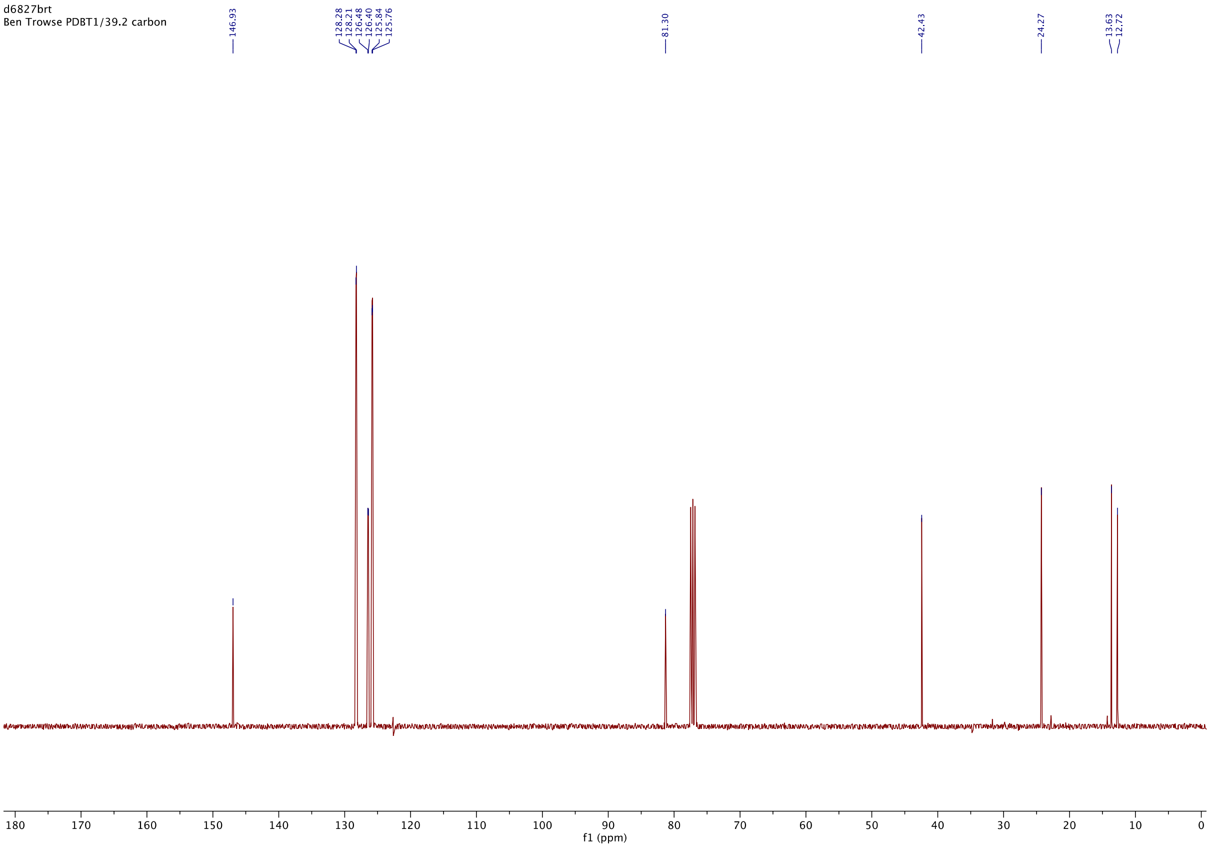

Figure S77. ^{13}C NMR spectrum of **29** ($CDCl_3$, 298 K, 400 MHz).

Reaction of *s*-BuLi$_{gel}$ with *N*-benzylideneaniline 6 using an organolithium gel in a vial (Fig. 4G)

s-BuLi$_{gel}$ (1.2 mmol, 2.0 equiv, 20.0% wt/vol) in a vial was prepared according to general procedure I. The organolithium gel was exposed to air for 10 min by removing the rubber septum. At room temperature, a solution of *N*-benzylideneaniline (0.11 g, 0.6 mmol, 1.0 equiv) in dry hexane (1 mL) was added on top of the gel and the vial was closed with a screw cap. The mixture was vigorously stirred for 5 s before the reaction was quenched by the addition of water (0.5 mL). The solids in the reaction mixture were removed by filtration using a glass funnel and filter paper. This procedure removed most of the $C_{36}H_{74}$ gelator. The reaction vial and filter paper with the gelator were washed with additional hexane (3 × 3 mL) and the combined filtrates were dried ($MgSO_4$) and evaporated under reduced pressure to give the crude product. Purification by column chromatography (SiO_2, hexane → hexane:EtOAc 49:1) gave *N*-phenyl-(1-phenyl-2-methylbutyl)amine **30** (0.14 g, 98%) as a yellow oil. Compound **30** has been previously reported and the spectroscopic data were in agreement.[13]

^{1}H NMR ($CDCl_3$, 400 MHz, 293 K) (55:45 mixture of diastereomers): δ = 7.32−7.28 (m, 4H), 7.24−7.18 (m, 1H), 7.11−7.03 (m, 2H), 6.65−6.58 (m, 1H), 6.54−6.46 (m, 2H), 4.31 (d, *J* = 5.0 Hz, 0.45H), 4.22 (d, *J* = 6.0 Hz, 0.55H), 4.10 (br s, 1H), 1.87−1.76 (m, 1H), 1.67−1.45 (m, 1.45H), 1.25−1.14 (m, 0.55H), 0.98−0.84 (m, 6H); ^{13}C NMR ($CDCl_3$, 101 MHz, 293 K) (mixture of diastereomers) δ 147.9, 143.1, 142.5, 129.20, 129.19, 128.4, 128.3, 127.5, 127.1, 126.9, 126.8, 117.10, 117.08, 113.3, 62.7, 61.6, 42.0, 41.6, 27.0, 25.5, 16.2, 14.5, 12.2, 11.9.

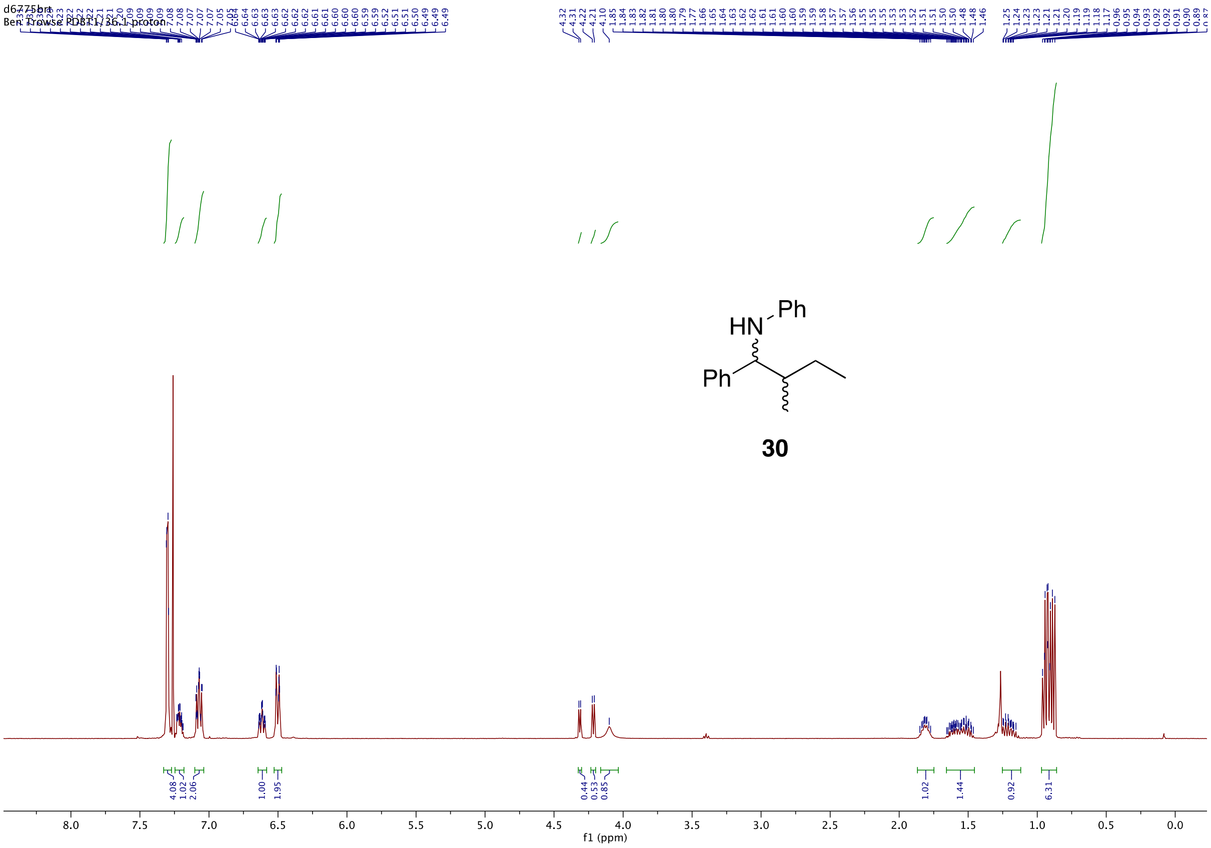

Figure S78. ^{1}H NMR spectrum of **30** ($CDCl_3$, 298 K, 400 MHz).

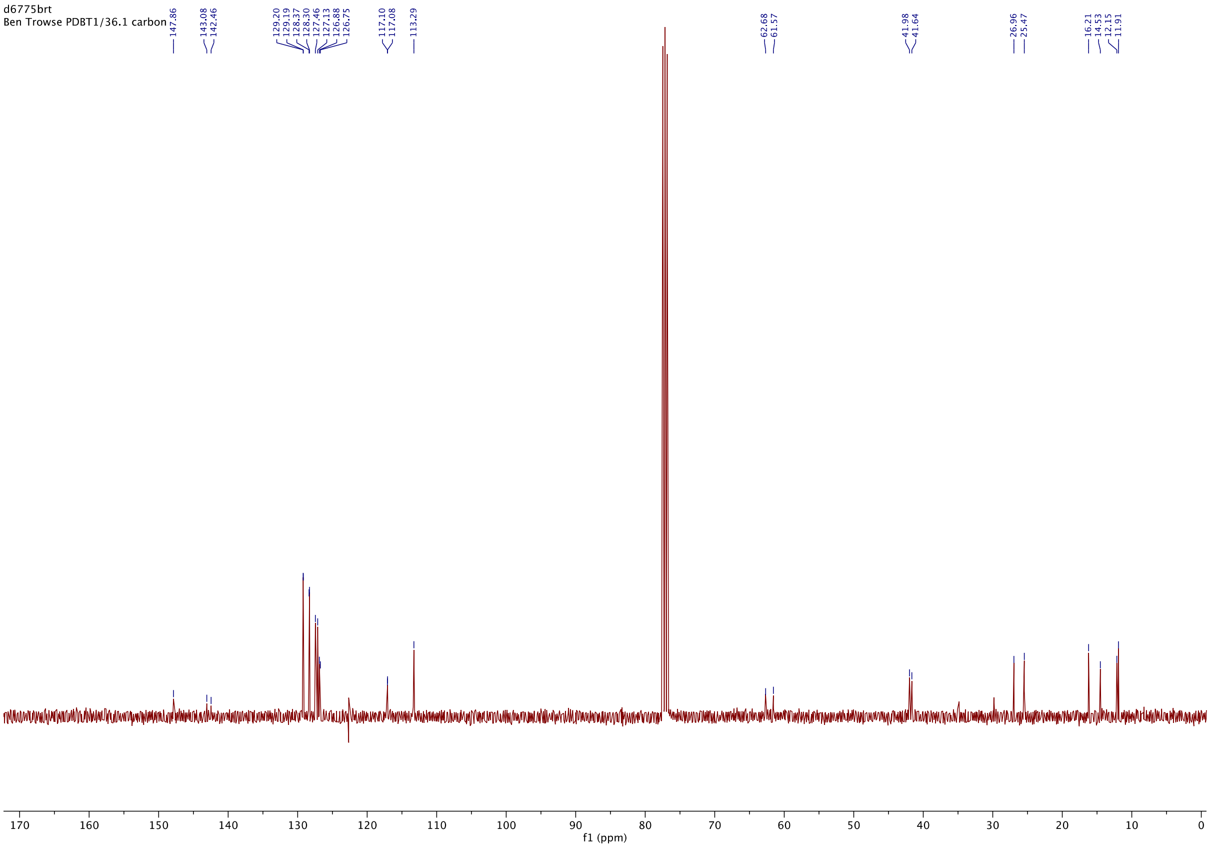

Figure S79. ^{13}C NMR spectrum of **30** ($CDCl_3$, 298 K, 400 MHz).

7. Preparation of *n*-BuLi/TMEDA Gel in a Vial and Subsequent Reactions (Results in Fig. 4)

General procedure J: preparation of *n*-BuLi/TMEDA$_{gel}$ in a vial (Fig. 4H)

A 5 mL vial was dried in the oven and allowed to cool under a nitrogen atmosphere. The vial was charged with gelator $C_{36}H_{74}$ (0.50 g, 1.0 mmol, 25.0% wt/vol), sealed with a rubber septum and flushed with nitrogen three times. TMEDA (0.19 g, 0.24 mL, 1.6 mmol) and anhydrous and degassed hexane (0.76 mL) was added followed by the addition of *n*-BuLi (1.6 M in hexane, 1.0 mL, 1.6 mmol). The vial (kept under a nitrogen atmosphere using a balloon) was carefully heated until all of the gelator was dissolved. Then, the vial was immediately placed in iced water for 1 min until the organogel formed.

It was necessary to vary the concentration of the gelator in order to obtain a stable gel under air. The results obtained are shown in Table S12. It was found that 25.0% wt/vol of gelator was necessary in order to obtain a gel that was stable to air for 30 min

Table S12 Screening of *n*-BuLi/TMEDA$_{gel}$ formation under ambient conditions

Entry	Concentration of $C_{36}H_{74}$ gelator (% wt/vol)	Stable gel under air [Yes/No]
1	2.7	No
2	5.0	No
3	10.0	No
4	15.0	No
5	20.0	No
6	25.0	Yes

ortho-Lithiation of anisole 31 using *n*-BuLi/TMEDA$_{gel}$ and trapping with Me_3SiCl using an organolithium gel in a vial (Fig. 4H)

n-BuLi/TMEDA$_{gel}$ (1.6 mmol, 1.3 equiv, 25.0% wt/vol) in a vial was prepared according to general procedure J. The organolithium gel was exposed to air for 30 min by removing the rubber septum. At room temperature, anisole **31** (0.13 g, 1.2 mmol, 1.0 equiv) in dry Et_2O (0.5 mL) was added on top of the gel and the vial was closed with a screw cap. The mixture was vigorously stirred for 30 min before the addition of a solution of Me_3SiCl (0.26 g, 2.4 mmol, 2.0 equiv) in dry Et_2O (0.5 mL) and stirred at room temperature for a further 15 min. The reaction was quenched by the addition of saturated $NH_4Cl_{(aq)}$ (1 mL). The solids in the reaction mixture were removed by filtration using a glass funnel and filter paper. This procedure removed most of the $C_{36}H_{74}$ gelator. The reaction vial and filter paper with the gelator were washed with additional hexane (3 × 3 mL) and the combined filtrates were dried ($MgSO_4$) and evaporated under reduced pressure to give the crude product. Purification by column chromatography (SiO_2, hexane → hexane:EtOAc 99:1) gave (2-methoxy-phenyl)-trimethylsilane **32** (0.13 g, 60%) as a colourless oil. Compound **32** has been previously reported and the spectroscopic data were in agreement.[14]

^{1}H NMR ($CDCl_3$, 400 MHz, 293 K): δ = 7.41–7.33 (m, 2H), 6.96 (t, *J* = 7.0 Hz, 1H), 6.84 (d, *J* = 8.0 Hz, 1H), 3.81 (s, 3H), 0.28 (s, 9H); ^{13}C NMR ($CDCl_3$, 101 MHz, 293 K): δ = 164.5, 135.1, 130.8, 128.1, 120.5, 109.7, 55.1, −0.8.

For comparison, the preparation of (2-methoxy-phenyl)-trimethylsilane **32** was carried out according to the original procedure:[41] anisole **31** (0.11 g, 1.0 mmol), TMEDA (0.15 g, 0.2 mL, 1.3 mmol) and *n*-BuLi (0.81 mL, 1.6 M solution in hexane, 1.3 mmol) in dry Et_2O (5 mL) at room temperature for 30 min then Me_3SiCl (0.22 g, 2.0 mmol). Purification by column chromatography (SiO_2, hexane → hexane:EtOAc 99:1) gave (2-methoxy-phenyl)-trimethylsilane **32** (0.11 g, 61%) as a colourless oil.

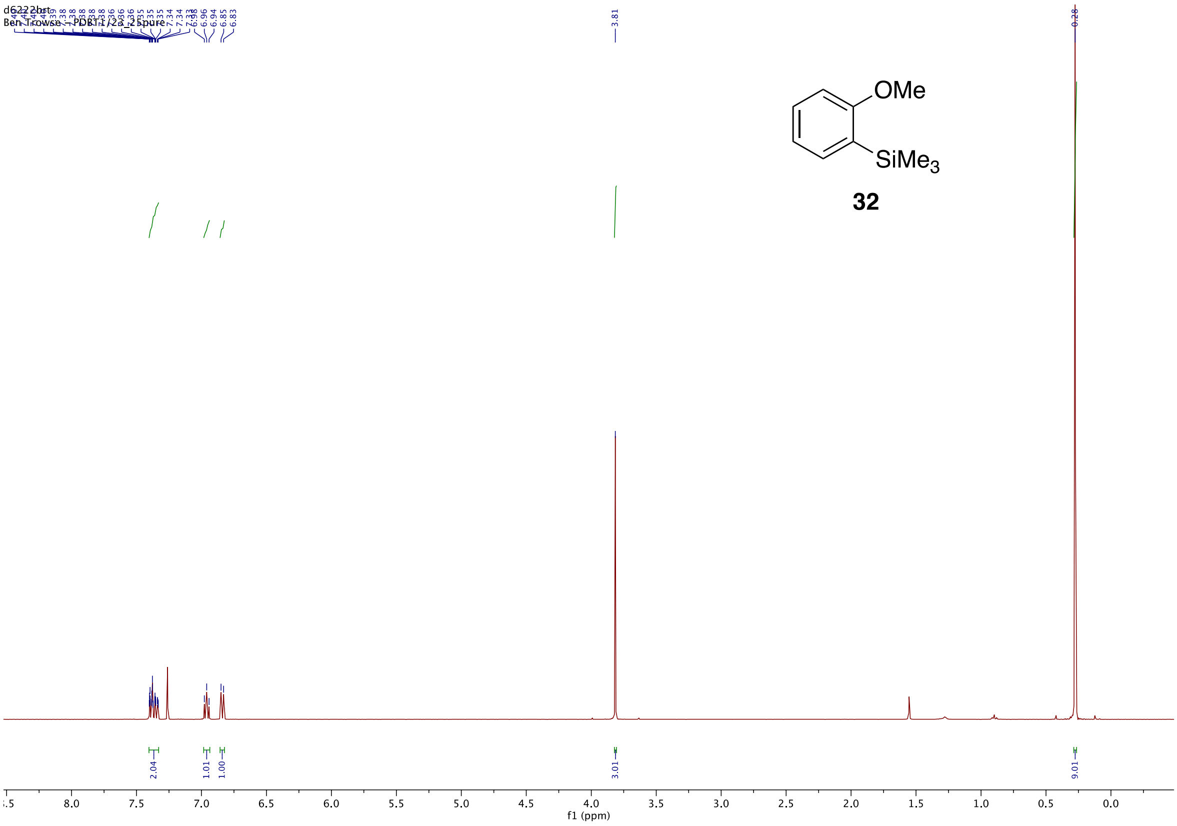

Figure S80. ^{1}H NMR spectrum of **32** ($CDCl_3$, 298 K, 400 MHz).

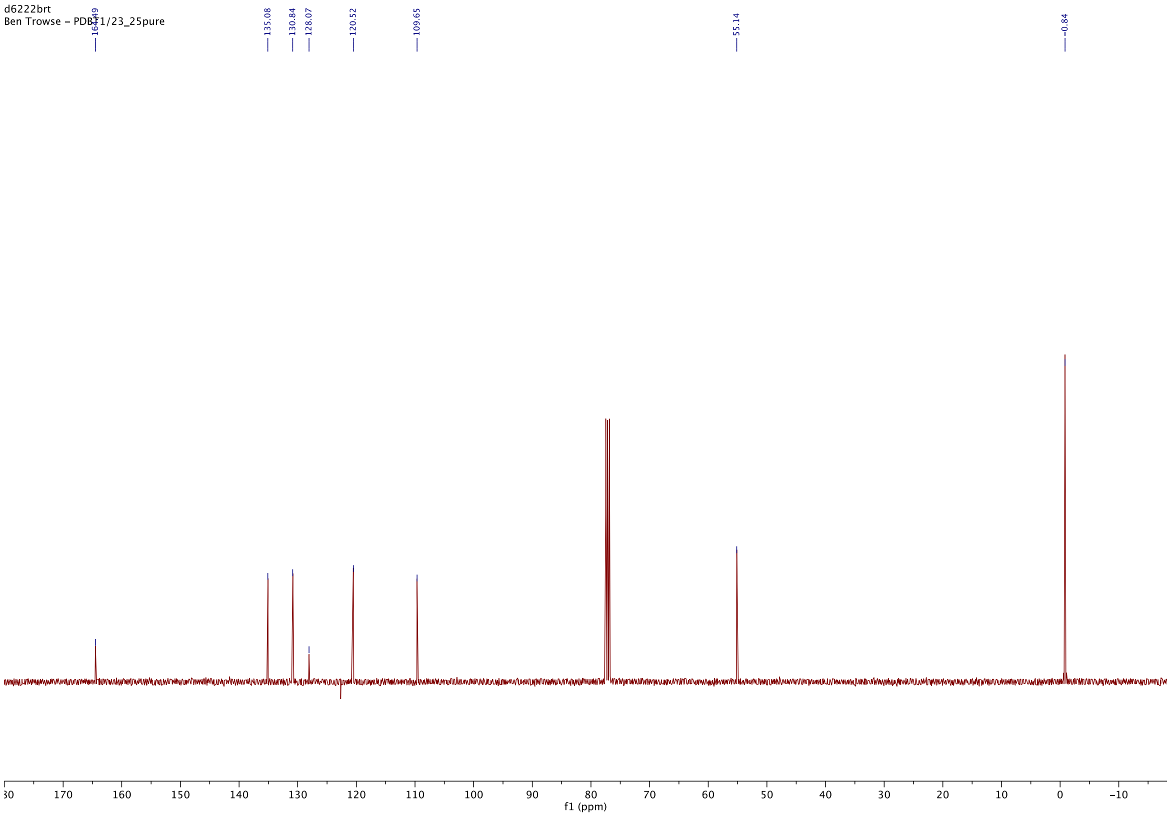

Figure S81. ^{13}C NMR spectrum of **32** ($CDCl_3$, 298 K, 400 MHz).

8. Preparation of vinylMgBr$_{gel}$ and PhMgCl$_{gel}$ Blocks and Subsequent Reactions (Results in Fig. 4)

General procedure K: preparation of robust vinylMgBr$_{gel}$ block

A 5 mL vial was dried in the oven and allowed to cool under a nitrogen atmosphere. The vial was charged with the gelator $C_{36}H_{74}$ (143 mg, 0.28 mmol, 10% wt/vol), sealed with a rubber septum and flushed with nitrogen via a needle for 5 min. Then, vinylmagnesium bromide (1.43 mL, 0.7 M in THF, 1.0 mmol) was added through the septum. The vial (kept under a nitrogen atmosphere using a balloon) was carefully heated until all of the gelator had dissolved. The hot hydrosol was quickly transferred under a nitrogen atmosphere via a needle into a 2 mL syringe (previously flushed with nitrogen and pre-heated in the oven). The syringe was immediately placed in iced water for 1 min until the organogel formed. The organomagnesium gel was kept in the syringe under a nitrogen atmosphere prior to use. In order to use the organomagnesium gel, the upper part of the syringe was carefully cut with scissors and the gel block was removed.

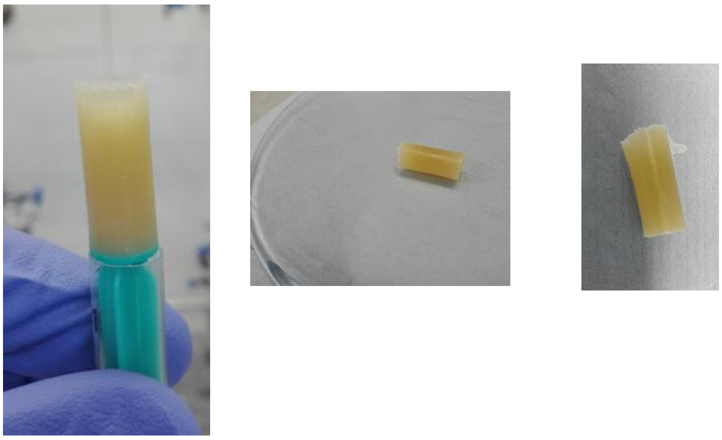

Figure S82. Images of vinylMgBr gel block.

Reaction of a vinylMgBr$_{gel}$ block with 2′-methoxyacetophenone 1 (Fig. 4l)

A vinylMgBr$_{gel}$ block (1.0 mmol) was prepared according to general procedure K. The organomagnesium gel was exposed to air by placing it on a petri dish in the fumehood for 5 min. The gel block was carefully placed in a 10 mL round-bottom flask containing 2′-methoxyacetophenone **1** (68.9 µL, 0.5 mmol) in dry THF (3 mL)at room temperature and under air. The mixture was vigorously stirred for 5 s before the reaction was quenched by the addition of water (0.5 mL). The solids in the reaction mixture were removed by filtration using a glass funnel and filter paper. This procedure removed most of the $C_{36}H_{74}$ gelator. The reaction vial and filter paper with the gelator were washed with additional dibutyl ether (3 x 2 mL) and the combined filtrates were dried ($MgSO_4$) and evaporated under reduced pressure to give the crude product. The crude product was analysed by ^{1}H NMR spectroscopy to determine the conversion to **33** (88%) based on relative integrals of key signals in the product and starting material (Fig. S83). Compound **33** has been previously reported and the spectroscopic data were in agreement.[1]

If the gel was exposed to air for 3 s instead of 5 min, the conversion to **33** was 99% (Fig. S84).

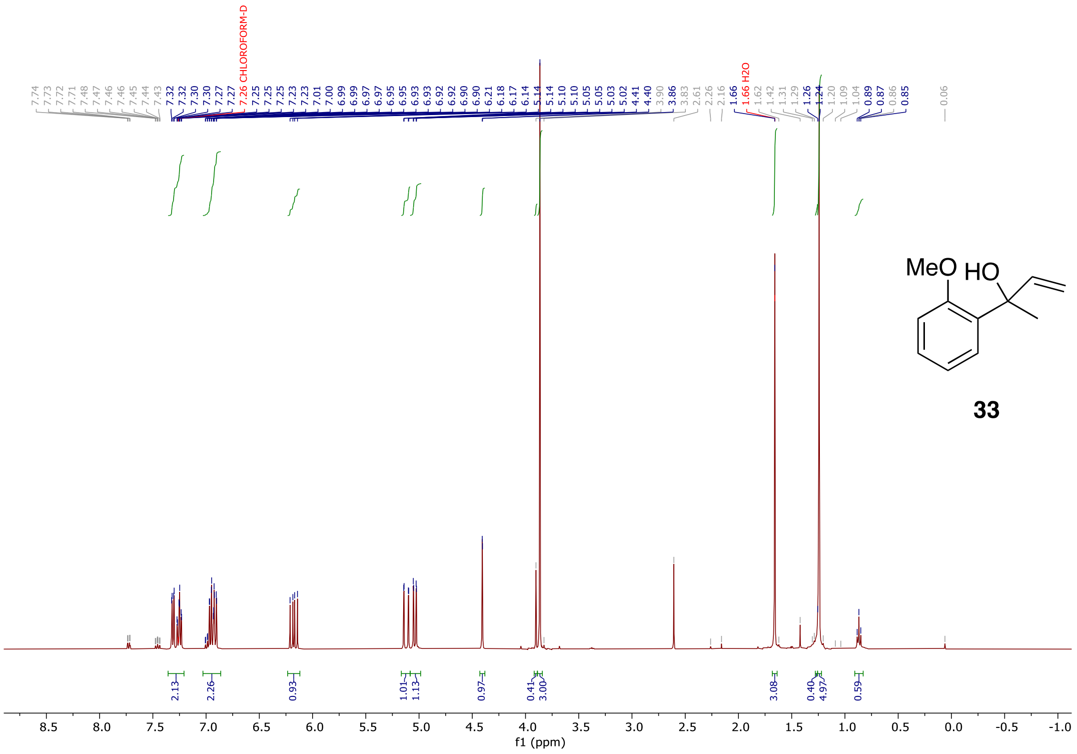

Figure S83. ^{1}H NMR spectrum of the crude product obtained using a vinylMgBr$_{gel}$ block that was exposed to air on a petri dish for 5 min followed by the reaction with 2′-methoxyacetophenone **1** ($CDCl_3$, 298 K, 400 MHz) – Conversion to **33**: 88% – Fig. 4l.

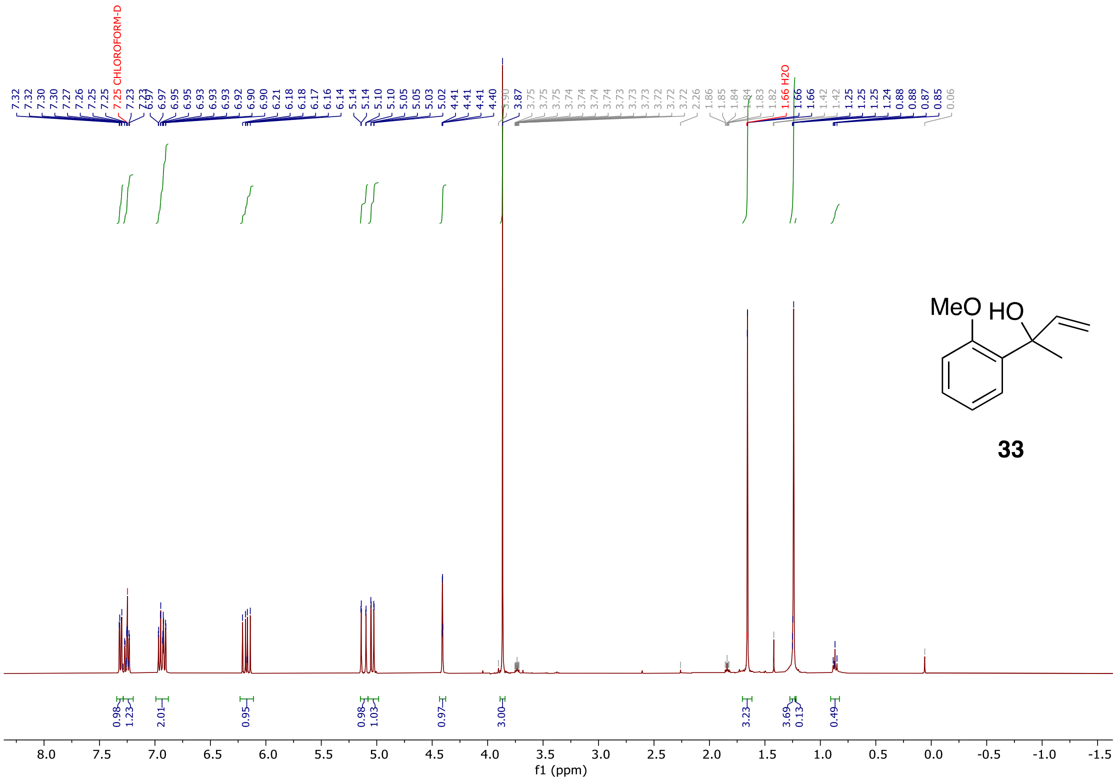

Figure S84. ^{1}H NMR spectrum of the crude product obtained using a vinylMgBr$_{gel}$ block that was exposed to air for 3 s followed by the reaction with 2′-methoxyacetophenone **1** ($CDCl_3$, 298 K, 400 MHz) – Conversion to **33**: 99%.

General procedure L: preparation of robust $PhMgCl_{gel}$ block

A 5 mL vial was dried in the oven and allowed to cool under a nitrogen atmosphere. The vial was charged with the gelator $C_{36}H_{74}$ (100 mg, 0.2 mmol, 6.7% wt/vol), sealed with a rubber septum and flushed with nitrogen via a needle for 5 min. Then, phenylmagnesium chloride (1.5 mL, 1 M in 2-MeTHF, 1.5 mmol) was added through the septum. The vial (kept under a nitrogen atmosphere using a balloon) was carefully heated until all of the gelator had dissolved. The hot hydrosol was quickly transferred under a nitrogen atmosphere via a needle into a 2 mL syringe (previously flushed with nitrogen and pre-heated in the oven). The syringe was immediately placed in iced water for 1 min until the organogel formed. The organomagnesium gel was kept in the syringe under a nitrogen atmosphere prior to use. In order to use the organomagnesium gel, the upper part of the syringe was carefully cut with scissors and the gel block was removed. The organomagnesium gel block was transferred to a separate round bottom flask and the concentration of phenylmagnesium chloride was determined to be 78% of its original value (after exposure to air in a closed vial for 30 min) by titration with propan-2-ol using 1,10-phenanthroline as an indicator.[1]

In a similar way following general procedure L, robust $PhMgCl_{gel}$ blocks were prepared using 10.0, 8.3, 5.7 and 5.0% wt/vol of gelator $C_{36}H_{74}$. All of the formed gels were robust to be transeferred to a round bottom flask and the concentration of phenylmagnesium chloride was determined to be 66-83% of its original value (after exposure to air on a petri dish for 1 min) by titration with propan-2-ol using 1,10-phenanthroline as an indicator.[1]

References

1 Watson, S. C. & Eastham, J. F. Colored indicators for simple direct titration of magnesium and lithium reagents. *J. Organomet. Chem.* **9**, 165-168, doi:10.1016/S0022-328X(00)92418-5 (1967).

2 Vidal, C., García-Álvarez, J., Hernán-Gómez, A., Kennedy, A. R. & Hevia, E. Introducing Deep Eutectic Solvents to Polar Organometallic Chemistry: Chemoselective Addition of Organolithium and Grignard Reagents to Ketones in Air. *Angew. Chem. Int. Ed.* **53**, 5969-5973, doi:10.1002/anie.201400889 (2014).

3 Vidal, C., García-Álvarez, J., Hernán-Gómez, A., Kennedy, A. R. & Hevia, E. Exploiting Deep Eutectic Solvents and Organolithium Reagent Partnerships: Chemoselective Ultrafast Addition to Imines and Quinolines Under Aerobic Ambient Temperature Conditions. *Angew. Chem. Int. Ed.* **55**, 16145-16148, doi: 10.1002/anie.201609929 (2016).

4 Ireland, R. E. & Meissner, R. S. Convenient method for the titration of amide base solutions. *J. Org. Chem.* **56**, 4566−4568, doi:10.1021/jo00014a050 (1991).

5 Rodríguez-Álvarez, M. J., Garcia- Álvarez, J., Uzelac, M, Fairley, M., O'Hara, C. T. & Hevia, E. Introducing Glycerol as a Sustainable Solvent to Organolithium Chemistry: Ultrafast Chemoselective Addition of Aryllithium Reagents to Nitriles under Air and at Ambient Temperature. *Chem. Eur. J.* **24**, 1720-1725, doi:10.1002/chem.201705577 (2018).

6 Chaumont-Olive, P., Rouen, M., Barozzino-Consiglio, G., Ben Abdeladhim, A., Maddaluno, J. & Harrison-Marchand, A. Chiral Lithium Amido Aryl Zincates: Simple and Efficient Chemo- and Enantio-Selective Aryl Transfer Reagents. *Angew. Chem. Int. Ed.* **58**, 3193-3197, doi:10.1002/anie.201813510 (2019).

7 Turan, I. S., Cakmak, F. P. & Sozmen, F. Highly selective fluoride sensing via chromogenic aggregation of a silyloxy-functionalized tetraphenylethylene (TPE) derivative. *Tetrahedron Lett.* **55**, 456-459, doi:10.1016/j.tetlet2013.11.059 (2014).

8 Zhang, G., Bai, R.-X., Li, C.-H., Feng, C.-G. & Lin, G.-Q. Halogenation of 1,1-diarylethylenes by N-halosuccinimides. *Tetrahedron* **75**, 1658-1662, doi:10.1016/j.tet.2018.11.018 (2019).

9 Estévez, M. C., Galve, R., Sánchez-Baeza, F. & Marco, M. P. Direct Competitive Enzyme-Linked Immunosorbent Assay for the Determination of the Highly Polar Short-Chain Sulfophenyl Carboxylates. *Anal. Chem.* **77**, 5283-5293, doi:10.1021/ac0502910 (2005).

10 Chen, W., Ma, L., Paul, A. & Seidel, D. Direct α-C–H bond functionalization of unprotected cyclic amines. *Nature Chemistry* **10**, 165, doi:10.1038/nchem.2871 (2017).

11 Toriyama, F., Cornella, J., Wimmer, L., Chen, T.-G., Dixon, D. D., Creech, G. & Baran, P. S. Redox-Active Esters in Fe-Catalyzed C–C Coupling. *J. Am. Chem. Soc.* **138**, 11132-11135, doi:10.1021/jacs.6b07172 (2016).

12 Sheikh, N. S., Leonori, D., Barker, G., Firth, J. D., Campos, K. R., Meijer, A. J. H. M., O'Brien, P. & Coldham, I. An Experimental and in Situ IR Spectroscopic Study of the Lithiation–Substitution of *N*-Boc-2-phenylpyrrolidine and -piperidine: Controlling the Formation of Quaternary Stereocenters. *J. Am. Chem. Soc.* **134**, 5300-5308, doi:10.1021/ja211398b (2012).

13 Hatano, M., Suzuki, S. & Ishihara, K., Highly Efficient Alkylation to Ketones and Aldimines with Grignard Reagents Catalyzed by Zinc (II) Chloride. *J. Am. Chem. Soc.* **128**, 9998−9999, doi: 10.1021/ja0628405 (2006).

14 Xiong, X., Zhu, R., Huang, L., Chang, S. & Huang, J, Direct *ortho* Arylation of Anisoles via the Formation of Four-Memebered Lithiumcycles/Palladcycles. *Synlett.* **28**, 2046−2050, doi:10.1055/s-0036-1588863 (2017).
